# Climate, Life Form and Family Jointly Control Variation of Leaf Traits

**DOI:** 10.3390/plants8080286

**Published:** 2019-08-14

**Authors:** Hao Zhang, Zhaoxia Zeng, Zhigang Zou, Fuping Zeng

**Affiliations:** 1 Key Laboratory of Agro-Ecological Processes in Subtropical Region, Institute of Subtropical Agriculture, Chinese Academy of Sciences, Changsha 410125, China; 2 Huanjiang Observation and Research Station for Karst Ecosystem, Chinese Academy of Sciences, Huanjiang 547100, China; 3 Guangxi Key Laboratory of Plant Conservation and Restoration Ecology in Karst Terrain, Guangxi Institute of Botany, Guangxi Zhuang Autonomous Region and Chinese Academy of Sciences, Guilin 541006, China

**Keywords:** biome, climate, leaf trait, multivariate analysis, resource use, trade-off

## Abstract

Variation in leaf traits may represent differences in physiological processes and environmental adaptative strategies. Using multivariate analyses, we investigated 13 leaf traits to quantify the trade-off in these traits and the trait–climate/biome relationships based on the China Plant Trait Database, which contains morphometric and physiological character information on 1215 species for 122 sites, ranging from the north to the tropics, and from deserts and grasslands to woodlands and forests. Leaf traits across the dataset of Chinese plants showed different spatial patterns along longitudinal and latitudinal gradients and high variation. There were significant positive or negative correlations among traits; however, with the exception of the leaf ^13^C:^12^C stable isotope ratio, there were no significant correlations between leaf area and other traits. Climate, life form, and family jointly accounted for 68.4% to 95.7% of trait variance. Amongst these forms of variation partitioning, the most important partitioning feature was the family independence of climate and life form (35.6% to 57.2%), while the joint effect of family and climate was 4.5% to 26.2%, and the joint effect of family and life form was 2.4% to 21.6%. The findings of this study will enhance our understanding of the variation in leaf traits in Chinese flora and the environmental adaptative strategies of plants against a background of global climate change, and also may enrich and improve the leaf economics spectrum of China.

## 1. Introduction

Functional traits, as physiological and ecological indicators related to the acquisition, utilization, and maintenance of resources by plants, reflect the response of plants to different environments and the trade-off of physiological or evolutionary adaptation among different functions within plants [1,2,3]. The most common functional traits of plants are morphological, physiological, vegetative and reproductive, aboveground and belowground, and effect and response traits [4,5]. In most studies, these traits have been widely divided into soft and hard traits. Soft traits are generally defined as plant traits that can be readily and rapidly measured, including size of reproductive bodies, shape, leaf area, and tree height [5,6,7]. Compared with soft traits (e.g., propagator size, shape, leaf area, tree height, etc.), hard traits (e.g., leaf photosynthetic rate, plant cold tolerance, negative tolerance, etc.) are difficult to measure but can accurately represent the response of plants to external environment change [8]. Over the past decades, studies on functional traits have been performed at multiple levels, ranging from the individual to the ecosystem [9,10,11,12]. With regard to plant functional traits, the important scientific issues of particular concern in various fields include trade-offs among the different plant functional traits; differences in functional traits among individuals, species, and regions; and the distribution pattern and factors governing variation of functional characters along an environmental gradient (e.g., relationships with climate, topography, and soil nutrients) [13].

The trade-offs between functional traits mainly include trade-offs between leaf traits, leaf and branch and trunk traits, reproductive traits and quantity traits, reproductive traits and seedling leaves, and leaf and root traits [13,14,15]. For example, leaf traits associated with photosynthesis and root traits linked to water and nutrient uptake are closely related [16]. As an important component of plant growth, branch traits are also correlated with leaf and root traits [17,18]. As the only movable stage in plant life history, seed size, traits and quantity are closely related to each other, as well as to the traits of later seedlings [19]. Furthermore, variation in plant functional characters between and within species is an important prerequisite for species coexistence and community development [20,21]. Previous studies on individual plants and functional groups, and at community and ecosystem levels on regional and global scales, have found that the variation in intraspecific and interspecific traits among different communities or functional groups could reflect the response of species to environmental changes and resource competition [2,7]. In addition, the variation in intraspecific traits has been shown to be as important as that in interspecific traits [14], which is of considerable practical significance with regards to guiding research on traits based on communities and ecosystems. 

However, whether the plant traits vary with environmental factors has been continually debated [2,16,22]. The environmental factors domination hypothesis (EFDH) suggests that different species have different patterns of distribution along environmental gradients, which can be attributed to interspecific differences in the morphological and physiological characters of different plant species that are associated with a series of different life strategies [20]. Within different ecosystems and biological communities, these strategies (fast resource acquisition or high resource saving) can be arranged along an axis of basic resources, with life strategies related to resource use located at one end of the axis and those related to resource maintenance located at the other [15]. The resource axis is representative of different relationships with various environmental factors. Moreover, the distribution of plant functional traits is affected by different environmental factors at different scales [23], and the distribution of functional properties in a specific location is often determined by multiple scale-dependent factors [24,25,26,27]. At a global scale or large scale, climatic factors play an important role in determining the distribution of plant functional traits [24,28], whereas at a regional scale, land use and disturbance play a major role [27], and at the local scale, topographic and edaphic factors determine the distribution of trait characters [25]. In contrast, the independent evolution hypothesis (IEH) suggests that allometric growth and tradeoffs between plant functional traits and the resulting life-cycle strategies are independent of climate change and community type [2]. Thus, studies on the relationships between plant functional traits and the environment can not only contribute to gaining a better understanding of existence strategy of different plant communities and species, but also provide an effective evidence to test the two hypothesis [29]. Moreover, although recent studies have examined the variation in traits and trait–environment correlations [21], there has still been little research regarding quantification of the relationships between climatic or phylogenetic factors and plant functional traits. Based on the research mentioned above, here, we assume that EFDH may suitable than the IEH. To test our hypothesis, in the present study, we investigated 13 traits in Chinese plants based on a database of Chinese plant traits [12]. We used multivariate analysis to quantify the traits-environments relationships based on previous dataset and attribute the trait variation by using variance partitioning. The aim of this research is to test EFDH and IEH by using more leaf traits and push the development of the leaf economics spectrum in China.

## 2. Results

### 2.1. Variation in the Leaf Traits of Plants

Both plant (genus and species) and leaf traits showed different spatial patterns along longitudinal and latitudinal gradients (Table S1). The leaf traits of plants within the Chinese plant dataset showed high variation, ranging from 1.67^−07^ to 0.24 m^2^ for LA, 1.64 to 205.59 m^2^/kg for SLA, 0.0049 to 0.61 kg/m^2^ for LMA, 62.09 to 950.57 mg/g for LDMC, 168.95 to 979.36 g/kg for C mass, 0.087 to 122.91 g/kg for N mass, 0.06 to 7.87 g/kg for P mass, 0.12 to 102.53 g/kg for K mass, 0.0054 to 16.02 g/m^2^ for N area, 0.0025 to 1.29 g/m^2^ for P area, 0.003 to 10.93 g/m^2^ for K area, −39.07 to −11.83 for d13C:12C, and −7.40 to 12.14 for d15N:14N (Table 1). The mean values for LA, SLA, LDMC, C mass, N mass, P mass, K mass, N area, P area, K area, d13C:12C, and d15N:14N were 0.0036 m^2^, 20.31 m^2^/kg, 0.07 kg/m^2^, 336.98 mg/g, 436.75 g/kg, 19.59 g/kg, 2.50 g/kg, 14.77 g/kg, 1.32 g/m^2^, 0.15 g/m^2^, 0.83 g/m^2^, −28.56, and −0.26, respectively (Table 1). 

### 2.2. Trade-Off in the Leaf Traits of Plants 

**Figure 1:**
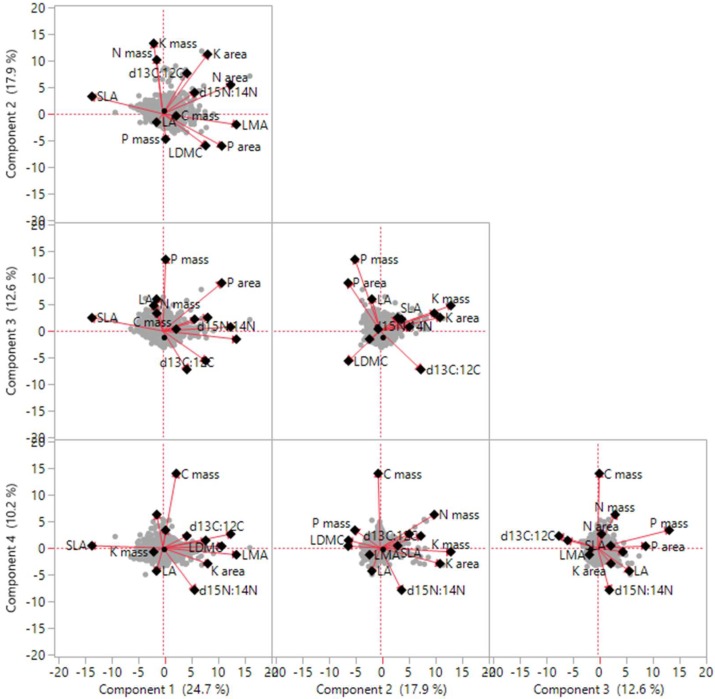
Trait dimensions from the first four principal component (PC) analysis.

Correlation analysis indicated that there were significant positive or negative correlations among traits, whereas with the exception of d13C:12C, no significant correlations were detected between LA and other traits (Figure S1; Table 2). PCA of traits revealed four independent axes of leaf trait variation (Figure 1; Table 3). The first four principal components collectively accounted for 54.53% of the total variation. The first two axes, which were predominantly related to LA, SLA, LMA, LDMC, N mass, and K mass, which together accounted for 32.74% of the total variation, whereas axes 3 and 4 together accounted for 21.79% of total trait variation, indicating that SLA, C mass, N mass, and P mass show close covariance. 

### 2.3. Multi-Factorial Control of Leaf Trait Variation

**Figure 2:**
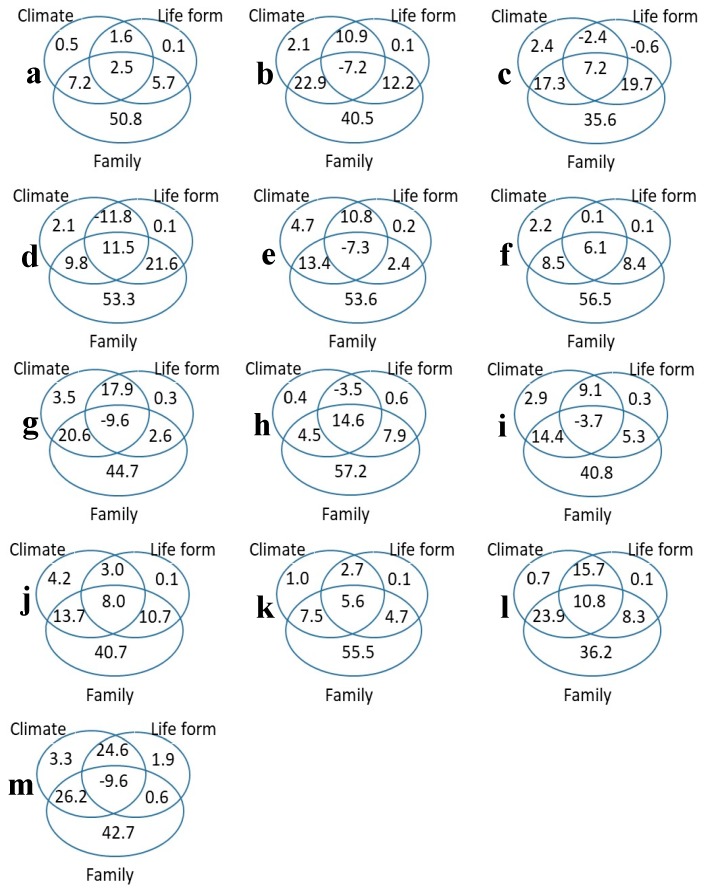
The variance partitioning (%) for all traits considered together, and each trait separately. (**a**) LA, leaf area (m^2^); (**b**) SLA, Specific leaf area (m^2^/kg); (**c**) LMA, Leaf mass per unit area (kg/m^2^); (**d**) LDMC, Leaf dry matter content (mg/g); (**e**) C mass, Leaf carbon content (g/kg); (**f**) N mass, Leaf nitrogen content (g/kg); (**g**) P mass, Leaf phosphorus content (g/kg); (**h**) K mass, Leaf potassium content (g/kg); (**i**) N area, Leaf nitrogen content per unit area (g/m^2^); (**j**) P area, Leaf phosphorus content per unit area (g/m^2^); (**k**) K area, Leaf potassium content per unit area (g/m^2^); (**l**) d13C:12C, The ratio of 13C to 12C stable isotopes in the leaf (unitless); (**m**) d15N:14N, The ratio of 15N to 14N stable isotopes in the leaf (unitless).

Variation partitioning analysis showed the percentage contributions of climate, life form, and family (including intersecting contributions) to the variation in each trait (Figure 2). The trait variation was predominantly determined by climate, life form, and family and interactions among these factors (Figure 2). Among the 13 leaf traits, climate, life form, and family jointly accounted for 68.4% to 95.7% of trait variance. The most important feature of the partitioning was the family independence of climate and life form (35.6% to 57.2%), whereas the joint effect of family and climate was 4.5% to 26.2% and the joint effect of family and life form was 2.4% to 21.6%. In other words, this means that the most important feature was taxonomic (influenced by family), independent of the effects of climate or life-form, with the interaction term between family and other traits detected to have a much smaller effect.

## 3. Discussion

### 3.1. Variation in Leaf Traits and Its Control

In the present study, we found that 13 leaf traits of plants growing in China showed different patterns of variation (Table S1), which may be influenced by the natural and historical conditions and the evolution of vegetation [18]. At species and sites levels, predictions of leaf traits except for LDMC and SLA based on the multivariate analysis were similar with the world−wide prediction of traits [21]. For these multiple factors, previous studies have shown that interspecific variation plays a dominant role in the variation of plant functional traits, accumulating evidence indicates that intraspecific variation, which can account for 28% to 52% of total trait variation [30,31], should not be ignored. We found that the family (interspecific or intraspecific) variation in plant traits ranged from 35.6% to 57.2%, which supports our hypothesis and is consistent with the findings of some previous studies [32,33]. In addition, with regards to the leaf traits of more than 1100 plants types along a sample belt of the North–South Transect of Eastern China (NSTEC), it has previously been demonstrated that the latitudinal variability of leaf morphological attributes differs at the species and community levels [34]. 

Plant traits are determined by both genetic factors and environmental conditions [35], and among species with different genetic diversity backgrounds, there was considerable interspecific variation among traits, particular with respect to leaf area and the leaf 15N to 14N stable isotope ratio [36]. In contrast, variations in the 13C to 12C stable isotope ratio; specific leaf area; leaf dry matter content; leaf mass per unit area; leaf carbon, nitrogen, phosphorus, and potassium contents; and leaf nitrogen, phosphorus, and potassium content per unit area were relatively small and were relatively stable among the species surveyed [37]. Nevertheless, plants have, of necessity, adapted to their surrounding environments over the long term, and the range of variation in their traits differs according to environment and habitat [6]. In particular, in regions characterized by high atmospheric nitrogen deposition, variation in leaf traits of plants have been found to be more complex and diverse, and under such conditions, leaf nitrogen isotopes may show greater variation and unpredictability [38].

In the present study, we also found that leaf traits were mainly affected by species groups (genetic background), and to a lesser extent by life form and climate (Figure 2). Given that the interaction of life form and climate is also associated with the species classification units, plant system development or its evolution and classification background can have a considerable effect on trait differentiation, sometimes to greater extent than the influence of environmental factors [39,40]. However, we found that the explanatory power of the effect of species groups on the variation in leaf mass per unit area and the leaf 13C to 12C stable isotope ratio was relatively low, and thus the underlying mechanisms need further study.

### 3.2. Covariation of Different Leaf Traits

Plant growth and long−term adaptation to the environment are influenced by physiological, phylogenetic, and environmental factors, and plant traits are correlated to some extent [7,41], eventually forming a series of optimal combinations of functional traits that are adapted to a specific environment [42,43]. On the basis of a comprehensive analysis of multiple trait values of a large number of species, a previous study found that there were different degrees of correlation between various plant traits at the global or regional scale, and that plant functional traits could be classified into four principal dimensions, with the same dimension also having a high degree of correlation among functional traits [42]. Furthermore, a large number of studies have also shown that the correlation between plant functional traits can differ according to research scales. For example, LA and SLA have been found to show significant positive correlation at global and regional scales [10,22], whereas no or negative correlations between LA and SLA have been detected [43]. In the present study, however, we were able to detect any significant correlation between SLA and LA at the whole dataset level (Table 2), which is consistent with the findings of some of previous studies [21].

In general, plants with high leaf nutrient content, particularly N content, typically have high photosynthetic capacity and respiratory consumption to facilitate adaptation to the environment via rapid nutrient cycling, whereas those with lower leaf nutrient content have lower photosynthetic capacity and survive through rapid nutrient cycling [2,7]. In the present study, we found that nutrient content per unit area was significantly and positively correlated with plant LMA and LDMC (Table 2). With an increase in LDMC, there is a concomitant thickening of leaves, which leads to an increase of dry matter per unit area, and thus an increase of nutrient content per unit area. Plants with higher LMA and LDMC tend to have stronger resistance to stress and maintain growth by accumulating captured resources [44]. Moreover, although the unit mass of dry matter allocated to leaf area is reduced with an increase in LMA and LDMC, this may not affect the nutrient content of blade unit mass [38]. However, our data showed that the mass N, P, and K was reduced with an increase in LMA and LDMC (Table 2). In this regard, it indicates that leaf K content decreases with the decrease of leaf area distributed per unit dry matter. Previous studies have shown that potassium can obviously improve the absorption and utilization of nitrogen, promote photosynthesis, and enhance stress resistance of plants [45,46,47]. With increasing LMA and LDMC, leaf blade thickness increases and the leaf area corresponding to the blade per unit mass decreases, which to a certain extent reduces the number of stomata corresponding to the blade per unit mass, thereby reducing K mass. When N area and P area are constant, N mass and P mass will decrease with an increase in blade thickness (an increase LMA and LDMC) [2,48]. 

N, P, and K are all essential elements affecting plant growth and development. As shown in Table 2, the relationship between nutrient contents in leaves based on area was more significant than that based on mass. Moreover, the significant relationships between N and P based on area and mass were higher than those between N and K and P and K, which is consistent with results obtained at a global scale [5,15]. These observations can probably be attributed to the fact that N and P are directly involved in the synthesis of all stable plant structural materials, while K is not [49]. 

### 3.3. Leaf Economics Spectrum in China

Although Wright et al. [2] collected plant trait data from most regions of the world, they also pointed out that there had been relatively limited study on the leaf economics spectrum in China. However, theoretical and applied aspects of the leaf economics spectrum are still in need of further study. Studies on the relationship between the leaf economics spectrum in China and global environmental factors are still insufficient, and there have been even fewer studies that have used the relationships of trait data to construct dynamic models of vegetation–climate–land change [48,50]. The data presented in the present study revealed the variations and trade−offs in leaf traits across different climates and biomes based on a Chinese plant trait database, which may enhance our understanding of the leaf economics spectrum in China. 

As a research idea and method, global leaf economics spectrum theory, which describes plant functional traits and their relationships, can provide a new perspective for explaining and addressing the many challenging ecological problems facing China today. Xu et al. [51] proposed that the vulnerability and adaptability of China’s ecosystems against a background of global climate change could be discussed through assessing the importance of plant functional traits in terms of resource utilization. Under the pressure of environmental change and selection, plant traits will adjust to different degrees in order to adapt to environmental stress or resource limitation, and the traits of the economics spectrum of different species will show different types of response to the gradient of environmental change [52,53,54]. Studies that have sought to clarify different vegetation types, the typical economic state of the response spectrum of species, and analysis of the ecosystem service function of specific flora [55] will contribute to supplementing and enhancing the law of response of the leaf economics spectrum to global change. Meanwhile, we are aware that the representativeness and completeness of the data in this study are still limited, which is mainly reflected in the lack of data on plant traits in extreme environments, remote and backward areas, and the lack of data on species that are difficult to measure, such as needle leaves and bryophytes. Extensive leaf traits data collection including more research sites, species, leaf structure and anatomy traits is still needed in future research. Moreover, based on the investigation data of long−term fixed monitoring sites, future research also should focus on analyzing the inherent links among functional traits, plants, and ecosystems, as well as establishing theoretical models of multi−scale and multi−dimensional vegetation economics spectra (VES) to promote biodiversity conservation and ecosystem management, such as formulating strategies of biodiversity conservation, schemes design of vegetation restoration in ecologically fragile areas, setting rules and ecological environment planning schemes according to the indicative role of functional traits.

## 4. Materials and Methods 

### 4.1. Plant Trait Database

Data on plant traits collated from previous research were extracted from the Plant Trait Database of China [21,56], which includes morphometric and physiological character information for 122 sites ranging from the north to the tropics, and from forests and woodlands to grasslands and deserts. The sample sites span an extremely large range of moisture and temperature regimes. The research area includes the Northeast China Transect, Northwest China Transect, and North−South Transect of Eastern China. At each site, data were collected based on a dominant individual species in different ecosystem types. The ecosystems include trees, small trees, lianas, shrubs, forbs, graminoids, bamboos, herbaceous, climbers, geophytes and pteridophytes. The previous used different sampling strategies in the collection of different types of trait data. Most of the fieldwork subprojects included multiple sites. All family attributions of plants were checked and verified, and these with uncertain family attributions were not included in this database. Collectively, this dataset contains information on the vegetation type, leaf traits, climate, and life form relating to 1215 species. Here, all these data were included in our analysis. 

In this study, we selected 13 functional traits in the database that were easier to measure, relatively stable and easier to compare with other studies. These leaf traits selected were as follows: leaf area (LA, m^2^), specific leaf area (SLA, m^2^/kg), leaf mass per unit area (LMA, kg/m^2^), leaf dry matter content (LDMC, mg/g), leaf carbon content (C mass, g/kg), leaf nitrogen content (N mass, g/kg), leaf phosphorus content (P mass, g/kg), leaf potassium content (K mass, g/kg), leaf nitrogen content per unit area (N area, g/m^2^), leaf phosphorus content per unit area (P area, g/m^2^), leaf potassium content per unit area (K area, g/m^2^), the 13C to 12C stable isotope ratio of leaves (d13C:12C, unitless), and the 15N to 14N stable isotope ratio of leaves (d15N:14N, unitless).

### 4.2. Climatic, Vegetation, Family, and Life Form Variables

The climates in this dataset includes temperate continental climate, monsoon climate of medium latitudes, subtropical monsoon climate, tropical monsoon climate, and alpine plateau climate. The diverse climates in China are characterized by a wide range of humidity and temperatures, and in the present study, we firstly selected three bioclimatic variables that fully represent the main plant community structure and composition in the different regions of China [21,56]: the index of heat accumulation during the growing season, which includes photosynthetic active radiation (PAR0, the period when daily temperature above 0 °C); the mean daily temperature of the thermal growth season(mGDD0); and the ratio of average annual precipitation to annual equilibrium evapotranspiration (water index, MI) [21,57]. The data used to calculate these bioclimatic variables were mainly obtained from 1814 weather stations (740 from 1971 to 2000 and 1074 from 1981 to 1990), using ANUSPLIN v.4.37 to interpolate elevation as a covariant to 1km resolution [58]. In order to fully test the effect of climate on the leaf traits, we also selected other climatic variables, including mean annual temperature (MAT, °C), mean monthly precipitation (MMP, mm), mean annual precipitation (MAP, mm), actual evapotranspiration/equilibrium (alpha or α, unitless), growing degree days above 0 °C (GDD0, °days), photosynthetically active radiation per day during the growing season and at temperatures above 0 °C (mPAR0, mol photon m^−2^), the timing of peak precipitation (Prec timing, a monthly vector where January 1st and December 31th is set to an angle of 0° and 12°, respectively), and the seasonality of precipitation (Prec season, where 0 and 1 denote that precipitation is equally distributed in each month of the year and concentrated in 1 month of the year, respectively, unitless). 

The vegetation variables in the trait dataset relate to fundamental vegetation types classified according to the vegetation map of China, clustered vegetation types defined by Wang et al. [12], and biome classification determined by the dominant plant functional types. In detail, the biomes include alpine tundra and steppe, temperate steppe, temperate broadleaf deciduous forest, temperate deciduous woodland, temperate needleleaf forest, temperate shrubland, temperate broadleaf deciduous forest, temperate evergreen needleleaf forest, temperate grassland, temperate desert, subtropical deciduous broadleaf forest, subtropical mixed forest, subtropical evergreen broadleaf forest, tropical shrub, tropical grass, and temperate crops. Moreover, the species family (genus and species), life form (e g., trees, small trees, lianas, shrubs, forbs and graminoids), plant phenology, leaf phenology, and leaf type were also included in this dataset. The plants in this dataset included 614 genus and 1215 species, respectively.

### 4.3. Statistical Analysis

SAS JMP 14.1 software (SAS Inc., North Carolina, CA, USA, 2018) was used to compare the different traits using analysis of variance (ANOVA). Pearson correlations between leaf traits across Chinese plants were calculated in JMP. After traits had been log−transformed, the covariance matrix among traits was determined using principal component analysis (PCA) [59]. The effects of climate, family, and life form on the leaf traits were identified by variation partitioning [18,60]. The method we used here was the Legendre method, which explicitly accounts for correlations between groups by distinguishing unique and overlapping contributions from each group [56].

## Figures and Tables

**Table 1:** Summary statistics of functional traits across China’s different climates and biomes.

Functional Traits	Mean	Minimum	Maximum	Standard Deviation	Variable Coefficient
LA	0.0036	1.67^−07^	0.24012	0.0098	2.7222
SLA	20.3092	1.6420	205.6915	12.9413	0.6372
LMA	0.0712	0.0049	0.6090	0.0546	0.7669
LDMC	336.9785	62.0915	950.5650	116.2379	0.3449
C mass	436.7534	168.9521	979.3636	85.6143	0.1960
N mass	19.5915	0.0870	122.9118	8.9276	0.4557
P mass	2.4990	0.0600	7.8705	1.2507	0.5005
K mass	14.7673	0.1210	102.5344	10.3826	0.7031
N area	1.3227	0.0054	16.0167	0.9466	0.7157
P area	0.1487	0.0025	1.2868	0.1086	0.7303
K area	0.8250	0.0030	10.9283	0.7188	0.8713
d13C:12C	−28.5584	−39.0705	−11.8300	4.4495	−0.1558
d15N:14N	−0.2624	−7.4005	12.1350	2.8134	−10.7218

LA, leaf area (m^2^); SLA, Specific leaf area (m^2^/kg); LMA, Leaf mass per unit area (kg/m^2^); LDMC, Leaf dry matter content (mg/g); C mass, Leaf carbon content (g/kg); N mass, Leaf nitrogen content (g/kg); P mass, Leaf phosphorus content (g/kg); K mass, Leaf potassium content (g/kg); N area, Leaf nitrogen content per unit area (g/m^2^); P area, Leaf phosphorus content per unit area (g/m^2^); K area, Leaf potassium content per unit area (g/m^2^); d13C:12C, The ratio of 13C to 12C stable isotopes in the leaf (unitless); d15N:14N, The ratio of 15N to 14N stable isotopes in the leaf (unitless).

**Table 2:** Pearson correlations efficient and correlation significance level between functional traits across China’s different climates and biomes.

	LA	SLA	LMA	LDMC	C Mass	N Mass	P Mass	K Mass	N Area	P Area	K Area	d13C:12C	d15N:14N
LA		0.2195	0.1841	0.4822	0.4698	0.5431	0.0685	0.4745	0.1463	0.1263	0.8353	**<0.0001**	0.1828
SLA	0.054		**<0.0001**	**<0.0001**	**0.0026**	**<0.0001**	0.2830	**<0.0001**	**<0.0001**	**<0.0001**	**<0.0001**	**0.0007**	**<0.0001**
LMA	−0.059	−0.617		**<0.0001**	0.2980	**<0.0001**	**0.0105**	**<0.0001**	**<0.0001**	**<0.0001**	**<0.0001**	**0.0070**	**0.0100**
LDMC	−0.031	−0.482	0.281		**0.0021**	**<0.0001**	0.0592	**<0.0001**	**0.0007**	**<0.0001**	0.8982	**<0.0001**	0.2827
C mass	−0.032	−0.133	-0.046	0.135		**<0.0001**	**0.0118**	0.5186	**<0.0001**	**0.0287**	0.4322	**0.0171**	**<0.0001**
N mass	−0.027	0.203	−0.247	−0.233	0.181		0.7016	**<0.0001**	**<0.0001**	**<0.0001**	**0.0006**	**0.0004**	**0.0002**
P mass	0.080	0.047	−0.113	−0.083	0.111	0.017		0.8934	**0.0362**	**<0.0001**	**0.0487**	**<0.0001**	0.8104
K mass	0.032	0.257	−0.222	−0.335	−0.029	0.335	0.006		0.1288	**<0.0001**	**<0.0001**	**<0.0001**	0.5534
N area	−0.064	−0.492	0.694	0.149	0.184	0.365	−0.092	0.067		**<0.0001**	**<0.0001**	**<0.0001**	**<0.0001**
P area	0.067	−0.485	0.556	0.297	0.096	−0.187	0.630	−0.183	0.367		**<0.0001**	**0.0050**	**<0.0001**
K area	−0.009	−0.276	0.363	0.006	−0.035	0.151	−0.087	0.711	0.495	0.179		**<0.0001**	**<0.0001**
d13C:12C	−0.225	−0.148	0.119	0.175	0.105	0.154	−0.258	0.197	0.214	−0.124	0.326		**0.0002**
d15N:14N	0.059	−0.237	0.113	0.047	−0.247	0.163	−0.011	0.026	0.283	0.213	0.275	0.161	

LA, leaf area (m^2^); SLA, Specific leaf area (m^2^/kg); LMA, Leaf mass per unit area (kg/m^2^); LDMC, Leaf dry matter content (mg/g); C mass, Leaf carbon content (g/kg); N mass, Leaf nitrogen content (g/kg); P mass, Leaf phosphorus content (g/kg); K mass, Leaf potassium content (g/kg); N area, Leaf nitrogen content per unit area (g/m^2^); P area, Leaf phosphorus content per unit area (g/m^2^); K area, Leaf potassium content per unit area (g/m^2^); d13C:12C, The ratio of 13C to 12C stable isotopes in the leaf (unitless); d15N:14N, The ratio of 15N to 14N stable isotopes in the leaf (unitless).

**Table 3:** Trait loadings, eigenvalues, and the percentage of trait variation explained by Principal components analysis (PCA).

Leaf Traits	PC1	PC2	PC3	PC4
LA	**0.95**	−0.01	0.05	−0.19
SLA	**0.84**	0.17	0.00	**0.43**
LMA	−0.19	**0.93**	−0.02	0.18
LDMC	**0.38**	**0.88**	−0.01	0.02
C mass	−0.13	−0.01	**0.96**	0.04
N mass	**0.51**	−0.02	**0.78**	−0.14
P mass	−0.05	0.15	−0.03	**0.95**
K mass	**−0.64**	0.00	−0.04	0.23
N area	0.16	−0.13	0.02	−0.09
P area	0.12	0.07	0.05	0.11
K area	0.07	−0.03	0.07	0.12
d13C:12C	−0.04	0.02	0.04	−0.02
d15N:14N	0.08	0.17	−0.15	0.06
Eigenvalue	2.53	1.73	1.57	1.26
Explained (%)	19.43	13.31	12.09	9.70
Cumulative (%)	19.43	32.74	44.83	54.53

LA, leaf area (m^2^); SLA, Specific leaf area (m^2^/kg); LMA, Leaf mass per unit area (kg/m^2^); LDMC, Leaf dry matter content (mg/g); C mass, Leaf carbon content (g/kg); N mass, Leaf nitrogen content (g/kg); P mass, Leaf phosphorus content (g/kg); K mass, Leaf potassium content (g/kg); N area, Leaf nitrogen content per unit area (g/m^2^); P area, Leaf phosphorus content per unit area (g/m^2^); K area, Leaf potassium content per unit area (g/m^2^); d13C:12C, The ratio of 13C to 12C stable isotopes in the leaf (unitless); d15N:14N, The ratio of 15N to 14N stable isotopes in the leaf (unitless).

## Supplementary Materials

The following are available online at https://www.mdpi.com/2223-7747/8/8/286/s1, Table S1. The information of plant genus, plant species, biome classification, life form, longitude and latitude in this research. Figure S1 Scatterplot matrix of functional traits across China’s plants.
