# Supplementary material for: Climate, Life Form and Family Jointly Control Variation of Leaf Traits
Source: Plants (Basel). 2019 Aug 14;8(8):286. doi: 10.3390/plants8080286 (PMC6724092; doi:10.3390/plants8080286)
Supplement: Supplementary file 1 [file plants-08-00286-s001.pdf]

Climate, Life Form and Family Jointly Control Variation of Leaf Traits

Hao Zhang [1,2,3], Zhaoxia Zeng [1,2], Zhigang Zou [1,2] and Fuping Zeng [1,2,*]

[1] Key Laboratory of Agro-Ecological Processes in Subtropical Region, Institute of Subtropical Agriculture, Chinese Academy of Sciences, Changsha 410125, China
[2] Huanjiang Observation and Research Station for Karst Ecosystem, Chinese Academy of Sciences, Huanjiang 547100, China
[3] Guangxi Key Laboratory of Plant Conservation and Restoration Ecology in Karst Terrain, Guangxi Institute of Botany, Guangxi Zhuang Autonomous Region and Chinese Academy of Sciences, Guilin 541006, China
* Correspondence: fpzeng@isa.ac.cn; Tel.: +86-731-8461-9701

Figure S1 Scatterplot matrix of functional traits across China's plants. LA, leaf area (m^2); SLA, Specific leaf area (m^2/kg); LMA, Leaf mass per unit area (kg/m^2); LDMC, Leaf dry matter content (mg/g); C mass, Leaf carbon content (g/kg); N mass, Leaf nitrogen content (g/kg); P mass, Leaf phosphorus content (g/kg); K mass, Leaf potassium content (g/kg); N area, Leaf nitrogen content per unit area (g/m^2); P area, Leaf phosphorus content per unit area (g/m^2); K area, Leaf potassium content per unit area (g/m^2); d13C:12C, The ratio of 13C to 12C stable isotopes in the leaf (unitless); d15N:14N, The ratio of 15N to 14N stable isotopes in the leaf (unitless). The red line and red circle represent fit line and density ellipses, respectively.

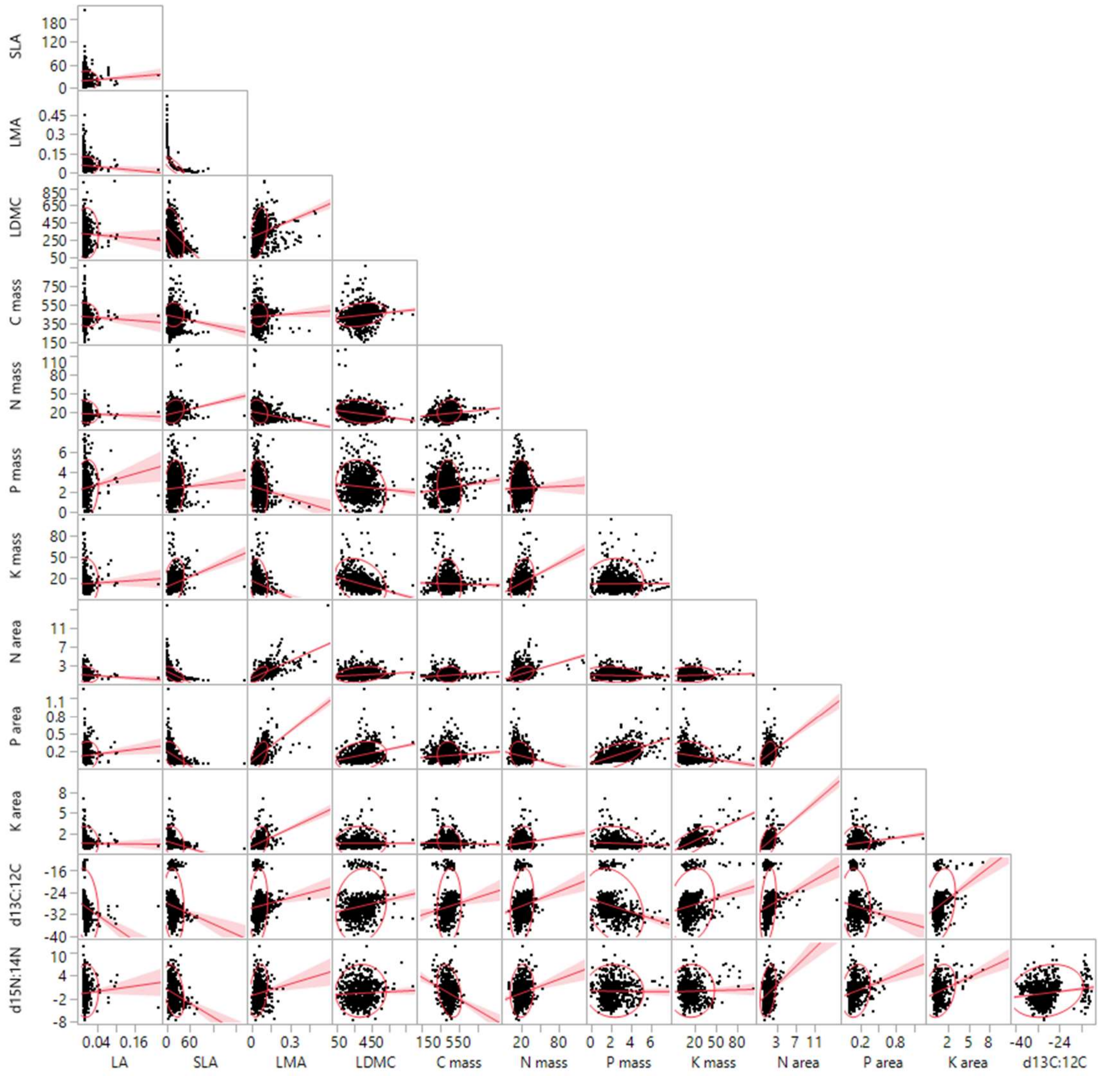

SLA
LMA
LDMC
C mass
N mass
P mass
K mass
N area
P area
K area
d13C:12C
d15N:14N
LA
SLA
LMA
LDMC
C mass
N mass
P mass
K mass
N area
P area
K area
d13C:12C

Table S1. The information of plant genus, plant species, biome classification, life form, longitude and latitude in this research.

Genus	Species	Biome classification	Life form	Latitude (°)	Longitude (°)
Allium	senescens	steppe	geophyte	42.875	118.485
Artemisia	frigida	steppe	erect dwarf shrub	42.875	118.485
Artemisia	sacrorum	steppe	erect dwarf shrub	42.875	118.485
Artemisia	sacrorum var glauca	steppe	erect dwarf shrub	42.875	118.485
Astragalus	galactites	steppe	forb	42.875	118.485
Astragalus	scaberrimus	steppe	forb	42.875	118.485
Cleistogenes	squarrosa	steppe	graminoid	42.875	118.485
Euphorbia	esula	steppe	forb	42.875	118.485
Euphorbia	humifusa	steppe	forb	42.875	118.485
Lespedeza	davurica	steppe	erect dwarf shrub	42.875	118.485
Leymus	chinensis	steppe	graminoid	42.875	118.485
Polygala	tenuifolia	steppe	forb	42.875	118.485
Potentilla	discolor	steppe	forb	42.875	118.485
Scutellaria	baicalensis	steppe	forb	42.875	118.485
Stipa	krylovii	steppe	graminoid	42.875	118.485
Thymus	mongolicus	steppe	erect dwarf shrub	42.875	118.485
Unidentified forb 1		steppe	forb	42.875	118.485
Unidentified grass 1		steppe	graminoid	42.875	118.485
Agropyron	cristatum	steppe	graminoid	43.645	119.025
Anemarrhena	asphodeloides	steppe	forb	43.645	119.025
Artemisia	frigida	steppe	erect dwarf shrub	43.645	119.025
Artemisia	sacrorum	steppe	erect dwarf shrub	43.645	119.025
Artemisia	sacrorum var glauca	steppe	erect dwarf shrub	43.645	119.025

Artemisia	scoparia	steppe	forb	43.645	119.025
Astragalus	galactites	steppe	forb	43.645	119.025
Astragalus	scaberrimus	steppe	forb	43.645	119.025
Caragana	microphylla	steppe	low to high shrub	43.645	119.025
Cleistogenes	squarrosa	steppe	graminoid	43.645	119.025
Cynanchum	thesioides	steppe	forb	43.645	119.025
Delphinium	grandiflorum	steppe	forb	43.645	119.025
Echinops	sp	steppe	forb	43.645	119.025
Erodium	stephanianum	steppe	forb	43.645	119.025
Euphorbia	esula	steppe	forb	43.645	119.025
Euphorbia	humifusa	steppe	forb	43.645	119.025
Glycyrrhiza	uralensis	steppe	low to high shrub	43.645	119.025
Haplophyllum	dauricum	steppe	forb	43.645	119.025
Heteropappus	altaicus	steppe	forb	43.645	119.025
Lespedeza	davurica	steppe	forb	43.645	119.025
Melilotoides	ruthenica	steppe	forb	43.645	119.025
Oxytropis	sp	steppe	forb	43.645	119.025
Potentilla	tanacetifolia	steppe	forb	43.645	119.025
Saussurea	parviflora	steppe	forb	43.645	119.025
Scutellaria	baicalensis	steppe	forb	43.645	119.025
Scutellaria	scordifolia	steppe	forb	43.645	119.025
Serratula	yamatsutana	steppe	forb	43.645	119.025
Sesamum	indicum	steppe	forb	43.645	119.025
Stellaria	chamaejasme	steppe	forb	43.645	119.025
Stipa	krylovii	steppe	graminoid	43.645	119.025
Taraxacum	mongolicum	steppe	forb	43.645	119.025

Thalictrum	squarrosum var supradecompositum	steppe	forb	43.645	119.025
Thymus	mongolicus	steppe	erect dwarf shrub	43.645	119.025
Tragus	racemosus	steppe	graminoid	43.645	119.025
Tribulus	terrestris	steppe	forb	43.645	119.025
Unidentified geophyte		steppe	geophyte	43.645	119.025
unidentified scroph		steppe	forb	43.645	119.025
Unidentified semi-rosette forb 1		steppe	forb	43.645	119.025
Unidentified stoloniferous grass		steppe	graminoid	43.645	119.025
Acer	mono	steppe	tree	43.025	129.775
Astilbe	chinensis	steppe	forb	43.025	129.775
Betula	utilis	steppe	tree	43.025	129.775
Carex	pediformis	steppe	graminoid	43.025	129.775
Clematis	sp	steppe	climber	43.025	129.775
Corylus	heterophylla	temperate broadleaf deciduous forest	tree	43.025	129.775
Dioscorea	nipponica	temperate broadleaf deciduous forest	climber	43.025	129.775
Euonymus	alatus	temperate broadleaf deciduous forest	low to high shrub	43.025	129.775
Fraxinus	rhynchophylla	temperate broadleaf deciduous forest	tree	43.025	129.775
Hemerocallis	middendorffii	temperate broadleaf deciduous forest	graminoid	43.025	129.775
Lespedeza	bicolor	temperate broadleaf deciduous forest	low to high shrub	43.025	129.775

Lonicera	chrysantha	temperate broadleaf deciduous forest	low to high shrub	43.025	129.775
Philadelphus	tenuifolius	temperate broadleaf deciduous forest	low to high shrub	43.025	129.775
Populus	davidiana	temperate broadleaf deciduous forest	tree	43.025	129.775
Pteridium	aquilinum	temperate broadleaf deciduous forest	pteridophyte	43.025	129.775
Ribes	amurensis	temperate broadleaf deciduous forest	low to high shrub	43.025	129.775
Rosa	sp	temperate broadleaf deciduous forest	low to high shrub	43.025	129.775
Streptopus	streptopoides	temperate broadleaf deciduous forest	climber	43.025	129.775
Thalictrum	tuberiferum	temperate broadleaf deciduous forest	forb	43.025	129.775
Tilia	amurensis	temperate broadleaf deciduous forest	tree	43.025	129.775
Tilia	mandshurica	temperate broadleaf deciduous forest	tree	43.025	129.775
Ulmus	davidiana var japonica	temperate broadleaf deciduous forest	tree	43.025	129.775
Urtica	angustifolia	temperate broadleaf deciduous forest	forb	43.025	129.775
Viola	sp	temperate broadleaf deciduous forest	forb	43.025	129.775

Artemisia	sp	temperate broadleaf deciduous forest	forb	42.985	130.075
Artemisia	sylvatica	temperate broadleaf deciduous forest	forb	42.985	130.075
Asparagus	dauricus	temperate broadleaf deciduous forest	forb	42.985	130.075
Calamagrostis	epigejos	temperate broadleaf deciduous forest	graminoid	42.985	130.075
Carex	sp	temperate broadleaf deciduous forest	graminoid	42.985	130.075
Dioscorea	nipponica	temperate broadleaf deciduous forest	climber	42.985	130.075
Securinega	suffruticosa	temperate broadleaf deciduous forest	tree	42.985	130.075
Fraxinus	rhynchophylla	temperate broadleaf deciduous forest	tree	42.985	130.075
Hemerocallis	middendorffii	temperate broadleaf deciduous forest	graminoid	42.985	130.075
Ixeris	chinensis	temperate broadleaf deciduous forest	forb	42.985	130.075
Lathyrus	davidii	temperate broadleaf deciduous forest	forb	42.985	130.075
Phlomis	maximowiczii	temperate broadleaf deciduous forest	forb	42.985	130.075
Pinus	tabuliformis	temperate broadleaf deciduous forest	tree	42.985	130.075

Polygonatum	oderatum	temperate broadleaf deciduous forest	forb	42.985	130.075
Prunus	padus	temperate broadleaf deciduous forest	tree	42.985	130.075
Pteridium	aquilinum	temperate broadleaf deciduous forest	pteridophyte	42.985	130.075
Quercus	mongolica	temperate broadleaf deciduous forest	tree	42.985	130.075
Rhamnus	schneideri	temperate broadleaf deciduous forest	low to high shrub	42.985	130.075
Rubia	sylvatica	temperate broadleaf deciduous forest	forb	42.985	130.075
Syneilesis	aconitifolia	temperate broadleaf deciduous forest	forb	42.985	130.075
Thalictrum	tuberiferum	temperate broadleaf deciduous forest	forb	42.985	130.075
Ulmus	davidiana var japonica	temperate broadleaf deciduous forest	tree	42.985	130.075
Urtica	angustifolia	temperate broadleaf deciduous forest	forb	42.985	130.075
Vicia	amoena	temperate broadleaf deciduous forest	climber	42.985	130.075
Viola	variegata	temperate broadleaf deciduous forest	forb	42.985	130.075
Acer	ginnala	temperate broadleaf deciduous forest	tree	43.295	131.145

Adenophora	tetraphylla	temperate broadleaf deciduous forest	forb	43.295	131.145
Aegopodium	alpestre	temperate broadleaf deciduous forest	forb	43.295	131.145
Agrimonia	pilosa	temperate broadleaf deciduous forest	forb	43.295	131.145
Asparagus	sp	temperate broadleaf deciduous forest	ND	43.295	131.145
Astilbe	chinensis	temperate broadleaf deciduous forest	forb	43.295	131.145
Betula	albosinensis	temperate broadleaf deciduous forest	tree	43.295	131.145
Betula	platyphylla	temperate broadleaf deciduous forest	tree	43.295	131.145
Bromus	inermis	temperate broadleaf deciduous forest	forb	43.295	131.145
Campanula	glomerata	temperate broadleaf deciduous forest	forb	43.295	131.145
Carex	pediformis	temperate broadleaf deciduous forest	graminoid	43.295	131.145
Carex	sp	temperate broadleaf deciduous forest	graminoid	43.295	131.145
Corylus	heterophylla	temperate broadleaf deciduous forest	tree	43.295	131.145
Crataegus	sp	temperate broadleaf deciduous forest	tree	43.295	131.145

Dioscorea	nipponica	temperate broadleaf deciduous forest	climber	43.295	131.145
Acanthopanax	sessiliflorus	temperate broadleaf deciduous forest	low to high shrub	43.295	131.145
Equisetum	hyemale	temperate broadleaf deciduous forest	pteridophyte	43.295	131.145
Euonymus	alatus	temperate broadleaf deciduous forest	low to high shrub	43.295	131.145
Fraxinus	rhynchophylla	temperate broadleaf deciduous forest	tree	43.295	131.145
Fraxinus	mandshurica	temperate broadleaf deciduous forest	tree	43.295	131.145
Geum	sp	temperate broadleaf deciduous forest	forb	43.295	131.145
Larix	olgensis	temperate broadleaf deciduous forest	tree	43.295	131.145
Lespedeza	bicolor	temperate broadleaf deciduous forest	low to high shrub	43.295	131.145
Lonicera	chrysantha	temperate broadleaf deciduous forest	low to high shrub	43.295	131.145
Maackia	amurensis	temperate broadleaf deciduous forest	tree	43.295	131.145
Mukdenia	rossii	temperate broadleaf deciduous forest	forb	43.295	131.145
Onoclea	sensibilis	temperate broadleaf deciduous forest	pteridophyte	43.295	131.145

Cacalia	hastata	temperate broadleaf deciduous forest	forb	43.295	131.145
Phellodendron	amurense	temperate broadleaf deciduous forest	tree	43.295	131.145
Phragmites	australis	temperate broadleaf deciduous forest	graminoid	43.295	131.145
Pteridium	aquilinum	temperate broadleaf deciduous forest	pteridophyte	43.295	131.145
Quercus	mongolica	temperate broadleaf deciduous forest	tree	43.295	131.145
Rhamnus	sp	temperate broadleaf deciduous forest	low to high shrub	43.295	131.145
Rubia	sylvatica	temperate broadleaf deciduous forest	forb	43.295	131.145
Salix	gracilistyla	temperate broadleaf deciduous forest	tree	43.295	131.145
Salix	viminalis	temperate broadleaf deciduous forest	tree	43.295	131.145
Sorbaria	sorbifolia	temperate broadleaf deciduous forest	low to high shrub	43.295	131.145
Thalictrum	tuberiferum	temperate broadleaf deciduous forest	forb	43.295	131.145
Tilia	amurensis	temperate broadleaf deciduous forest	tree	43.295	131.145
Ulmus	davidiana var japonica	temperate broadleaf deciduous forest	tree	43.295	131.145

Viburnum	sargentii	temperate broadleaf deciduous forest	low to high shrub	43.295	131.145
Vicia	unijuga	temperate broadleaf deciduous forest	forb	43.295	131.145
Acer	mono	temperate broadleaf deciduous forest	tree	43.115	130.995
Acer	ginnala	temperate broadleaf deciduous forest	tree	43.115	130.995
Aconitum	volubile	temperate broadleaf deciduous forest	forb	43.115	130.995
Adenophora	tetraphylla	temperate broadleaf deciduous forest	forb	43.115	130.995
Aegopodium	alpestre	temperate broadleaf deciduous forest	forb	43.115	130.995
Angelica	amurensis	temperate broadleaf deciduous forest	forb	43.115	130.995
Arctium	lappa	temperate deciduous woodland	forb	43.115	130.995
Aster	scaber	temperate deciduous woodland	forb	43.115	130.995
Astilbe	chinensis	temperate deciduous woodland	forb	43.115	130.995
Betula	platyphylla	temperate deciduous woodland	tree	43.115	130.995
Brachybotrys	paridiformis	temperate deciduous woodland	forb	43.115	130.995

Carex	appendiculata	temperate deciduous woodland	graminoid	43.115	130.995
Carex	pediformis	temperate deciduous woodland	graminoid	43.115	130.995
Caulophyllum	robustum	temperate deciduous woodland	forb	43.115	130.995
Chloranthus	japonicus	temperate deciduous woodland	forb	43.115	130.995
Chrysosplenium	alternifolium	temperate deciduous woodland	forb	43.115	130.995
Corylus	heterophylla	temperate deciduous woodland	tree	43.115	130.995
Dioscorea	nipponica	temperate deciduous woodland	climber	43.115	130.995
Acanthopanax	senticosus	temperate deciduous woodland	low to high shrub	43.115	130.995
Equisetum	hyemale	temperate deciduous woodland	pteridophyte	43.115	130.995
Euonymus	alatus	temperate deciduous woodland	low to high shrub	43.115	130.995
Fraxinus	rhynchophylla	temperate deciduous woodland	tree	43.115	130.995
Fraxinus	mandshurica	temperate deciduous woodland	tree	43.115	130.995
Impatiens	furcillata	temperate deciduous woodland	forb	43.115	130.995

Lespedeza	bicolor	temperate deciduous woodland	low to high shrub	43.115	130.995
Lonicera	maackii	temperate deciduous woodland	low to high shrub	43.115	130.995
Lychnis	fulgens	temperate deciduous woodland	forb	43.115	130.995
Maackia	amurensis	temperate deciduous woodland	tree	43.115	130.995
Paeonia	japonica	temperate deciduous woodland	low to high shrub	43.115	130.995
Cacalia	hastata	temperate deciduous woodland	forb	43.115	130.995
Phellodendron	amurense	temperate deciduous woodland	tree	43.115	130.995
Phlomis	maximowiczii	temperate deciduous woodland	forb	43.115	130.995
Pinus	koraiensis	temperate deciduous woodland	tree	43.115	130.995
Polygonatum	oderatum	temperate deciduous woodland	forb	43.115	130.995
Pteridium	aquilinum	temperate deciduous woodland	pteridophyte	43.115	130.995
Quercus	mongolica	temperate deciduous woodland	tree	43.115	130.995
Rhamnus	sp	temperate deciduous woodland	low to high shrub	43.115	130.995

Rubia	sp	temperate deciduous woodland	forb	43.115	130.995
Sorbaria	sorbifolia	temperate deciduous woodland	low to high shrub	43.115	130.995
Thalictrum	tuberiferum	temperate deciduous woodland	forb	43.115	130.995
Tilia	mandshurica	temperate deciduous woodland	tree	43.115	130.995
Ulmus	davidiana var japonica	temperate deciduous woodland	tree	43.115	130.995
Viburnum	sargentii	temperate deciduous woodland	low to high shrub	43.115	130.995
Vicia	unijuga	temperate deciduous woodland	forb	43.115	130.995
Vitis	amurensis	temperate deciduous woodland	climber	43.115	130.995
Adenophora	tetraphylla	temperate deciduous woodland	forb	43.385	129.665
Aegopodium	alpestre	temperate deciduous woodland	forb	43.385	129.665
Artemisia	keiskeana	temperate deciduous woodland	forb	43.385	129.665
Aster	scaber	temperate deciduous woodland	forb	43.385	129.665
Astilbe	chinensis	temperate deciduous woodland	forb	43.385	129.665

Atractylodes	japonica	temperate deciduous woodland	forb	43.385	129.665
Betula	utilis	temperate deciduous woodland	tree	43.385	129.665
Bupleurum	longiradiatum	temperate deciduous woodland	forb	43.385	129.665
Carex	sp	temperate deciduous woodland	graminoid	43.385	129.665
Caulophyllum	robustum	temperate needleleaf forest	forb	43.385	129.665
Clematis	fusca var violaceae	temperate needleleaf forest	climber	43.385	129.665
Convallaria	majalis	temperate needleleaf forest	geophyte	43.385	129.665
Corylus	heterophylla	temperate needleleaf forest	tree	43.385	129.665
Dracocephalum	argunense	temperate needleleaf forest	forb	43.385	129.665
Euonymus	pauciflorus	temperate needleleaf forest	low to high shrub	43.385	129.665
Euphorbia	lucorum	temperate needleleaf forest	forb	43.385	129.665
Larix	olgensis	temperate needleleaf forest	tree	43.385	129.665
Lespedeza	bicolor	temperate needleleaf forest	low to high shrub	43.385	129.665

Paeonia	japonica	temperate needleleaf forest	low to high shrub	43.385	129.665
Peucedanum	terebinthaceum	temperate needleleaf forest	forb	43.385	129.665
Philadelphus	tenuifolius	temperate needleleaf forest	low to high shrub	43.385	129.665
Polygonatum	oderatum	temperate needleleaf forest	forb	43.385	129.665
Quercus	mongolica	temperate needleleaf forest	tree	43.385	129.665
Ranunculus	chinensis	temperate needleleaf forest	forb	43.385	129.665
Rhamnus	schneideri	temperate needleleaf forest	low to high shrub	43.385	129.665
Rhododendron	sp	temperate needleleaf forest	low to high shrub	43.385	129.665
Scutellaria	sp	temperate needleleaf forest	forb	43.385	129.665
Sedum	aizoon	temperate needleleaf forest	forb	43.385	129.665
Syneilesis	aconitifolia	temperate needleleaf forest	forb	43.385	129.665
Tilia	amurensis	temperate needleleaf forest	tree	43.385	129.665
Tilia	mandshurica	temperate needleleaf forest	tree	43.385	129.665

Trifolium	lupinaster	temperate needleleaf forest	forb	43.385	129.665
Vicia	unijuga	temperate needleleaf forest	forb	43.385	129.665
Viola	acuminata	temperate needleleaf forest	forb	43.385	129.665
Viola	sp 1	temperate needleleaf forest	forb	43.385	129.665
Viola	sp 2	temperate needleleaf forest	forb	43.385	129.665
Viola	sp 3	temperate needleleaf forest	forb	43.385	129.665
Acer	mono	temperate needleleaf forest	tree	43.245	128.645
Aconitum	kirinense	temperate needleleaf forest	forb	43.245	128.645
Arctium	lappa	temperate needleleaf forest	forb	43.245	128.645
Asparagus	sp	temperate needleleaf forest	ND	43.245	128.645
Aster	scaber	temperate needleleaf forest	forb	43.245	128.645
Astilbe	chinensis	temperate needleleaf forest	forb	43.245	128.645
Carex	siderosticta	temperate needleleaf forest	graminoid	43.245	128.645

Chloranthus	japonicus	temperate needleleaf forest	forb	43.245	128.645
Clintonia	udensis	temperate needleleaf forest	forb	43.245	128.645
Corylus	heterophylla	temperate needleleaf forest	tree	43.245	128.645
Euonymus	alatus	temperate needleleaf forest	low to high shrub	43.245	128.645
Fragaria	orientalis	temperate needleleaf forest	forb	43.245	128.645
Fraxinus	rhynchophylla	temperate shrubland	tree	43.245	128.645
Galium	manshuricum	temperate shrubland	forb	43.245	128.645
Geranium	sp	temperate shrubland	forb	43.245	128.645
Lespedeza	bicolor	temperate shrubland	low to high shrub	43.245	128.645
Lilium	lancifolium	temperate shrubland	geophyte	43.245	128.645
Monotropa	hypopitys	temperate shrubland	parasite	43.245	128.645
Cacalia	hastata	temperate shrubland	forb	43.245	128.645
Paris	verticillata	temperate shrubland	forb	43.245	128.645
Philadelphus	tenuifolius	temperate shrubland	low to high shrub	43.245	128.645
Pinus	koraiensis	temperate shrubland	tree	43.245	128.645
Pteridium	aquilinum	temperate shrubland	pteridophyte	43.245	128.645
Quercus	mongolica	temperate shrubland	tree	43.245	128.645
Rosa	sp	temperate shrubland	low to high shrub	43.245	128.645
Rubus	sp	temperate shrubland	erect dwarf shrub	43.245	128.645
Sambucus	sp	temperate shrubland	ND	43.245	128.645
Thalictrum	sp	temperate shrubland	forb	43.245	128.645

Tilia	mandshurica	temperate shrubland	tree	43.245	128.645
Ulmus	macrocarpa	temperate shrubland	tree	43.245	128.645
Unidentified forb 1		temperate shrubland	forb	43.245	128.645
Unidentified forb 2		temperate shrubland	forb	43.245	128.645
Unidentified forb 3		temperate shrubland	forb	43.245	128.645
Vicia	unijuga	temperate shrubland	forb	43.245	128.645
Viola	acuminata	temperate shrubland	forb	43.245	128.645
Viola	sp	temperate shrubland	forb	43.245	128.645
Vitis	amurensis	temperate shrubland	climber	43.245	128.645
Acer	mandschuricum	temperate shrubland	tree	43.735	127.035
Acer	mono	temperate shrubland	tree	43.735	127.035
Acer	tegmentosum	temperate shrubland	tree	43.735	127.035
Aconitum	jaluense var paniculigerum	temperate shrubland	climber	43.735	127.035
Actinidia	arguta	temperate shrubland	climber	43.735	127.035
Adiantum	pedatum	temperate shrubland	pteridophyte	43.735	127.035
Aegopodium	alpestre	temperate shrubland	forb	43.735	127.035
Aralia	elata	temperate shrubland	low to high shrub	43.735	127.035
Asparagus	sp	temperate shrubland	low to high shrub	43.735	127.035
Astilbe	chinensis	temperate shrubland	forb	43.735	127.035
Brachybotrys	paridiformis	temperate shrubland	forb	43.735	127.035
Cardamine	macrophylla	temperate shrubland	forb	43.735	127.035
Carex	meyeriana	temperate shrubland	graminoid	43.735	127.035
Carex	siderosticta	temperate shrubland	graminoid	43.735	127.035
Carex	sp	temperate broadleaf deciduous forest	graminoid	43.735	127.035

Carpinus	cordata	temperate broadleaf deciduous forest	tree	43.735	127.035
Clematis	sp	temperate broadleaf deciduous forest	climber	43.735	127.035
Corylus	heterophylla	temperate broadleaf deciduous forest	tree	43.735	127.035
Dioscorea	nipponica	temperate broadleaf deciduous forest	climber	43.735	127.035
Dryopteris	crassirhizoma	temperate broadleaf deciduous forest	pteridophyte	43.735	127.035
Acanthopanax	senticosus	temperate broadleaf deciduous forest	low to high shrub	43.735	127.035
Euonymus	alatus	temperate broadleaf deciduous forest	low to high shrub	43.735	127.035
Euonymus	pauciflorus	temperate broadleaf deciduous forest	low to high shrub	43.735	127.035
Fragaria	orientalis	temperate broadleaf deciduous forest	forb	43.735	127.035
Glycine	soja	temperate broadleaf deciduous forest	climber	43.735	127.035
Desmodium	fallax var mandshuricum	temperate broadleaf deciduous forest	forb	43.735	127.035
Impatiens	furcillata	temperate broadleaf deciduous forest	forb	43.735	127.035
Juglans	mandshurica	temperate broadleaf deciduous forest	tree	43.735	127.035

Lilium	lancifolium	temperate broadleaf deciduous forest	geophyte	43.735	127.035
Lonicera	maackii	temperate broadleaf deciduous forest	low to high shrub	43.735	127.035
Lonicera	monantha	temperate broadleaf deciduous forest	low to high shrub	43.735	127.035
Lychnis	fulgens	temperate broadleaf deciduous forest	forb	43.735	127.035
Matteuccia	struthiopteris	temperate broadleaf deciduous forest	pteridophyte	43.735	127.035
Cacalia	hastata	temperate broadleaf deciduous forest	forb	43.735	127.035
Philadelphus	tenuifolius	temperate broadleaf deciduous forest	low to high shrub	43.735	127.035
Phlomis	maximowiczii	temperate broadleaf deciduous forest	forb	43.735	127.035
Pinus	koraiensis	temperate broadleaf deciduous forest	tree	43.735	127.035
Populus	davidiana	temperate broadleaf deciduous forest	tree	43.735	127.035
Pteridium	aquilinum	temperate broadleaf deciduous forest	pteridophyte	43.735	127.035
Quercus	mongolica	temperate broadleaf deciduous forest	tree	43.735	127.035
Rubia	cordifolia	temperate broadleaf deciduous forest	climber	43.735	127.035

Rubia	sylvatica	temperate broadleaf deciduous forest	forb	43.735	127.035
Sanicula	chinenesis	temperate broadleaf deciduous forest	forb	43.735	127.035
Sedum	aizoon	temperate broadleaf deciduous forest	forb	43.735	127.035
Tilia	mandshurica	temperate broadleaf deciduous forest	tree	43.735	127.035
Ulmus	davidiana var japonica	temperate broadleaf deciduous forest	tree	43.735	127.035
Viburnum	burejaeticum	temperate broadleaf deciduous forest	low to high shrub	43.735	127.035
Viburnum	sargentii	temperate broadleaf deciduous forest	low to high shrub	43.735	127.035
Viola	acuminata	temperate broadleaf deciduous forest	forb	43.735	127.035
Viola	biflora	temperate broadleaf deciduous forest	forb	43.735	127.035
Allium	ramosum	temperate broadleaf deciduous forest	geophyte	43.805	125.685
Artemisia	sacrorum	temperate broadleaf deciduous forest	erect dwarf shrub	43.805	125.685
Artemisia	mongolica	temperate broadleaf deciduous forest	forb	43.805	125.685
Artemisia	scoparia	temperate broadleaf deciduous forest	forb	43.805	125.685

Artemisia	sieversiana	temperate broadleaf deciduous forest	forb	43.805	125.685
Cardamine	macrophylla	temperate broadleaf deciduous forest	forb	43.805	125.685
Carex	duriuscula	temperate broadleaf deciduous forest	graminoid	43.805	125.685
Clematis	hexapetala	temperate broadleaf deciduous forest	forb	43.805	125.685
Clematis	terniflora var mandshurica	temperate broadleaf deciduous forest	climber	43.805	125.685
Corylus	heterophylla	temperate broadleaf deciduous forest	tree	43.805	125.685
Crataegus	pinnatifida	temperate broadleaf deciduous forest	tree	43.805	125.685
Echinochloa	crusgalli	temperate broadleaf deciduous forest	graminoid	43.805	125.685
Euphorbia	mandshurica	temperate broadleaf deciduous forest	forb	43.805	125.685
Securinega	suffruticosa	temperate broadleaf deciduous forest	forb	43.805	125.685
Heteropappus	altaicus	temperate broadleaf deciduous forest	forb	43.805	125.685
Iris	lactea var chinensis	temperate broadleaf deciduous forest	geophyte	43.805	125.685
Kummerowia	striata	temperate broadleaf deciduous forest	forb	43.805	125.685

Campanula	falcata	temperate broadleaf deciduous forest	forb	43.805	125.685
Lespedeza	davurica	temperate broadleaf deciduous forest	forb	43.805	125.685
Linum	stelleroides	steppe	forb	43.805	125.685
Melilotoides	ruthenica	steppe	forb	43.805	125.685
Hieracium	denticuliferum	steppe	forb	43.805	125.685
Pinus	tabuliformis	steppe	tree	43.805	125.685
Platycodon	grandiflorus	steppe	forb	43.805	125.685
Polygala	tenuifolia	steppe	forb	43.805	125.685
Populus	davidiana	steppe	tree	43.805	125.685
Potentilla	chinensis	steppe	forb	43.805	125.685
Quercus	mongolica	steppe	tree	43.805	125.685
Rosa	sp	steppe	low to high shrub	43.805	125.685
Setaria	viridis	steppe	graminoid	43.805	125.685
Siphonostegia	chinensis	steppe	forb	43.805	125.685
Siphonostegia	chinensis	steppe	forb	43.805	125.685
Sonchus	brachyotus	steppe	forb	43.805	125.685
Tripolium	vulgare	steppe	forb	43.805	125.685
Ulmus	davidiana var japonica	steppe	tree	43.805	125.685
unidentified forb		steppe	forb	43.805	125.685
Viola	sp.	steppe	forb	43.805	125.685
Allium	senescens	steppe	geophyte	44.595	123.505
Artemisia	anethifolia	steppe	forb	44.595	123.505
Artemisia	scoparia	steppe	forb	44.595	123.505

Aster	ageratoides	steppe	forb	44.595	123.505
Calamagrostis	epigejos	steppe	graminoid	44.595	123.505
Chloris	virgata	steppe	graminoid	44.595	123.505
Echinochloa	crusgalli	steppe	graminoid	44.595	123.505
Inula	britannica	steppe	forb	44.595	123.505
Kalimeris	integrifolia	steppe	forb	44.595	123.505
Lamium	japonicum	steppe	forb	44.595	123.505
Leymus	chinensis	steppe	graminoid	44.595	123.505
Melilotus	suaveolens	steppe	forb	44.595	123.505
Phragmites	australis	steppe	graminoid	44.595	123.505
Phragmites	hirsutus	steppe	graminoid	44.595	123.505
Polygonum	sibiricum	steppe	forb	44.595	123.505
Puccinella	chinampoensis	steppe	graminoid	44.595	123.505
Sanguisorba	officinalis	steppe	forb	44.595	123.505
Thalictrum	simplex	steppe	forb	44.595	123.505
Amaranthus	retroflexus	steppe	forb	44.435	123.265
Artemisia	scoparia	steppe	forb	44.435	123.265
Astragalus	adsurgens	steppe	forb	44.435	123.265
Carex	duriuscula	steppe	graminoid	44.435	123.265
Chloris	virgata	steppe	graminoid	44.435	123.265
Ixeris	denticulata	steppe	forb	44.435	123.265
Echinochloa	crusgalli	steppe	graminoid	44.435	123.265
Eragrostis	minor	steppe	graminoid	44.435	123.265
Hieracium	denticulata	steppe	forb	44.435	123.265
Inula	britannica	steppe	forb	44.435	123.265

Kochia	scoparia var sieversiana	steppe	forb	44.435	123.265
Kummerowia	striata	steppe	forb	44.435	123.265
Leymus	chinensis	steppe	graminoid	44.435	123.265
Potentilla	chinensis	steppe	forb	44.435	123.265
Potentilla	flagellaris	steppe	forb	44.435	123.265
Taraxacum	ohwianum	steppe	forb	44.435	123.265
Allium	sp	steppe	geophyte	43.595	121.845
Atriplex	gmelinii	steppe	forb	43.595	121.845
Chenopodium	glaucum	steppe	forb	43.595	121.845
Chloris	virgata	steppe	graminoid	43.595	121.845
Erodium	stephanianum	steppe	forb	43.595	121.845
Iris	lactea var chinensis	steppe	geophyte	43.595	121.845
Kochia	scoparia var sieversiana	steppe	forb	43.595	121.845
Lepidium	apetalum	steppe	forb	43.595	121.845
Leymus	chinensis	steppe	graminoid	43.595	121.845
Limonium	bicolor	steppe	forb	43.595	121.845
Metaplexis	japonica	steppe	forb	43.595	121.845
Phragmites	australis	steppe	graminoid	43.595	121.845
Polygonum	sibiricum	steppe	forb	43.595	121.845
Salsola	collina	steppe	forb	43.595	121.845
Setaria	viridis	steppe	graminoid	43.595	121.845
Swainsona	salsula	steppe	forb	43.595	121.845
Suaeda	glauca	steppe	forb	43.595	121.845
Taraxacum	ohwianum	steppe	forb	43.595	121.845

Thermopsis	lanceolata	steppe	forb	43.595	121.845
Messerschmidia	sibirica	steppe	forb	43.595	121.845
Aristida	adscensionis	steppe	graminoid	44.125	121.765
Artemisia	scoparia	steppe	forb	44.125	121.765
Chenopodium	acuminatum	steppe	forb	44.125	121.765
Eragrostis	minor	steppe	graminoid	44.125	121.765
Setaria	viridis	steppe	graminoid	44.125	121.765
Tragus	racemosus	steppe	graminoid	44.125	121.765
Allium	mongolicum	steppe	geophyte	44.385	120.545
Artemisia	frigida	steppe	erect dwarf shrub	44.385	120.545
Artemisia	sacrorum	steppe	erect dwarf shrub	44.385	120.545
Artemisia	sacrorum var glauca	steppe	erect dwarf shrub	44.385	120.545
Cleistogenes	squarrosa	steppe	graminoid	44.385	120.545
Convolvulus	arvensis	steppe	climber	44.385	120.545
Hedysarum	fruticosum	steppe	forb	44.385	120.545
Heteropappus	altaicus	steppe	forb	44.385	120.545
Leontopodium	leontopodioides	steppe	forb	44.385	120.545
Lespedeza	davurica	steppe	forb	44.385	120.545
Lathyrus	hedysaroides var subsericea	steppe	forb	44.385	120.545
Lespedeza	hedysaroides	steppe	forb	44.385	120.545
Patrinia	rupestris	steppe	forb	44.385	120.545
Pennisetum	flaccidum	steppe	graminoid	44.385	120.545
Rhaponticum	uniflorum	steppe	forb	44.385	120.545
Salsola	collina	steppe	forb	44.385	120.545
Serratula	yamatsutana	steppe	forb	44.385	120.545

Setaria	viridis	steppe	graminoid	44.385	120.545
Stipa	krylovii	steppe	graminoid	44.385	120.545
Thalictrum	squarrosum	steppe	forb	44.385	120.545
Unidentified grass 1		steppe	graminoid	44.385	120.545
Aristida	adscensionis	steppe	graminoid	44.215	120.365
Caragana	microphylla	steppe	low to high shrub	44.215	120.365
Chenopodium	acuminatum	steppe	forb	44.215	120.365
Cleistogenes	squarrosa	steppe	graminoid	44.215	120.365
Cuscuta	chinensis	steppe	parasite	44.215	120.365
Chenopodium	aristatum	steppe	forb	44.215	120.365
Echinops	gmelinii	steppe	forb	44.215	120.365
Eragrostis	cilianensis	steppe	graminoid	44.215	120.365
Euphorbia	humifusa var pilosa	steppe	forb	44.215	120.365
Ferula	bungeana	steppe	forb	44.215	120.365
Medicago	lupulina	steppe	forb	44.215	120.365
Pennisetum	flaccidum	steppe	graminoid	44.215	120.365
Salix	gordejevii	steppe	low to high shrub	44.215	120.365
Salsola	ruthenica	steppe	forb	44.215	120.365
Setaria	viridis	steppe	graminoid	44.215	120.365
Thalictrum	squarrosum	steppe	forb	44.215	120.365
Tragus	racemosus	steppe	graminoid	44.215	120.365
Tribulus	terrestris	steppe	forb	44.215	120.365
Agropyron	michnoi	steppe	graminoid	43.885	119.385
Allium	ramosum	steppe	geophyte	43.885	119.385
Artemisia	annua	steppe	forb	43.885	119.385
Artemisia	mongolica	steppe	forb	43.885	119.385

Cleistogenes	squarrosa	steppe	graminoid	43.885	119.385
Ephedra	distachya	steppe	erect dwarf shrub	43.885	119.385
Hedysarum	fruticosum	steppe	forb	43.885	119.385
Lespedeza	davurica	steppe	forb	43.885	119.385
Leymus	chinensis	temperate grassland	graminoid	43.885	119.385
Melilotoides	ruthenica	temperate grassland	forb	43.885	119.385
Pennisetum	flaccidum	temperate grassland	graminoid	43.885	119.385
Polygonum	divaricatum	temperate grassland	forb	43.885	119.385
Potentilla	chinensis	temperate grassland	forb	43.885	119.385
Ulmus	davidiana var japonica	temperate grassland	tree	43.885	119.385
Allium	mongolicum	temperate grassland	geophyte	43.755	119.115
Arnebia	guttata	temperate grassland	forb	43.755	119.115
Artemisia	annua	temperate grassland	forb	43.755	119.115
Artemisia	frigida	temperate grassland	erect dwarf shrub	43.755	119.115
Atraphaxis	manshurica	temperate grassland	forb	43.755	119.115
Belamcanda	chinensis	temperate grassland	geophyte	43.755	119.115
Cleistogenes	squarrosa	temperate grassland	graminoid	43.755	119.115
Cynanchum	sibiricum	temperate grassland	forb	43.755	119.115
Delphinium	grandiflorum	temperate grassland	forb	43.755	119.115
Dianthus	chinensis	temperate grassland	forb	43.755	119.115
Geranium	transbaicalicum	temperate grassland	forb	43.755	119.115
Lespedeza	davurica	temperate grassland	forb	43.755	119.115
Leymus	chinensis	temperate grassland	graminoid	43.755	119.115
Melilotoides	ruthenica	temperate grassland	forb	43.755	119.115
Pennisetum	flaccidum	temperate grassland	graminoid	43.755	119.115

Polygonum	divaricatum	temperate grassland	forb	43.755	119.115
Potentilla	conferta	temperate grassland	forb	43.755	119.115
Salsola	collina	temperate grassland	forb	43.755	119.115
Siler	divaricatum	temperate grassland	forb	43.755	119.115
Scutellaria	baicalensis	temperate grassland	forb	43.755	119.115
Serratula	glauca	temperate grassland	forb	43.755	119.115
Sophora	flavescens	temperate grassland	forb	43.755	119.115
Thalictrum	squarrosum	temperate grassland	forb	43.755	119.115
Agropyron	michnoi	temperate grassland	graminoid	43.335	118.495
Bassia	dasyphylla	temperate grassland	forb	43.335	118.495
Caragana	microphylla	temperate grassland	low to high shrub	43.335	118.495
Chenopodium	acuminatum	temperate grassland	forb	43.335	118.495
Chloris	virgata	steppe	graminoid	43.335	118.495
Cynanchum	sibiricum	steppe	forb	43.335	118.495
Echinops	gmelinii	steppe	forb	43.335	118.495
Lespedeza	davurica	steppe	forb	43.335	118.495
Salsola	collina	steppe	forb	43.335	118.495
Scutellaria	viscidula	steppe	forb	43.335	118.495
Setaria	viridis	steppe	graminoid	43.335	118.495
Tribulus	terrestris	steppe	forb	43.335	118.495
Allium	neriniflorum	steppe	geophyte	43.195	117.765
Arnebia	guttata	steppe	forb	43.195	117.765
Artemisia	frigida	steppe	erect dwarf shrub	43.195	117.765
Artemisia	scoparia	steppe	forb	43.195	117.765
Cleistogenes	squarrosa	steppe	graminoid	43.195	117.765
Convolvulus	arvensis	steppe	climber	43.195	117.765

Dianthus	chinensis	steppe	forb	43.195	117.765
Dontostemon	integrifolius	steppe	forb	43.195	117.765
Chenopodium	aristatum	steppe	forb	43.195	117.765
Euphorbia	esula	steppe	forb	43.195	117.765
Geranium	transbaicalicum	steppe	forb	43.195	117.765
Heteropappus	altaicus	steppe	forb	43.195	117.765
Leonurus	japonicus	steppe	forb	43.195	117.765
Lespedeza	davurica	steppe	forb	43.195	117.765
Lespedeza	hedysaroides	steppe	forb	43.195	117.765
Leymus	chinensis	steppe	graminoid	43.195	117.765
Limonium	bicolor	steppe	forb	43.195	117.765
Melilotoides	ruthenica	steppe	forb	43.195	117.765
Pennisetum	flaccidum	steppe	graminoid	43.195	117.765
Polygonum	divaricatum	steppe	forb	43.195	117.765
Potentilla	chinensis	steppe	forb	43.195	117.765
Salsola	collina	steppe	forb	43.195	117.765
Agropyron	michnoi	steppe	graminoid	43.225	117.235
Allium	mongolicum	steppe	geophyte	43.225	117.235
Artemisia	frigida	steppe	erect dwarf shrub	43.225	117.235
Artemisia	scoparia	steppe	forb	43.225	117.235
Astragalus	adsurgens	steppe	forb	43.225	117.235
Carex	korshinskyi	temperate shrubland	graminoid	43.225	117.235
Cleistogenes	squarrosa	temperate shrubland	graminoid	43.225	117.235
Convolvulus	arvensis	temperate shrubland	climber	43.225	117.235
Dianthus	chinensis	temperate shrubland	forb	43.225	117.235
Dontostemon	integrifolius	temperate shrubland	forb	43.225	117.235

Filifolium	sibiricum	temperate shrubland	forb	43.225	117.235
Lespedeza	davurica	temperate shrubland	forb	43.225	117.235
Leymus	chinensis	temperate shrubland	graminoid	43.225	117.235
Melilotoides	ruthenica	temperate shrubland	forb	43.225	117.235
Polygonum	divaricatum	temperate shrubland	forb	43.225	117.235
Potentilla	acaulis	temperate shrubland	forb	43.225	117.235
Potentilla	bifurca	temperate shrubland	forb	43.225	117.235
Potentilla	chinensis	temperate shrubland	forb	43.225	117.235
Serratula	yamatsutana	temperate shrubland	forb	43.225	117.235
Stipa	grandis	temperate shrubland	graminoid	43.225	117.235
Thalictrum	squarrosum	temperate shrubland	forb	43.225	117.235
Ulmus	davidiana var japonica	temperate shrubland	tree	43.225	117.235
Unidentified grass 1		temperate shrubland	graminoid	43.225	117.235
Allium	ramosum	temperate shrubland	geophyte	43.385	116.885
Artemisia	frigida	temperate shrubland	erect dwarf shrub	43.385	116.885
Artemisia	scoparia	temperate shrubland	forb	43.385	116.885
Carex	duriuscula	temperate shrubland	graminoid	43.385	116.885
Cleistogenes	squarrosa	temperate shrubland	graminoid	43.385	116.885
Heteropappus	altaicus	steppe	forb	43.385	116.885
Iris	lactea var chinensis	steppe	geophyte	43.385	116.885
Leymus	chinensis	steppe	graminoid	43.385	116.885
Melilotoides	ruthenica	steppe	forb	43.385	116.885
Potentilla	acaulis	steppe	forb	43.385	116.885
Potentilla	bifurca	steppe	forb	43.385	116.885
Salsola	collina	steppe	forb	43.385	116.885

Stipa	krylovii	steppe	graminoid	43.385	116.885
Achnatherum	sibiricum	steppe	graminoid	43.555	116.675
Agropyron	michnoi	steppe	graminoid	43.555	116.675
Allium	condensatum	steppe	geophyte	43.555	116.675
Allium	mongolicum	steppe	geophyte	43.555	116.675
Allium	ramosum	steppe	geophyte	43.555	116.675
Artemisia	frigida	steppe	erect dwarf shrub	43.555	116.675
Bupleurum	scorzonerifolium	steppe	forb	43.555	116.675
Caragana	microphylla	steppe	low to high shrub	43.555	116.675
Chenopodium	album	steppe	forb	43.555	116.675
Cymbaria	dahurica	steppe	forb	43.555	116.675
Galium	verum	steppe	forb	43.555	116.675
Glycyrrhiza	uralensis	steppe	low to high shrub	43.555	116.675
Heteropappus	altaicus	steppe	forb	43.555	116.675
Kochia	prostrata	steppe	forb	43.555	116.675
Schizonepeta	multifida	steppe	forb	43.555	116.675
Orostachys	malacophylla	steppe	forb	43.555	116.675
Polygonum	divaricatum	steppe	forb	43.555	116.675
Serratula	yamatsutana	steppe	forb	43.555	116.675
Spiraea	trilobata	steppe	low to high shrub	43.555	116.675
Stellera	chamaejasme	steppe	forb	43.555	116.675
Stipa	krylovii	steppe	graminoid	43.555	116.675
Thermopsis	lanceolata	steppe	forb	43.555	116.675
Allium	mongolicum	steppe	geophyte	43.695	116.635
Allium	ramosum	steppe	geophyte	43.695	116.635
Artemisia	frigida	steppe	erect dwarf shrub	43.695	116.635

Caragana	microphylla	steppe	low to high shrub	43.695	116.635
Chenopodium	album	steppe	forb	43.695	116.635
Cirsium	setosum	steppe	forb	43.695	116.635
Cleistogenes	squarrosa	steppe	graminoid	43.695	116.635
Chenopodium	aristatum	steppe	forb	43.695	116.635
Echinops	gmelinii	steppe	forb	43.695	116.635
Heteropappus	altaicus	steppe	forb	43.695	116.635
Kochia	prostrata	steppe	forb	43.695	116.635
Leymus	chinensis	steppe	graminoid	43.695	116.635
Melilotoides	ruthenica	steppe	forb	43.695	116.635
Poa	angustifolia	steppe	graminoid	43.695	116.635
Potentilla	acaulis	steppe	forb	43.695	116.635
Potentilla	bifurca	steppe	forb	43.695	116.635
Potentilla	chinensis	steppe	forb	43.695	116.635
Psammochloa	villosa	steppe	graminoid	43.695	116.635
Stipa	krylovii	steppe	graminoid	43.695	116.635
Adenophora	gmelinii	steppe	forb	43.915	116.315
Adenophora	stenanthina var crispata	steppe	forb	43.915	116.315
Allium	mongolicum	steppe	geophyte	43.915	116.315
Allium	ramosum	steppe	geophyte	43.915	116.315
Artemisia	frigida	steppe	erect dwarf shrub	43.915	116.315
Astragalus	variabilis	steppe	forb	43.915	116.315
Bupleurum	scorzonerifolium	steppe	forb	43.915	116.315
Caragana	microphylla	steppe	low to high shrub	43.915	116.315
Chloris	virgata	steppe	graminoid	43.915	116.315

Gentiana	dahurica	temperate deciduous woodland	forb	43.915	116.315
Heteropappus	altaicus	temperate deciduous woodland	forb	43.915	116.315
Iris	lactea var chinensis	temperate deciduous woodland	geophyte	43.915	116.315
Leymus	chinensis	temperate deciduous woodland	graminoid	43.915	116.315
Melilotoides	ruthenica	temperate deciduous woodland	forb	43.915	116.315
Oxytropis	myriophylla	temperate deciduous woodland	forb	43.915	116.315
Oxytropis	sp	temperate deciduous woodland	forb	43.915	116.315
Phlomis	tuberosa	temperate deciduous woodland	forb	43.915	116.315
Potentilla	acaulis	temperate deciduous woodland	forb	43.915	116.315
Potentilla	chinensis	temperate deciduous woodland	forb	43.915	116.315
Rhaponticum	uniflorum	temperate deciduous woodland	forb	43.915	116.315
Serratula	yamatsutana	temperate deciduous woodland	forb	43.915	116.315
Stipa	krylovii	temperate deciduous woodland	graminoid	43.915	116.315

Thalictrum	squarrosum var supradecompositum	temperate deciduous woodland	forb	43.915	116.315
Achnatherum	sibiricum	temperate deciduous woodland	graminoid	43.895	115.325
Agropyron	michnoi	temperate deciduous woodland	graminoid	43.895	115.325
Allium	condensatum	temperate deciduous woodland	geophyte	43.895	115.325
Allium	mongolicum	temperate deciduous woodland	geophyte	43.895	115.325
Allium	ramosum	temperate deciduous woodland	geophyte	43.895	115.325
Artemisia	frigida	temperate deciduous woodland	erect dwarf shrub	43.895	115.325
Astragalus	adsurgens	steppe	forb	43.895	115.325
Astragalus	galactites	steppe	forb	43.895	115.325
Astragalus	scaberrimus	steppe	forb	43.895	115.325
Bupleurum	scorzonerifolium	steppe	forb	43.895	115.325
Caragana	stenophylla	steppe	low to high shrub	43.895	115.325
Ephedra	sinica	steppe	erect dwarf shrub	43.895	115.325
Glycyrrhiza	uralensis	steppe	low to high shrub	43.895	115.325
Haplophyllum	dauricum	steppe	forb	43.895	115.325
Heteropappus	altaicus	steppe	forb	43.895	115.325
Leymus	chinensis	steppe	graminoid	43.895	115.325
Limonium	bicolor	steppe	forb	43.895	115.325
Melilotoides	ruthenica	steppe	forb	43.895	115.325

Potentilla	chinensis	steppe	forb	43.895	115.325
Rhaponticum	uniflorum	steppe	forb	43.895	115.325
Siler	divaricatum	steppe	forb	43.895	115.325
Serratula	yamatsutana	steppe	forb	43.895	115.325
Setaria	viridis	steppe	graminoid	43.895	115.325
Stipa	krylovii	steppe	graminoid	43.895	115.325
Thalictrum	sp	steppe	forb	43.895	115.325
Thalictrum	squarrosum	steppe	forb	43.895	115.325
Allium	mongolicum	steppe	geophyte	43.935	114.615
Allium	polyrhizum	steppe	geophyte	43.935	114.615
Allium	ramosum	steppe	geophyte	43.935	114.615
Artemisia	frigida	steppe	erect dwarf shrub	43.935	114.615
Artemisia	pubescens	steppe	forb	43.935	114.615
Asparagus	dauricus	steppe	forb	43.935	114.615
Caragana	microphylla	steppe	low to high shrub	43.935	114.615
Caragana	stenophylla	steppe	low to high shrub	43.935	114.615
Chenopodium	acuminatum	steppe	forb	43.935	114.615
Convolvulus	ammannii	steppe	forb	43.935	114.615
Cymbaria	dahurica	steppe	forb	43.935	114.615
Heteropappus	altaicus	steppe	forb	43.935	114.615
Kochia	prostrata	steppe	forb	43.935	114.615
Melilotoides	ruthenica	steppe	forb	43.935	114.615
Rhaponticum	uniflorum	steppe	forb	43.935	114.615
Salsola	collina	steppe	forb	43.935	114.615
Stipa	krylovii	steppe	graminoid	43.935	114.615
unidentified chenopod 1		steppe	forb	43.935	114.615

Agropyron	michnoi	steppe	graminoid	43.835	113.825
Allium	mongolicum	steppe	geophyte	43.835	113.825
Allium	ramosum	steppe	geophyte	43.835	113.825
Artemisia	frigida	steppe	erect dwarf shrub	43.835	113.825
Asparagus	dauricus	steppe	forb	43.835	113.825
Caragana	microphylla	steppe	low to high shrub	43.835	113.825
Caragana	stenophylla	steppe	low to high shrub	43.835	113.825
Cleistogenes	songorica	steppe	graminoid	43.835	113.825
Convolvulus	ammannii	steppe	forb	43.835	113.825
Peganum	harmala	steppe	forb	43.835	113.825
Salsola	collina	steppe	forb	43.835	113.825
Scorzonera	divaricata	steppe	forb	43.835	113.825
Stipa	krylovii	steppe	graminoid	43.835	113.825
Allium	bidentatum	steppe	geophyte	43.805	113.355
Allium	leucocephalum	steppe	geophyte	43.805	113.355
Asparagus	dauricus	steppe	forb	43.805	113.355
Caragana	microphylla	steppe	low to high shrub	43.805	113.355
Caragana	stenophylla	steppe	low to high shrub	43.805	113.355
Festuca	dahurica	steppe	graminoid	43.805	113.355
Kochia	prostrata	steppe	forb	43.805	113.355
Salsola	collina	steppe	forb	43.805	113.355
Scorzonera	divaricata	steppe	forb	43.805	113.355
Stipa	glareosa	steppe	graminoid	43.805	113.355
Allium	ramosum	steppe	geophyte	43.725	112.585
Aristida	adscensionis	steppe	graminoid	43.725	112.585
Artemisia	frigida	steppe	erect dwarf shrub	43.725	112.585

Asparagus	dauricus	steppe	forb	43.725	112.585
Astragalus	sp	steppe	forb	43.725	112.585
Atraphaxis	bracteata	steppe	erect dwarf shrub	43.725	112.585
Caragana	microphylla	steppe	low to high shrub	43.725	112.585
Caragana	stenophylla	steppe	low to high shrub	43.725	112.585
Caragana	tibetica	steppe	erect dwarf shrub	43.725	112.585
Carex	duriuscula	steppe	graminoid	43.725	112.585
Cleistogenes	songorica	steppe	graminoid	43.725	112.585
Convolvulus	ammannii	steppe	forb	43.725	112.585
Eragrostis	minor	steppe	graminoid	43.725	112.585
Ferula	bungeana	steppe	forb	43.725	112.585
Haplophyllum	dauricum	steppe	forb	43.725	112.585
Hippolytia	trifida	steppe	erect dwarf shrub	43.725	112.585
Salsola	collina	steppe	forb	43.725	112.585
Scorzonera	divaricata	steppe	forb	43.725	112.585
Setaria	viridis var depressa	steppe	graminoid	43.725	112.585
Stipa	gobica	steppe	graminoid	43.725	112.585
Tribulus	terrestris	steppe	prostrate dwarf shrub	43.725	112.585
Agropyron	michnoi	steppe	graminoid	43.635	112.165
Allium	ramosum	steppe	geophyte	43.635	112.165
Asparagus	dauricus	steppe	forb	43.635	112.165
Caragana	microphylla	steppe	low to high shrub	43.635	112.165
Caragana	stenophylla	steppe	low to high shrub	43.635	112.165
Cleistogenes	songorica	steppe	graminoid	43.635	112.165
Convolvulus	ammannii	steppe	forb	43.635	112.165

Eragrostis	minor	steppe	graminoid	43.635	112.165
Euphorbia	humifusa	steppe	forb	43.635	112.165
Ferula	bungeana	steppe	forb	43.635	112.165
Haplophyllum	dauricum	steppe	forb	43.635	112.165
Hippolytia	trifida	steppe	erect dwarf shrub	43.635	112.165
Salsola	collina	steppe	forb	43.635	112.165
Scorzonera	divaricata	steppe	forb	43.635	112.165
Setaria	viridis var depressa	steppe	graminoid	43.635	112.165
Stipa	gobica	steppe	graminoid	43.635	112.165
Allium	ramosum	steppe	geophyte	43.665	111.915
Allium	tenuissimum	steppe	geophyte	43.665	111.915
Artemisia	sp	steppe	forb	43.665	111.915
Asparagus	dauricus	steppe	forb	43.665	111.915
Convolvulus	ammannii	steppe	forb	43.665	111.915
Haplophyllum	dauricum	steppe	forb	43.665	111.915
Haplophyllum	tragacanthoides	steppe	erect dwarf shrub	43.665	111.915
Hippolytia	trifida	steppe	erect dwarf shrub	43.665	111.915
Ceratoides	latens	steppe	erect dwarf shrub	43.665	111.915
Limonium	aureum	steppe	forb	43.665	111.915
Reaumuria	soongarica	steppe	low to high shrub	43.665	111.915
Salsola	passerina	steppe	erect dwarf shrub	43.665	111.915
Scorzonera	divaricata	steppe	forb	43.665	111.915
Scorzonera	muriculata	steppe	forb	43.665	111.915
Stipa	gobica	steppe	graminoid	43.665	111.915
Agropyron	michnoi	steppe	graminoid	43.655	111.885
Allium	ramosum	steppe	geophyte	43.655	111.885

Asparagus	dauricus	steppe	forb	43.655	111.885
Caragana	microphylla	steppe	low to high shrub	43.655	111.885
Caragana	stenophylla	steppe	low to high shrub	43.655	111.885
Cleistogenes	songorica	steppe	graminoid	43.655	111.885
Convolvulus	ammannii	steppe	forb	43.655	111.885
Ferula	bungeana	steppe	forb	43.655	111.885
Hippolytia	trifida	steppe	erect dwarf shrub	43.655	111.885
Iris	lactea var chinensis	steppe	geophyte	43.655	111.885
Ceratoides	latens	steppe	erect dwarf shrub	43.655	111.885
Peganum	harmala	steppe	forb	43.655	111.885
Salsola	collina	steppe	forb	43.655	111.885
Setaria	viridis	steppe	graminoid	43.655	111.885
Stipa	gobica	steppe	graminoid	43.655	111.885
Tribulus	terrestris	steppe	prostrate dwarf shrub	43.655	111.885
Broussonetia	papyrifera	steppe	tree	36.245	117.025
Grewia	biloba	steppe	low to high shrub	36.245	117.025
Pinus	tabuliformis	steppe	tree	36.245	117.025
Quercus	acutissima	steppe	tree	36.245	117.025
Quercus	fabrei	steppe	tree	36.245	117.025
Robinia	pseudoacacia	steppe	tree	36.245	117.025
Spiraea	trilobata	steppe	low to high shrub	36.245	117.025
Vitex	negundo var heterophylla	steppe	low to high shrub	36.245	117.025
Vitis	heyneana subsp ficifolia	steppe	liana	36.245	117.025

Albizia	kalkora	steppe	tree	34.635	119.235
Cerasus	japonica	steppe	tree	34.635	119.235
Clerodendrum	trichotomum	steppe	tree	34.635	119.235
Dalbergia	hupeana	steppe	tree	34.635	119.235
Glochidion	puberum	steppe	low to high shrub	34.635	119.235
Lespedeza	formosa	steppe	low to high shrub	34.635	119.235
Pinus	tabuliformis	steppe	tree	34.635	119.235
Platycladus	orientalis	steppe	tree	34.635	119.235
Platycodon	grandiflorus	steppe	liana	34.635	119.235
Quercus	acutissima	steppe	tree	34.635	119.235
Quercus	serrata var breviopetiolata	steppe	tree	34.635	119.235
Vitex	negundo	steppe	low to high shrub	34.635	119.235
Zanthoxylum	schinifolium	steppe	low to high shrub	34.635	119.235
Acer	buergerianum	steppe	tree	32.055	118.855
Alangium	chinense	steppe	low to high shrub	32.055	118.855
Aphananthe	aspera	steppe	tree	32.055	118.855
Celtis	sinensis	steppe	tree	32.055	118.855
Cercis	chinensis	steppe	tree	32.055	118.855
Cinnamomum	camphora	steppe	tree	32.055	118.855
Euonymus	alatus	steppe	low to high shrub	32.055	118.855
Firmiana	platanifolia	steppe	tree	32.055	118.855
Glochidion	puberum	steppe	low to high shrub	32.055	118.855
Podocarpium	podocarpum var oxyphyllum	steppe	low to high shrub	32.055	118.855
Ilex	cornuta	steppe	tree	32.055	118.855

Sabina	chinensis	steppe	tree	32.055	118.855
Kalopanax	septemlobus	temperate evergreen needleleaf forest	tree	32.055	118.855
Ligustrum	lucidum	temperate evergreen needleleaf forest	tree	32.055	118.855
Lindera	glauca	temperate evergreen needleleaf forest	low to high shrub	32.055	118.855
Liquidambar	formosana	temperate evergreen needleleaf forest	tree	32.055	118.855
Cudrania	tricuspidata	temperate evergreen needleleaf forest	low to high shrub	32.055	118.855
Osmanthus	fragrans	temperate evergreen needleleaf forest	low to high shrub	32.055	118.855
Paederia	scandens	temperate evergreen needleleaf forest	liana	32.055	118.855
Parthenocissus	tricuspidata	temperate evergreen needleleaf forest	liana	32.055	118.855
Phyllostachys	heteroclada	temperate evergreen needleleaf forest	bamboo	32.055	118.855
Pinus	massoniana	temperate evergreen needleleaf forest	tree	32.055	118.855
Pistacia	chinensis	temperate evergreen needleleaf forest	tree	32.055	118.855
Pittosporum	tobira	temperate evergreen needleleaf forest	low to high shrub	32.055	118.855

Quercus	acutissima	temperate evergreen needleleaf forest	tree	32.055	118.855
Quercus	aliena	temperate evergreen needleleaf forest	tree	32.055	118.855
Rosa	cymosa	temperate evergreen needleleaf forest	liana	32.055	118.855
Rubus	parvifolius	temperate evergreen needleleaf forest	liana	32.055	118.855
Serissa	serissoides	temperate evergreen needleleaf forest	erect dwarf shrub	32.055	118.855
Smilax	glaucochina	temperate evergreen needleleaf forest	liana	32.055	118.855
Symplocos	paniculata	temperate evergreen needleleaf forest	low to high shrub	32.055	118.855
Trachelospermum	jasminoides	temperate evergreen needleleaf forest	liana	32.055	118.855
Ulmus	parvifolia	temperate evergreen needleleaf forest	tree	32.055	118.855
Vernicia	fordii	temperate evergreen needleleaf forest	tree	32.055	118.855
Vitex	negundo	subtropical deciduous broadleaf forest	low to high shrub	32.055	118.855
Castanopsis	eyrei	subtropical deciduous broadleaf forest	tree	30.285	119.445
Castanopsis	sclerophylla	subtropical deciduous broadleaf forest	tree	30.285	119.445

Cunninghamia	lanceolata	subtropical deciduous broadleaf forest	tree	30.285	119.445
Cyclobalanopsis	glauca	subtropical deciduous broadleaf forest	tree	30.285	119.445
Dalbergia	hupeana	subtropical deciduous broadleaf forest	tree	30.285	119.445
Diospyros	lotus	subtropical deciduous broadleaf forest	tree	30.285	119.445
Eurya	rubiginosa var attenuata	subtropical deciduous broadleaf forest	low to high shrub	30.285	119.445
Gardenia	jasminoides	subtropical deciduous broadleaf forest	low to high shrub	30.285	119.445
Ilex	purpurea	subtropical deciduous broadleaf forest	tree	30.285	119.445
Lindera	aggregata	subtropical deciduous broadleaf forest	low to high shrub	30.285	119.445
Loropetalum	chinense	subtropical deciduous broadleaf forest	low to high shrub	30.285	119.445
Osmanthus	cooperi	subtropical deciduous broadleaf forest	tree	30.285	119.445
Photinia	glabra	subtropical deciduous broadleaf forest	tree	30.285	119.445
Raphiolepis	indica	subtropical deciduous broadleaf forest	low to high shrub	30.285	119.445
Rhododendron	mariesii	subtropical deciduous broadleaf forest	low to high shrub	30.285	119.445

Rhododendron	ovatum	subtropical deciduous broadleaf forest	low to high shrub	30.285	119.445
Schima	superba	subtropical deciduous broadleaf forest	tree	30.285	119.445
Smilax	china	subtropical deciduous broadleaf forest	liana	30.285	119.445
Symplocos	sumuntia	subtropical deciduous broadleaf forest	tree	30.285	119.445
Vaccinium	mandarinorum	subtropical deciduous broadleaf forest	low to high shrub	30.285	119.445
Wisteria	sinensis	subtropical deciduous broadleaf forest	liana	30.285	119.445
Ardisia	crenata var bicolor	subtropical deciduous broadleaf forest	low to high shrub	29.805	121.785
Ardisia	japonica	subtropical deciduous broadleaf forest	low to high shrub	29.805	121.785
Camellia	fraterna	subtropical deciduous broadleaf forest	low to high shrub	29.805	121.785
Camellia	oleifera	subtropical deciduous broadleaf forest	tree	29.805	121.785
Castanopsis	carlesii	subtropical deciduous broadleaf forest	tree	29.805	121.785
Castanopsis	fargesii	subtropical deciduous broadleaf forest	tree	29.805	121.785
Castanopsis	sclerophylla	subtropical deciduous broadleaf forest	tree	29.805	121.785

Celastrus	orbiculatus	subtropical deciduous broadleaf forest	liana	29.805	121.785
Cleyera	japonica	subtropical deciduous broadleaf forest	low to high shrub	29.805	121.785
Cyclobalanopsis	gilva	subtropical deciduous broadleaf forest	tree	29.805	121.785
Cyclobalanopsis	glauca	subtropical deciduous broadleaf forest	tree	29.805	121.785
Cyclobalanopsis	gracilis	subtropical deciduous broadleaf forest	small tree	29.805	121.785
Cyclobalanopsis	stewardiana	subtropical deciduous broadleaf forest	tree	29.805	121.785
Dalbergia	mimosoides	subtropical mixed forest	liana	29.805	121.785
Damnacanthus	indicus	subtropical mixed forest	low to high shrub	29.805	121.785
Dioscorea	cirrhosa	subtropical mixed forest	liana	29.805	121.785
Dioscorea	opposita	subtropical mixed forest	liana	29.805	121.785
Elaeocarpus	japonicus	subtropical mixed forest	tree	29.805	121.785
Eurya	rubiginosa	subtropical mixed forest	low to high shrub	29.805	121.785
Liquidambar	formosana	subtropical mixed forest	tree	29.805	121.785

Lithocarpus	glaber	subtropical mixed forest	tree	29.805	121.785
Loropetalum	chinense	subtropical mixed forest	low to high shrub	29.805	121.785
Machilus	thunbergii	subtropical mixed forest	tree	29.805	121.785
Morinda	umbellata	subtropical mixed forest	liana	29.805	121.785
Myrica	rubra	subtropical mixed forest	tree	29.805	121.785
Neolitsea	aurata var zhejinagensis	subtropical mixed forest	tree	29.805	121.785
Ormosia	henryi	subtropical mixed forest	tree	29.805	121.785
Pleioblastus	amarus	subtropical mixed forest	bamboo	29.805	121.785
Rhododendron	ovatum	subtropical mixed forest	low to high shrub	29.805	121.785
Schima	superba	subtropical mixed forest	tree	29.805	121.785
Smilax	china	subtropical mixed forest	liana	29.805	121.785
Stauntonia	chinensis	subtropical mixed forest	liana	29.805	121.785
Styrax	japonicus	subtropical mixed forest	low to high shrub	29.805	121.785

Symplocos	heishanensis	subtropical mixed forest	tree	29.805	121.785
Symplocos	stellaris	subtropical evergreen broadleaf forest	tree	29.805	121.785
Symplocos	sumuntia	subtropical evergreen broadleaf forest	tree	29.805	121.785
Symplocos	lancilimba	subtropical evergreen broadleaf forest	tree	29.805	121.785
Trachelospermum	jasminoides	subtropical evergreen broadleaf forest	liana	29.805	121.785
Tylophora	silvestris	subtropical evergreen broadleaf forest	liana	29.805	121.785
Vaccinium	mandarinorum	subtropical evergreen broadleaf forest	low to high shrub	29.805	121.785
Viburnum	erosum	subtropical evergreen broadleaf forest	low to high shrub	29.805	121.785
Acer	cordatum	subtropical evergreen broadleaf forest	tree	27.975	119.135
Adina	pilulifera	subtropical evergreen broadleaf forest	low to high shrub	27.975	119.135
Adinandra	megaphylla	subtropical evergreen broadleaf forest	tree	27.975	119.135
Ampelopsis	cantoniensis	subtropical evergreen broadleaf forest	liana	27.975	119.135
Antidesma	japonicum	subtropical evergreen broadleaf forest	low to high shrub	27.975	119.135

Millettia	reticulata	subtropical evergreen broadleaf forest	liana	27.975	119.135
Callicarpa	rubella	subtropical evergreen broadleaf forest	low to high shrub	27.975	119.135
Camellia	fraterna	subtropical evergreen broadleaf forest	low to high shrub	27.975	119.135
Camellia	sinensis	subtropical evergreen broadleaf forest	low to high shrub	27.975	119.135
Castanopsis	eyrei	subtropical evergreen broadleaf forest	tree	27.975	119.135
Castanopsis	fissa	subtropical evergreen broadleaf forest	tree	27.975	119.135
Castanopsis	sclerophylla	subtropical evergreen broadleaf forest	tree	27.975	119.135
Cinnamomum	camphora	subtropical evergreen broadleaf forest	tree	27.975	119.135
Coptosapelta	diffusa	subtropical evergreen broadleaf forest	liana	27.975	119.135
Cryptomeria	fortunei	subtropical evergreen broadleaf forest	tree	27.975	119.135
Cunninghamia	lanceolata	subtropical evergreen broadleaf forest	tree	27.975	119.135
Cyclobalanopsis	glauca	subtropical evergreen broadleaf forest	tree	27.975	119.135
Distylium	myricoides	subtropical evergreen broadleaf forest	low to high shrub	27.975	119.135

Elaeocarpus	japonicus	subtropical evergreen broadleaf forest	tree	27.975	119.135
Embelia	rudis	subtropical evergreen broadleaf forest	liana	27.975	119.135
Euscaphis	japonica	subtropical evergreen broadleaf forest	low to high shrub	27.975	119.135
Ficus	pandurata	subtropical evergreen broadleaf forest	low to high shrub	27.975	119.135
Ficus	pumila	subtropical evergreen broadleaf forest	liana	27.975	119.135
Ilex	pubescens	subtropical evergreen broadleaf forest	low to high shrub	27.975	119.135
Indigofera	ichangensis	subtropical evergreen broadleaf forest	low to high shrub	27.975	119.135
Itea	chinensis var oblonga	subtropical evergreen broadleaf forest	low to high shrub	27.975	119.135
Kadsura	longipedunculata	subtropical evergreen broadleaf forest	liana	27.975	119.135
Lasianthus	lancilimbus	subtropical evergreen broadleaf forest	low to high shrub	27.975	119.135
Laurocerasus	spinulosa	subtropical evergreen broadleaf forest	tree	27.975	119.135
Lindera	aggregata	subtropical evergreen broadleaf forest	low to high shrub	27.975	119.135
Lindera	glauca	subtropical evergreen broadleaf forest	low to high shrub	27.975	119.135

Liquidambar	formosana	subtropical evergreen broadleaf forest	tree	27.975	119.135
Lithocarpus	glaber	subtropical evergreen broadleaf forest	tree	27.975	119.135
Litsea	wilsonii	subtropical evergreen broadleaf forest	tree	27.975	119.135
Lonicera	cinerea	subtropical mixed forest	liana	27.975	119.135
Loropetalum	chinense	subtropical mixed forest	low to high shrub	27.975	119.135
Maesa	japonica	subtropical mixed forest	low to high shrub	27.975	119.135
Morinda	umbellata	subtropical mixed forest	liana	27.975	119.135
Oreocnide	frutescens	subtropical mixed forest	low to high shrub	27.975	119.135
Pericampylus	glaucus	subtropical mixed forest	liana	27.975	119.135
Pinus	massoniana	subtropical mixed forest	tree	27.975	119.135
Pittosporum	sahnianum	subtropical mixed forest	low to high shrub	27.975	119.135
Pleioblastus	amarus	subtropical mixed forest	bamboo	27.975	119.135
Premna	fordii	subtropical mixed forest	liana	27.975	119.135

Rosa	laevigata	subtropical mixed forest	liana	27.975	119.135
Rubus	corchorifolius	subtropical mixed forest	low to high shrub	27.975	119.135
Sabia	swinhoei	subtropical mixed forest	liana	27.975	119.135
Sageretia	thea	subtropical mixed forest	low to high shrub	27.975	119.135
Smilax	glabra	subtropical mixed forest	liana	27.975	119.135
Styrax	obassis	subtropical mixed forest	tree	27.975	119.135
Tarenna	mollissima	subtropical mixed forest	low to high shrub	27.975	119.135
Toxicodendron	succedaneum	subtropical mixed forest	tree	27.975	119.135
Trachelospermum	axillare	subtropical mixed forest	liana	27.975	119.135
Ulmus	changii	subtropical mixed forest	tree	27.975	119.135
Vaccinium	bracteatum	subtropical mixed forest	low to high shrub	27.975	119.135
Vaccinium	mandarinorum	subtropical mixed forest	low to high shrub	27.975	119.135
Vernicia	fordii	subtropical mixed forest	tree	27.975	119.135

Actinidia	eriantha	subtropical mixed forest	tree	26.585	118.055
Adina	pilulifera	subtropical mixed forest	low to high shrub	26.585	118.055
Randia	cochinchinensis	subtropical mixed forest	tree	26.585	118.055
Alyxia	vulgaris	subtropical mixed forest	liana	26.585	118.055
Ampelopsis	grossedentata	subtropical mixed forest	liana	26.585	118.055
Antidesma	japonicum	subtropical mixed forest	low to high shrub	26.585	118.055
Ardisia	lindleyana	subtropical mixed forest	liana	26.585	118.055
Camellia	cordifolia	subtropical mixed forest	low to high shrub	26.585	118.055
Camellia	fraterna	subtropical mixed forest	low to high shrub	26.585	118.055
Castanopsis	eyrei	subtropical mixed forest	tree	26.585	118.055
Castanopsis	fargesii	subtropical mixed forest	tree	26.585	118.055
Castanopsis	fissa	subtropical mixed forest	tree	26.585	118.055
Castanopsis	fordii	subtropical mixed forest	tree	26.585	118.055

Celastrus	hypoleucus	subtropical mixed forest	liana	26.585	118.055
Choerospondias	axillaris	subtropical mixed forest	tree	26.585	118.055
Coptosapelta	diffusa	subtropical mixed forest	liana	26.585	118.055
Cyclobalanopsis	glauca	subtropical mixed forest	tree	26.585	118.055
Daphniphyllum	oldhamii	subtropical mixed forest	tree	26.585	118.055
Diospyros	kaki var sylvestris	subtropical mixed forest	tree	26.585	118.055
Diploclisia	glaucescens	subtropical mixed forest	liana	26.585	118.055
Elaeocarpus	japonicus	subtropical mixed forest	tree	26.585	118.055
Embelia	rudis	subtropical mixed forest	liana	26.585	118.055
Eurya	nitida	subtropical mixed forest	low to high shrub	26.585	118.055
Ficus	fulva	subtropical mixed forest	low to high shrub	26.585	118.055
Fissistigma	oldhamii	subtropical mixed forest	liana	26.585	118.055
Gardenia	jasminoides	subtropical mixed forest	low to high shrub	26.585	118.055

Gnetum	parvifolium	subtropical mixed forest	liana	26.585	118.055
Helicia	cochinchinensis	subtropical mixed forest	tree	26.585	118.055
Ilex	pubescens	subtropical mixed forest	low to high shrub	26.585	118.055
Ilex	viridis	subtropical mixed forest	tree	26.585	118.055
Indocalamus	tessellatus	subtropical mixed forest	bamboo	26.585	118.055
Itea	chinensis var oblonga	subtropical mixed forest	low to high shrub	26.585	118.055
Lasianthus	lancilimbus	subtropical mixed forest	low to high shrub	26.585	118.055
Lithocarpus	glaber	subtropical mixed forest	tree	26.585	118.055
Litsea	wilsonii	subtropical mixed forest	tree	26.585	118.055
Loropetalum	chinense	subtropical mixed forest	low to high shrub	26.585	118.055
Lyonia	ovalifolia var elliptica	subtropical mixed forest	tree	26.585	118.055
Millettia	dielsiana	subtropical mixed forest	liana	26.585	118.055
Mussaenda	pubescens	subtropical mixed forest	low to high shrub	26.585	118.055

Paulownia	kawakamii	subtropical mixed forest	tree	26.585	118.055
Pericampylus	glaucus	subtropical mixed forest	liana	26.585	118.055
Phoebe	hunanensis	subtropical mixed forest	tree	26.585	118.055
Photinia	serrulata	subtropical mixed forest	tree	26.585	118.055
Photinia	parvifolia	subtropical mixed forest	tree	26.585	118.055
Pleioblastus	amarus	subtropical mixed forest	bamboo	26.585	118.055
Rubus	columellaris	subtropical mixed forest	low to high shrub	26.585	118.055
Rubus	corchorifolius	subtropical mixed forest	low to high shrub	26.585	118.055
Sarcandra	glabra	subtropical mixed forest	low to high shrub	26.585	118.055
Sloanea	sinensis	subtropical mixed forest	tree	26.585	118.055
Smilax	lanceifolia	subtropical mixed forest	liana	26.585	118.055
Styrax	calvescens	subtropical mixed forest	liana	26.585	118.055
Styrax	odoratissimus	subtropical mixed forest	tree	26.585	118.055

Symplocos	sumuntia	subtropical mixed forest	tree	26.585	118.055
Syzygium	austrosinense	subtropical mixed forest	tree	26.585	118.055
Tarenna	mollissima	subtropical mixed forest	low to high shrub	26.585	118.055
Tarennoidea	wallichii	subtropical mixed forest	tree	26.585	118.055
Toxicodendron	succedaneum	subtropical mixed forest	tree	26.585	118.055
Vaccinium	bracteatum	subtropical mixed forest	low to high shrub	26.585	118.055
Vernicia	montana	subtropical mixed forest	tree	26.585	118.055
Randia	cochinchinensis	subtropical mixed forest	tree	24.405	116.345
Alyxia	vulgaris	subtropical mixed forest	liana	24.405	116.345
Ardisia	lindleyana	subtropical mixed forest	liana	24.405	116.345
Castanopsis	fargesii	subtropical mixed forest	tree	24.405	116.345
Castanopsis	fissa	subtropical mixed forest	tree	24.405	116.345
Coptosapelta	diffusa	subtropical mixed forest	liana	24.405	116.345

Daphniphyllum	oldhamii	subtropical mixed forest	tree	24.405	116.345
Dendrotrophe	frutescens	subtropical mixed forest	liana	24.405	116.345
Diospyros	morrisiana	subtropical mixed forest	tree	24.405	116.345
Diospyros	tutcheri	subtropical mixed forest	tree	24.405	116.345
Elaeocarpus	glabripetalus	subtropical mixed forest	tree	24.405	116.345
Engelhardia	roxburghiana	subtropical mixed forest	tree	24.405	116.345
Evodia	fargesii	subtropical mixed forest	tree	24.405	116.345
Evodia	lepta	subtropical mixed forest	low to high shrub	24.405	116.345
Ficus	fulva	subtropical mixed forest	low to high shrub	24.405	116.345
Gardenia	jasminoides	subtropical mixed forest	low to high shrub	24.405	116.345
Glochidion	eriocarpum	subtropical mixed forest	low to high shrub	24.405	116.345
Ilex	asprella	subtropical mixed forest	low to high shrub	24.405	116.345
Ilex	pubescens	subtropical mixed forest	low to high shrub	24.405	116.345

Lithocarpus	glaber	subtropical mixed forest	tree	24.405	116.345
Litsea	cubeba	subtropical mixed forest	low to high shrub	24.405	116.345
Litsea	machiloides	subtropical mixed forest	low to high shrub	24.405	116.345
Litsea	wilsonii	subtropical mixed forest	tree	24.405	116.345
Mussaenda	erosa	subtropical mixed forest	liana	24.405	116.345
Photinia	glabra	subtropical mixed forest	tree	24.405	116.345
Raphiolepis	lanceolata	subtropical mixed forest	low to high shrub	24.405	116.345
Rhododendron	mariae	subtropical mixed forest	low to high shrub	24.405	116.345
Rhodomyrtus	tomentosa	subtropical mixed forest	low to high shrub	24.405	116.345
Schefflera	octophylla	subtropical mixed forest	tree	24.405	116.345
Schima	remotiserrata	subtropical mixed forest	tree	24.405	116.345
Smilax	china	subtropical mixed forest	liana	24.405	116.345
Syzygium	austrosinense	subtropical mixed forest	tree	24.405	116.345

Tarenna	attenuata	subtropical mixed forest	low to high shrub	24.405	116.345
Toxicodendron	succedaneum	subtropical mixed forest	tree	24.405	116.345
Sapium	discolor	tropical shrubland	tree	24.405	116.345
Acronychia	pedunculata	tropical shrubland	tree	23.175	112.535
Randia	cochinchinensis	tropical shrubland	tree	23.175	112.535
Aporosa	dioica	tropical shrubland	tree	23.175	112.535
Ardisia	punctata	tropical shrubland	low to high shrub	23.175	112.535
Ardisia	hanceana	tropical shrubland	low to high shrub	23.175	112.535
Ardisia	hypargyrea	tropical shrubland	low to high shrub	23.175	112.535
Blastus	cochinchinensis	tropical shrubland	low to high shrub	23.175	112.535
Calamus	hoplites	tropical shrubland	tree	23.175	112.535
Canarium	album	tropical shrubland	tree	23.175	112.535
Caryota	ochlandra	tropical shrubland	tree	23.175	112.535
Castanopsis	chinensis	tropical shrubland	tree	23.175	112.535
Castanopsis	fissa	tropical shrubland	tree	23.175	112.535
Cryptocarya	chinensis	tropical shrubland	tree	23.175	112.535
Cryptocarya	concinna	tropical shrubland	tree	23.175	112.535
Dasymaschalon	rostratum	tropical shrubland	tree	23.175	112.535
Diospyros	eriantha	tropical shrubland	tree	23.175	112.535
Dischidia	chinensis	tropical shrubland	epiphyte	23.175	112.535
Erycibe	obtusifolia	tropical shrubland	liana	23.175	112.535
Erythrophleum	fordii	tropical shrubland	tree	23.175	112.535
Ficus	wightiana	tropical shrubland	tree	23.175	112.535
Fissistigma	glaucescens	tropical shrubland	liana	23.175	112.535

Garcinia	oblongifolia	tropical shrubland	tree	23.175	112.535
Gironniera	subaequalis	tropical shrubland	tree	23.175	112.535
Gnetum	montanum	tropical shrubland	liana	23.175	112.535
Ixora	chinensis	tropical shrubland	low to high shrub	23.175	112.535
Lindera	chunii	tropical shrubland	tree	23.175	112.535
Machilus	chinensis	tropical shrubland	tree	23.175	112.535
Melastoma	sanguineum	tropical shrubland	low to high shrub	23.175	112.535
Meliosma	cuneifolia	tropical shrubland	tree	23.175	112.535
Memecylon	ligustrifolium	tropical shrubland	tree	23.175	112.535
Microdesmis	caseariifolia	tropical shrubland	low to high shrub	23.175	112.535
Ormosia	glaberrima	tropical shrubland	tree	23.175	112.535
Picrasma	chinensis	tropical shrubland	tree	23.175	112.535
Piper	chinense	tropical shrubland	liana	23.175	112.535
Psychotria	serpens	tropical grassland	liana	23.175	112.535
Rourea	minor	tropical grassland	liana	23.175	112.535
Sarcandra	glabra	tropical grassland	low to high shrub	23.175	112.535
Sarcosperma	arboreum	tropical grassland	tree	23.175	112.535
Schima	superba	tropical grassland	tree	23.175	112.535
Smilax	hypoglauca	tropical grassland	liana	23.175	112.535
Sterculia	lanceolata	tropical grassland	tree	23.175	112.535
Acmena	acuminatissima	tropical grassland	tree	23.175	112.535
Tetracera	asiatica	tropical grassland	liana	23.175	112.535
Tetrastigma	hemsleyanum	tropical grassland	liana	23.175	112.535
Tetrastigma	planicaule	tropical grassland	liana	23.175	112.535
Albizia	kalkora	tropical grassland	tree	25.315	110.255
Alchornea	trewioides	tropical grassland	low to high shrub	25.315	110.255

Bauhinia	championii	tropical grassland	liana	25.315	110.255
Millettia	reticulata	tropical grassland	liana	25.315	110.255
Celastrus	hindsii	tropical grassland	liana	25.315	110.255
Celtis	sinensis	tropical grassland	tree	25.315	110.255
Croton	tiglium	tropical grassland	low to high shrub	25.315	110.255
Cyclobalanopsis	glauca	tropical grassland	tree	25.315	110.255
Dalbergia	hupeana	tropical grassland	tree	25.315	110.255
Decaspermum	fruticosum	tropical grassland	low to high shrub	25.315	110.255
Ficus	variolosa	tropical grassland	low to high shrub	25.315	110.255
Firmiana	platanifolia	tropical grassland	tree	25.315	110.255
Fordia	cauliflora	tropical grassland	tree	25.315	110.255
Grewia	biloba	tropical grassland	low to high shrub	25.315	110.255
Ilex	hylonoma	tropical grassland	tree	25.315	110.255
Litsea	coreana var sinensis	tropical grassland	tree	25.315	110.255
Loropetalum	chinense	tropical grassland	low to high shrub	25.315	110.255
Cudrania	cochinchinensis	tropical grassland	low to high shrub	25.315	110.255
Millettia	dielsiana	tropical grassland	liana	25.315	110.255
Rapanea	kwangsiensis	tropical grassland	low to high shrub	25.315	110.255
Pinus	massoniana	tropical grassland	tree	25.315	110.255
Pueraria	lobata	tropical grassland	liana	25.315	110.255
Radermachera	sinica	tropical grassland	tree	25.315	110.255
Smilax	biumbellata	tropical grassland	liana	25.315	110.255
Styrax	odoratissimus	tropical grassland	tree	25.315	110.255
Trachelospermum	jasminoides	tropical grassland	liana	25.315	110.255
Sapium	rotundifolium	tropical grassland	tree	25.315	110.255
Vitex	negundo	tropical grassland	low to high shrub	25.315	110.255

Zanthoxylum	podocarpum	tropical grassland	low to high shrub	25.315	110.255
Acer	cinnamomifolium	tropical grassland	tree	26.845	109.605
Camellia	furfuracea	tropical grassland	tree	26.845	109.605
Camellia	oleifera	tropical grassland	tree	26.845	109.605
Carya	hunanensis	tropical grassland	tree	26.845	109.605
Celastrus	gemmatus	tropical grassland	liana	26.845	109.605
Celtis	sinensis	subtropical mixed forest	tree	26.845	109.605
Clematis	armandii	subtropical mixed forest	liana	26.845	109.605
Cyclobalanopsis	glauca	subtropical mixed forest	tree	26.845	109.605
Dalbergia	hancei	subtropical mixed forest	liana	26.845	109.605
Daphniphyllum	oldhamii	subtropical mixed forest	tree	26.845	109.605
Dichroa	febrifuga	subtropical mixed forest	low to high shrub	26.845	109.605
Diospyros	miaoshanica	subtropical mixed forest	low to high shrub	26.845	109.605
Euonymus	dielsianus	subtropical mixed forest	low to high shrub	26.845	109.605
Eurya	loquaiana	subtropical mixed forest	low to high shrub	26.845	109.605
Ficus	henryi	subtropical mixed forest	tree	26.845	109.605

Gardenia	jasminoides	subtropical mixed forest	low to high shrub	26.845	109.605
Hovenia	acerba	subtropical mixed forest	tree	26.845	109.605
Podocarpium	podocarpum var fallax	subtropical mixed forest	low to high shrub	26.845	109.605
Podocarpium	podocarpum var oxyphyllum	subtropical mixed forest	low to high shrub	26.845	109.605
Ilex	purpurea	subtropical mixed forest	tree	26.845	109.605
Kadsura	longipedunculata	subtropical mixed forest	liana	26.845	109.605
Laurocerasus	zippeliana	subtropical mixed forest	tree	26.845	109.605
Ligustrum	sinense	subtropical mixed forest	tree	26.845	109.605
Lindera	communis	subtropical mixed forest	tree	26.845	109.605
Lindera	megaphylla	subtropical mixed forest	tree	26.845	109.605
Lithocarpus	glaber	subtropical mixed forest	tree	26.845	109.605
Litsea	coreana	subtropical mixed forest	tree	26.845	109.605
Machilus	pauhoi	subtropical mixed forest	tree	26.845	109.605

Macropanax	rosthornii	subtropical mixed forest	tree	26.845	109.605
Maesa	japonica	subtropical mixed forest	low to high shrub	26.845	109.605
Maesa	perlaria	subtropical mixed forest	low to high shrub	26.845	109.605
Mallotus	philippensis	subtropical mixed forest	tree	26.845	109.605
Millettia	dielsiana	subtropical mixed forest	liana	26.845	109.605
Osmanthus	fragrans	subtropical mixed forest	low to high shrub	26.845	109.605
Parthenocissus	laetevirens	subtropical evergreen needleleaf forest	liana	26.845	109.605
Phoebe	sheareri	subtropical evergreen needleleaf forest	tree	26.845	109.605
Photinia	beauverdiana	subtropical evergreen needleleaf forest	tree	26.845	109.605
Piper	hancei	subtropical evergreen needleleaf forest	liana	26.845	109.605
Piper	wallichii	subtropical evergreen needleleaf forest	liana	26.845	109.605
Rubus	ichangensis	subtropical evergreen needleleaf forest	liana	26.845	109.605
Rubus	irenaeus	subtropical evergreen needleleaf forest	low to high shrub	26.845	109.605

Rubus	malifolius	subtropical evergreen needleleaf forest	liana	26.845	109.605
Sabia	swinhoei	subtropical evergreen needleleaf forest	liana	26.845	109.605
Sageretia	henryi	subtropical evergreen needleleaf forest	liana	26.845	109.605
Serissa	serissoides	subtropical evergreen needleleaf forest	erect dwarf shrub	26.845	109.605
Smilax	polycolea	subtropical evergreen needleleaf forest	low to high shrub	26.845	109.605
Symplocos	laurina	subtropical evergreen needleleaf forest	tree	26.845	109.605
Tetrastigma	wulinshanense	subtropical evergreen needleleaf forest	liana	26.845	109.605
Toxicodendron	sylvestre	subtropical evergreen needleleaf forest	tree	26.845	109.605
Trachelospermum	jasminoides	subtropical evergreen needleleaf forest	liana	26.845	109.605
Turpinia	arguta	subtropical evergreen needleleaf forest	low to high shrub	26.845	109.605
Viburnum	brachybotryum	subtropical evergreen needleleaf forest	tree	26.845	109.605
Viburnum	dilatatum	subtropical evergreen needleleaf forest	low to high shrub	26.845	109.605
Akebia	trifoliata	subtropical evergreen needleleaf forest	liana	28.345	109.725

Antidesma	japonicum	subtropical evergreen needleleaf forest	low to high shrub	28.345	109.725
Aralia	chinensis	subtropical evergreen needleleaf forest	low to high shrub	28.345	109.725
Camellia	oleifera	subtropical evergreen needleleaf forest	tree	28.345	109.725
Castanea	seguinii	subtropical evergreen needleleaf forest	tree	28.345	109.725
Castanopsis	fargesii	subtropical evergreen needleleaf forest	tree	28.345	109.725
Celtis	sinensis	subtropical evergreen needleleaf forest	tree	28.345	109.725
Cinnamomum	appelianum	subtropical evergreen needleleaf forest	tree	28.345	109.725
Clerodendrum	mandarinorum	subtropical evergreen needleleaf forest	tree	28.345	109.725
Swida	wilsoniana	subtropical evergreen needleleaf forest	tree	28.345	109.725
Cunninghamia	lanceolata	subtropical evergreen needleleaf forest	tree	28.345	109.725
Cyclobalanopsis	glauca	subtropical evergreen needleleaf forest	tree	28.345	109.725
Diospyros	cathayensis	subtropical evergreen needleleaf forest	tree	28.345	109.725
Diospyros	kaki var sylvestris	subtropical evergreen needleleaf forest	tree	28.345	109.725

Elaeagnus	henryi	subtropical evergreen needleleaf forest	liana	28.345	109.725
Acanthopanax	trifoliatus	subtropical evergreen needleleaf forest	low to high shrub	28.345	109.725
Eurya	alata	subtropical evergreen needleleaf forest	low to high shrub	28.345	109.725
Ficus	heteromorpha	subtropical evergreen needleleaf forest	tree	28.345	109.725
Ficus	pandurata var angustifolia	subtropical evergreen needleleaf forest	low to high shrub	28.345	109.725
Ficus	sarmentosa var henryi	subtropical evergreen needleleaf forest	liana	28.345	109.725
Podocarpium	podocarpum	subtropical evergreen needleleaf forest	low to high shrub	28.345	109.725
Jasminum	lanceolarium	subtropical evergreen needleleaf forest	liana	28.345	109.725
Kadsura	longipedunculata	subtropical evergreen needleleaf forest	liana	28.345	109.725
Kalopanax	septemlobus	subtropical evergreen needleleaf forest	tree	28.345	109.725
Litsea	coreana	subtropical evergreen needleleaf forest	tree	28.345	109.725
Loropetalum	chinense	subtropical evergreen needleleaf forest	low to high shrub	28.345	109.725
Maesa	japonica	subtropical evergreen needleleaf forest	low to high shrub	28.345	109.725

Mahonia	japonica	subtropical evergreen needleleaf forest	low to high shrub	28.345	109.725
Mussaenda	esquirolii	subtropical evergreen needleleaf forest	low to high shrub	28.345	109.725
Photinia	davidsoniae	subtropical evergreen needleleaf forest	tree	28.345	109.725
Pinus	massoniana	subtropical evergreen needleleaf forest	tree	28.345	109.725
Pistacia	chinensis	subtropical evergreen needleleaf forest	tree	28.345	109.725
Pittosporum	sahnianum	subtropical evergreen needleleaf forest	low to high shrub	28.345	109.725
Quercus	aliena	subtropical evergreen needleleaf forest	tree	28.345	109.725
Rhamnus	leptophylla	tropical shrubland	low to high shrub	28.345	109.725
Sageretia	henryi	tropical shrubland	liana	28.345	109.725
Serissa	serissoides	tropical shrubland	erect dwarf shrub	28.345	109.725
Toxicodendron	sylvestre	tropical shrubland	tree	28.345	109.725
Zanthoxylum	echinocarpum	tropical shrubland	liana	28.345	109.725
Acer	buergerianum	tropical shrubland	tree	33.505	111.485
Albizia	kalkora	tropical shrubland	tree	33.505	111.485
Artemisia	capillaris	tropical shrubland	low to high shrub	33.505	111.485
Asparagus	brachyphyllus	tropical shrubland	low to high shrub	33.505	111.485
Clematis	armandii	tropical shrubland	liana	33.505	111.485
Coriaria	nepalensis	tropical shrubland	tree	33.505	111.485
Cotinus	coggygria	tropical shrubland	tree	33.505	111.485

Dalbergia	hupeana	tropical shrubland	tree	33.505	111.485
Diospyros	lotus	tropical shrubland	tree	33.505	111.485
Lespedeza	bicolor	tropical shrubland	low to high shrub	33.505	111.485
Lonicera	tartarinowii	tropical shrubland	low to high shrub	33.505	111.485
Periploca	sepium	tropical shrubland	low to high shrub	33.505	111.485
Pinus	tabuliformis	tropical shrubland	tree	33.505	111.485
Pistacia	chinensis	tropical shrubland	tree	33.505	111.485
Platycladus	orientalis	tropical shrubland	tree	33.505	111.485
Pyrus	betulifolia	tropical shrubland	tree	33.505	111.485
Quercus	aliena	tropical shrubland	tree	33.505	111.485
Quercus	baronii	tropical shrubland	tree	33.505	111.485
Quercus	chenii	tropical shrubland	tree	33.505	111.485
Rhamnus	leptophylla	tropical shrubland	low to high shrub	33.505	111.485
Rhus	chinensis	tropical shrubland	tree	33.505	111.485
Sageretia	thea	tropical shrubland	low to high shrub	33.505	111.485
Smilax	hypoglauca	tropical shrubland	liana	33.505	111.485
Vernicia	fordii	tropical shrubland	tree	33.505	111.485
Vitex	negundo	tropical shrubland	low to high shrub	33.505	111.485
Vitis	bryoniifolia	tropical shrubland	liana	33.505	111.485
Zelkova	serrata	tropical shrubland	tree	33.505	111.485
Abelia	biflora	tropical shrubland	low to high shrub	39.955	115.425
Acer	mono	tropical shrubland	tree	39.955	115.425
Swida	bretschneideri	tropical shrubland	tree	39.955	115.425
Corylus	heterophylla	tropical shrubland	tree	39.955	115.425
Fraxinus	bungeana	tropical shrubland	tree	39.955	115.425
Juglans	mandshurica	tropical shrubland	tree	39.955	115.425

Lespedeza	bicolor	tropical shrubland	low to high shrub	39.955	115.425
Lonicera	maackii	cropland	low to high shrub	39.955	115.425
Quercus	mongolica	cropland	tree	39.955	115.425
Rhamnus	leptophylla	cropland	low to high shrub	39.955	115.425
Rubus	xanthocarpus	cropland	low to high shrub	39.955	115.425
Spiraea	pubescens	cropland	low to high shrub	39.955	115.425
Tilia	paucicostata	cropland	tree	39.955	115.425
Ulmus	davidiana var japonica	cropland	tree	39.955	115.425
Agriophyllum	arenarium	cropland	forb	48.195	87.025
Halogeton	sp.	cropland	erect dwarf shrub	48.195	87.025
Haloxylon	ammodendron	cropland	low to high shrub	48.195	87.025
Kalidium	foliatum	cropland	low to high shrub	48.195	87.025
Reaumuria	soongarica	cropland	low to high shrub	48.195	87.025
Salicornia	europaea	cropland	forb	48.195	87.025
Salsola	sp.	cropland	erect dwarf shrub	48.195	87.025
Suaeda	microphylla	cropland	low to high shrub	48.195	87.025
Amaranthus	sp.	cropland	forb	46.395	85.945
Artemisia	sp.	cropland	erect dwarf shrub	46.395	85.945
Atriplex	centralasiatica	cropland	erect dwarf shrub	46.395	85.945
Chenopodium	iljinii	cropland	forb	46.395	85.945
Chloris	sp.	cropland	graminoid	46.395	85.945
Corispermum	chinganicum	cropland	erect dwarf shrub	46.395	85.945
Cynoglossum	divaricatum	cropland	forb	46.395	85.945
Eragrostis	minor	cropland	graminoid	46.395	85.945
Halogeton	glomeratus	cropland	erect dwarf shrub	46.395	85.945

Halostachys	caspica	cropland	low to high shrub	46.395	85.945
Nitraria	tangutorum	cropland	low to high shrub	46.395	85.945
Salsola	collina	cropland	forb	46.395	85.945
Stipa	sp.	temperate shrubland	graminoid	46.395	85.945
Sympegma	regelii	temperate shrubland	erect dwarf shrub	46.395	85.945
Tribulus	terrestris	temperate shrubland	prostrate dwarf shrub	46.395	85.945
Zygophyllum	fabago	temperate shrubland	erect dwarf shrub	46.395	85.945
Ajania	fruticulosa	temperate shrubland	erect dwarf shrub	47.035	87.095
Alhagi	sparsifolia	temperate shrubland	low to high shrub	47.035	87.095
Allium	polyrhizum	temperate shrubland	geophyte	47.035	87.095
Anabasis	salsa	temperate shrubland	succulent	47.035	87.095
Astragalus	sp.	temperate shrubland	prostrate dwarf shrub	47.035	87.095
Ceratocarpus	arenarius	temperate shrubland	erect dwarf shrub	47.035	87.095
Cleistogenes	squarrosa	temperate shrubland	graminoid	47.035	87.095
Ceratoides	latens	temperate shrubland	erect dwarf shrub	47.035	87.095
Salsola	collina	temperate shrubland	forb	47.035	87.095
Serratula	marginata	temperate shrubland	forb	47.035	87.095
Allium	chrysanthum	steppe	geophyte	47.835	86.845
Artemisia	desertorum	steppe	forb	47.835	86.845
Atraphaxis	frutescens	steppe	low to high shrub	47.835	86.845
Bassia	dasyphylla	steppe	forb	47.835	86.845
Ceratocarpus	arenarius	steppe	erect dwarf shrub	47.835	86.845
Kochia	prostrata	steppe	forb	47.835	86.845
Ceratoides	latens	steppe	erect dwarf shrub	47.835	86.845

Limonium	sp.	steppe	forb	47.835	86.845
Limonium	sp.1	desert	forb	47.835	86.845
Limonium	sp.2	desert	forb	47.835	86.845
Nanophyton	erinaceum	desert	erect dwarf shrub	47.835	86.845
Pyrethrum	sp.	desert	forb	47.835	86.845
Salsola	collina	desert	forb	47.835	86.845
Setaria	viridis	desert	graminoid	47.835	86.845
Stipa	sp.	desert	graminoid	47.835	86.845
Suaeda	salsa	desert	forb	47.835	86.845
unidentified chenopod		desert	erect dwarf shrub	47.835	86.845
Xanthium	strumarium	desert	forb	47.835	86.845
Agriophyllum	arenarium	desert	forb	47.935	86.835
Aristida	adscensionis	desert	graminoid	47.935	86.835
Asterothamnus	centrali-asiaticus	desert	low to high shrub	47.935	86.835
Calligonum	rubicundum	desert	low to high shrub	47.935	86.835
Ephedra	intermedia	desert	low to high shrub	47.935	86.835
Ceratoides	latens	desert	erect dwarf shrub	47.935	86.835
Poaceae	sp	desert	graminoid	47.935	86.835
Poaceae	sp	desert	graminoid	47.935	86.835
Salsola	sp.	desert	low to high shrub	47.935	86.835
Saussurea	epilobioides	desert	forb	47.935	86.835
Sonchus	oleraceus	desert	forb	47.935	86.835
Artemisia	scoparia	desert	forb	48.165	87.075
Artemisia	sp.	desert	low to high shrub	48.165	87.075
Asteraceae	sp.	desert	forb	48.165	87.075
Astragalus	sp.	desert	ND	48.165	87.075

Carex	sp.	desert	graminoid	48.165	87.075
Carlina	biebersteinii	desert	forb	48.165	87.075
Chenopodiaceae	sp.	desert	low to high shrub	48.165	87.075
Chenopodium	sp.	desert	forb	48.165	87.075
Chenopodium	foetidum	desert	forb	48.165	87.075
Dianthus	chinensis var versicolor	desert	forb	48.165	87.075
Dracocephalum	sp.	desert	erect dwarf shrub	48.165	87.075
Geranium	sp	desert	forb	48.165	87.075
Polygonum	aviculare	desert	forb	48.165	87.075
Serratula	marginata	desert	forb	48.165	87.075
Achillea	millefolium	desert	forb	48.115	87.015
Polygonum	aviculare	desert	forb	48.115	87.015
Spiraea	media	desert	low to high shrub	48.115	87.015
Achillea	millefolium	desert	forb	48.335	87.125
Cyperus	sp.	desert	graminoid	48.335	87.125
Erodium	oxyrrhynchum	desert	forb	48.335	87.125
Fragaria	pentaphylla	desert	forb	48.335	87.125
Meconopsis	sp.	desert	forb	48.335	87.125
Mentha	sp1	desert	forb	48.335	87.125
Mentha	sp2	desert	forb	48.335	87.125
Nepeta	cataria	desert	forb	48.335	87.125
Polygonum	sp.	desert	forb	48.335	87.125
Potentilla	sp.	desert	forb	48.335	87.125
Stachys	sp.	temperate grassland	forb	48.335	87.125
Thymus	mongolicus	temperate grassland	erect dwarf shrub	48.335	87.125

Achnatherum	splendens	temperate grassland	graminoid	47.715	87.015
Anabasis	salsa	temperate grassland	succulent	47.715	87.015
Artemisia	kanashiroi	temperate grassland	forb	47.715	87.015
Atraphaxis	frutescens	temperate grassland	low to high shrub	47.715	87.015
Atriplex	centralasiatica	temperate grassland	erect dwarf shrub	47.715	87.015
Caragana	microphylla	temperate grassland	low to high shrub	47.715	87.015
Ceratocarpus	arenarius	temperate grassland	erect dwarf shrub	47.715	87.015
Cynanchum	chinense	temperate grassland	climber	47.715	87.015
Halogeton	sp.	temperate grassland	succulent	47.715	87.015
Kochia	prostrata	cropland	forb	47.715	87.015
Ceratoides	latens	cropland	erect dwarf shrub	47.715	87.015
Myosotis	alpestris subsp. asiatica	cropland	forb	47.715	87.015
Nanophyton	erinaceum	cropland	erect dwarf shrub	47.715	87.015
Nitraria	sibirica	cropland	low to high shrub	47.715	87.015
Plantago	lanceolata	cropland	forb	47.715	87.015
Polycnemum	arvense	cropland	forb	47.715	87.015
Reaumuria	soongarica	cropland	low to high shrub	47.715	87.015
Senecio	sp.	cropland	forb	47.715	87.015
Silene	sp.	cropland	forb	47.715	87.015
Sophora	alopecuroides	cropland	forb	47.715	87.015
Swainsona	salsula	cropland	low to high shrub	47.715	87.015
Stipa	sp.	cropland	graminoid	47.715	87.015
Anabasis	salsa	cropland	succulent	47.745	87.545
Artemisia	sp.	cropland	forb	47.745	87.545
Ceratocarpus	arenarius	steppe	erect dwarf shrub	47.745	87.545

Eragrostis	minor	steppe	graminoid	47.745	87.545
Ceratoides	latens	steppe	erect dwarf shrub	47.745	87.545
Meconopsis	sp.	steppe	forb	47.745	87.545
Peganum	harmala	steppe	forb	47.745	87.545
Polycnemum	arvense	steppe	forb	47.745	87.545
Polygonum	sp	temperate grassland	forb	47.745	87.545
Reaumuria	soongarica	temperate grassland	low to high shrub	47.745	87.545
Stipa	sp.	temperate grassland	graminoid	47.745	87.545
Tribulus	terrestris	temperate grassland	prostrate dwarf shrub	47.745	87.545
Zygophyllum	fabago	temperate grassland	low to high shrub	47.745	87.545
Artemisia	scoparia	temperate grassland	forb	47.155	88.705
Ceratocarpus	arenarius	temperate grassland	erect dwarf shrub	47.155	88.705
Halogeton	glomeratus	temperate grassland	erect dwarf shrub	47.155	88.705
Kochia	prostrata	temperate grassland	forb	47.155	88.705
Nanophyton	erinaceum	temperate grassland	erect dwarf shrub	47.155	88.705
Salsola	collina	temperate grassland	forb	47.155	88.705
Suaeda	prostrata	temperate grassland	forb	47.155	88.705
Anabasis	truncata	temperate grassland	forb	46.305	89.545
Artemisia	frigida	temperate grassland	erect dwarf shrub	46.305	89.545
Astragalus	sp.	temperate grassland	forb	46.305	89.545
Ceratocarpus	arenarius	temperate grassland	erect dwarf shrub	46.305	89.545
Kalidium	foliatum	temperate grassland	low to high shrub	46.305	89.545
Ceratoides	latens	temperate grassland	erect dwarf shrub	46.305	89.545
Limonium	sp.	temperate grassland	forb	46.305	89.545
Salicornia	europaea	temperate grassland	forb	46.305	89.545

Stipa	sp.	temperate grassland	graminoid	46.305	89.545
Allium	chrysanthum	temperate grassland	geophyte	45.355	89.405
Anabasis	aphylla	temperate grassland	erect dwarf shrub	45.355	89.405
Kalidium	foliatum	temperate grassland	low to high shrub	45.355	89.405
Reaumuria	soongarica	temperate grassland	low to high shrub	45.355	89.405
Stipa	sp.	temperate grassland	graminoid	45.355	89.405
Amaranthus	retroflexus	temperate grassland	forb	44.125	87.805
Ceratocarpus	arenarius	temperate grassland	erect dwarf shrub	44.125	87.805
Eragrostis	minor	temperate grassland	graminoid	44.125	87.805
Haloxylon	ammodendron	temperate grassland	low to high shrub	44.125	87.805
Kalidium	foliatum	temperate grassland	low to high shrub	44.125	87.805
Nitraria	tangutorum	temperate grassland	low to high shrub	44.125	87.805
Peganum	harmala	temperate grassland	forb	44.125	87.805
Reaumuria	soongarica	temperate grassland	low to high shrub	44.125	87.805
Salsola	collina	temperate grassland	forb	44.125	87.805
Suaeda	physophora	temperate grassland	low to high shrub	44.125	87.805
Amaranthus	sp.	temperate grassland	forb	44.075	87.795
Anabasis	aphylla	desert	erect dwarf shrub	44.075	87.795
Artemisia	sp	desert	ND	44.075	87.795
Atriplex	centralasiatica	desert	erect dwarf shrub	44.075	87.795
Ceratocarpus	arenarius	desert	erect dwarf shrub	44.075	87.795
Eragrostis	minor	desert	graminoid	44.075	87.795
Halogeton	sp.	desert	forb	44.075	87.795
Kalidium	foliatum	desert	low to high shrub	44.075	87.795
Ceratoides	latens	desert	erect dwarf shrub	44.075	87.795
Peganum	harmala	desert	forb	44.075	87.795

Reaumuria	soongarica	desert	low to high shrub	44.075	87.795
Salsola	collina	desert	forb	44.075	87.795
Salsola	collina	desert	forb	44.075	87.795
Solanaceae	sp.	desert	forb	44.075	87.795
Stipa	sp.	desert	graminoid	44.075	87.795
Suaeda	sp.	desert	forb	44.075	87.795
Artemisia	sp	desert	ND	44.065	88.075
Crepis	flexuosa	desert	forb	44.065	88.075
Atriplex	centralasiatica	desert	erect dwarf shrub	44.065	88.075
Caragana	microphylla	desert	low to high shrub	44.065	88.075
Ceratocarpus	arenarius	desert	erect dwarf shrub	44.065	88.075
Kochia	prostrata	desert	erect dwarf shrub	44.065	88.075
Tribulus	terrestris	desert	forb	44.065	88.075
Urtica	cannabina	desert	forb	44.065	88.075
Achnatherum	splendens	desert	graminoid	43.995	88.065
Amaranthus	sp.	desert	forb	43.995	88.065
Artemisia	sp	desert	ND	43.995	88.065
Caragana	microphylla	desert	low to high shrub	43.995	88.065
Carex	sp.	desert	graminoid	43.995	88.065
Ceratocarpus	arenarius	desert	erect dwarf shrub	43.995	88.065
Euphorbia	sp.	desert	forb	43.995	88.065
Medicago	sativa	desert	forb	43.995	88.065
Portulaca	oleracea	desert	forb	43.995	88.065
Potentilla	bifurca	desert	forb	43.995	88.065
Stipa	sp.	desert	graminoid	43.995	88.065
Berberis	amurensis	desert	low to high shrub	43.925	88.115

Chenopodium	foetidum	desert	forb	43.925	88.115
Corydalis	pallida	desert	forb	43.925	88.115
Cotoneaster	multiflorus	desert	low to high shrub	43.925	88.115
Populus	euphratica	desert	tree	43.925	88.115
Rosa	sp.	desert	low to high shrub	43.925	88.115
Rumex	sp.	desert	forb	43.925	88.115
Spiraea	mongolica	desert	low to high shrub	43.925	88.115
Ulmus	pumila	desert	tree	43.925	88.115
Berberis	sp.	desert	low to high shrub	43.925	88.115
Cotoneaster	multiflorus	desert	low to high shrub	43.925	88.115
Rosa	sp.	desert	low to high shrub	43.925	88.115
Alhagi	sparsifolia	desert	low to high shrub	42.835	89.435
Karelinia	caspia	desert	forb	42.835	89.435
Lycium	ruthenicum	desert	low to high shrub	42.725	89.435
Phragmites	australis	desert	graminoid	42.725	89.435
Halostachys	caspica	desert	low to high shrub	42.685	89.425
Tamarix	hispida	desert	low to high shrub	42.685	89.425
Ephedra	glauca	desert	low to high shrub	42.365	88.565
Halogeton	glomeratus	desert	erect dwarf shrub	42.365	88.565
Reaumuria	soongarica	desert	low to high shrub	42.365	88.565
Zygophyllum	kaschgaricum	desert	forb	42.365	88.565
Zygophyllum	xanthoxylum	desert	erect dwarf shrub	42.365	88.565
Artemisia	sp.	desert	ND	42.215	87.755
Brassicaceae	sp.	desert	forb	42.215	87.755
Ephedra	glauca	desert	low to high shrub	42.215	87.755
Haloxylon	ammodendron	desert	low to high shrub	42.215	87.755

Nitraria	tangutorum	desert	low to high shrub	42.215	87.755
Reaumuria	soongarica	desert	low to high shrub	42.215	87.755
Suaeda	heterophylla	desert	forb	42.215	87.755
Sympegma	regelii	desert	low to high shrub	42.215	87.755
unidentified forb		desert	forb	42.215	87.755
Reaumuria	soongarica	temperate deciduous woodland	low to high shrub	41.805	86.245
Sympegma	regelii	temperate deciduous woodland	low to high shrub	41.805	86.245
Halogeton	glomeratus	temperate deciduous woodland	erect dwarf shrub	40.825	84.295
Myricaria	sp.	temperate deciduous woodland	low to high shrub	40.825	84.295
Populus	euphratica	temperate deciduous woodland	tree	40.825	84.295
Salsola	collina	temperate deciduous woodland	forb	40.825	84.295
Reaumuria	soongarica	temperate deciduous woodland	low to high shrub	41.485	84.215
Tamarix	hispida	temperate deciduous woodland	low to high shrub	41.485	84.215
Karelinia	caspia	temperate deciduous woodland	forb	41.495	84.505
Phragmites	australis	temperate deciduous woodland	graminoid	41.495	84.505
Halogeton	glomeratus	desert	erect dwarf shrub	41.655	84.885

Halostachys	caspica	desert	low to high shrub	41.655	84.885
Kalidium	foliatum	desert	low to high shrub	41.655	84.885
Myricaria	sp.	desert	low to high shrub	41.655	84.885
Reaumuria	soongarica	desert	low to high shrub	41.655	84.885
Anemone	sp.	desert	forb	43.905	88.125
Caltha	palustris	desert	forb	43.905	88.125
Picea	schrenkiana	desert	tree	43.905	88.125
Polygonum	sp.	desert	forb	43.905	88.125
Stellaria	soongorica	desert	forb	43.905	88.125
Taraxacum	sp.	desert	forb	43.905	88.125
Trifolium	sp.	desert	forb	43.905	88.125
unknown	sp	steppe	ND	43.905	88.125
Viola	sp.	steppe	forb	43.905	88.125
Ammopiptanthus	mongolicus	steppe	low to high shrub	40.505	89.105
Zygophyllum	fabago	steppe	low to high shrub	40.505	89.105
Populus	euphratica	steppe	tree	40.825	84.295
Acacia	pennata	steppe	liana	21.915	101.275
Aesculus	lantsangensis	steppe	tree	21.915	101.275
Ailanthus	fordii	steppe	tree	21.915	101.275
Albizia	lucida	steppe	tree	21.915	101.275
Alstonia	scholaris	steppe	tree	21.915	101.275
Amischotolype	hispida	steppe	forb	21.915	101.275
Antiaris	toxicaria	steppe	tree	21.915	101.275
Ardisia	virens	steppe	low to high shrub	21.915	101.275
Baccaurea	ramiflora	steppe	tree	21.915	101.275
Barringtonia	macrostachya	temperate grassland	tree	21.915	101.275

Bolbitis	heteroclita	temperate grassland	pteridophyte	21.915	101.275
Canarium	album	temperate grassland	tree	21.915	101.275
Castanopsis	indica	temperate grassland	tree	21.915	101.275
Elatostema	rupestre	desert	forb	21.915	101.275
Embelia	vestita	desert	liana	21.915	101.275
Ficus	auriculata	desert	tree	21.915	101.275
Ficus	cyrtophylla	desert	tree	21.915	101.275
Ficus	subulata	desert	liana	21.915	101.275
Garcinia	cowa	desert	tree	21.915	101.275
Garuga	floribunda var gamblai	desert	tree	21.915	101.275
Justicia	patentiflora	desert	forb	21.915	101.275
Knema	cinerea var glauca	desert	tree	21.915	101.275
Laurocerasus	zippeliana	desert	tree	21.915	101.275
Leea	compactiflora	desert	low to high shrub	21.915	101.275
Lepisanthes	senegalensis	desert	low to high shrub	21.915	101.275
Macropanax	decandrus	desert	tree	21.915	101.275
Magnolia	henryi	desert	tree	21.915	101.275
Millettia	leptobotrya	temperate grassland	tree	21.915	101.275
Parashorea	chinensis	temperate grassland	tree	21.915	101.275
Pellacalyx	yunnanensis	temperate grassland	tree	21.915	101.275
Phlogacanthus	curviflorus	desert	forb	21.915	101.275
Phoebe	lanceolata	desert	tree	21.915	101.275
Pittosporopsis	kerrii	desert	tree	21.915	101.275
Poikilospermum	lanceolatum	desert	liana	21.915	101.275
Pometia	pinnata	desert	tree	21.915	101.275

Psychotria	calocarpa	temperate grassland	low to high shrub	21.915	101.275
Psychotria	henryi	temperate grassland	low to high shrub	21.915	101.275
Pterospermum	menglunense	temperate grassland	tree	21.915	101.275
Sterculia	brevissima	temperate grassland	low to high shrub	21.915	101.275
Stereospermum	colais	temperate grassland	tree	21.915	101.275
Syzygium	megacarpum	temperate grassland	tree	21.915	101.275
Tetrastigma	cauliflorum	desert	liana	21.915	101.275
Vitex	quinata	desert	tree	21.915	101.275
Xerospermum	bonii	desert	tree	21.915	101.275
Ziziphus	fungii	desert	liana	21.915	101.275
Actinodaphne	henryi	desert	tree	21.975	101.235
Alstonia	scholaris	desert	tree	21.975	101.235
Antidesma	acidum	desert	tree	21.975	101.235
Aporosa	yunnanensis	temperate shrubland	tree	21.975	101.235
Sapium	baccatum	temperate shrubland	tree	21.975	101.235
Oxyceros	sinensis	temperate shrubland	low to high shrub	21.975	101.235
Betula	alnoides	temperate shrubland	tree	21.975	101.235
Bridelia	stipularis	temperate shrubland	liana	21.975	101.235
Campylotropis	pinetorum	temperate shrubland	low to high shrub	21.975	101.235
Canthium	horridum	temperate shrubland	low to high shrub	21.975	101.235
Castanopsis	argyrophylla	temperate shrubland	tree	21.975	101.235
Castanopsis	hystrix	temperate shrubland	tree	21.975	101.235
Celastrus	paniculatus	desert	liana	21.975	101.235
Choerospondias	axillaris	desert	tree	21.975	101.235
Cibotium	barometz	desert	pteridophyte	21.975	101.235
Crassocephalum	crepidioides	desert	forb	21.975	101.235

Cratoxylon	cochinchinense	tropical evergreen broadleaf forest	tree	21.975	101.235
Dalbergia	pinnata	tropical evergreen broadleaf forest	liana	21.975	101.235
Dicranopteris	ampla	tropical evergreen broadleaf forest	pteridophyte	21.975	101.235
Engelhardia	spicata	tropical evergreen broadleaf forest	tree	21.975	101.235
Eurya	pittosporifolia	tropical evergreen broadleaf forest	low to high shrub	21.975	101.235
Fordia	cauliflora	tropical evergreen broadleaf forest	low to high shrub	21.975	101.235
Gnetum	montanum	tropical evergreen broadleaf forest	liana	21.975	101.235
Ilex	godajam	tropical evergreen broadleaf forest	tree	21.975	101.235
Leea	indica	tropical evergreen broadleaf forest	low to high shrub	21.975	101.235
Meliosma	arnottiana	tropical evergreen broadleaf forest	tree	21.975	101.235
Millettia	leptobotrya	tropical evergreen broadleaf forest	tree	21.975	101.235
Phoebe	lanceolata	tropical evergreen broadleaf forest	tree	21.975	101.235
Phoebe	puwenensis	tropical evergreen broadleaf forest	tree	21.975	101.235

Sarcosperma	arboreum	tropical evergreen broadleaf forest	tree	21.975	101.235
Saurauia	yunnanensis	tropical evergreen broadleaf forest	low to high shrub	21.975	101.235
Schima	wallichii	tropical evergreen broadleaf forest	tree	21.975	101.235
Smilax	hypoglauca	tropical evergreen broadleaf forest	liana	21.975	101.235
Tarennoidea	wallichii	tropical evergreen broadleaf forest	tree	21.975	101.235
Thysanolaena	latifolia	tropical evergreen broadleaf forest	graminoid	21.975	101.235
Toddalia	asiatica	tropical evergreen broadleaf forest	liana	21.975	101.235
Toona	ciliata	tropical evergreen broadleaf forest	tree	21.975	101.235
Toxicodendron	acuminatum	tropical evergreen broadleaf forest	tree	21.975	101.235
Trema	tomentosa	tropical evergreen broadleaf forest	tree	21.975	101.235
Turpinia	pomifera	tropical evergreen broadleaf forest	tree	21.975	101.235
Urena	lobata	tropical evergreen broadleaf forest	forb	21.975	101.235
Acanthus	leucostachyus	tropical evergreen broadleaf forest	forb	21.615	101.585

Alphonsea	monogyna	tropical evergreen broadleaf forest	liana	21.615	101.585
Amischotolype	hispida	tropical evergreen broadleaf forest	forb	21.615	101.585
Anthocephalus	chinensis	tropical evergreen broadleaf forest	tree	21.615	101.585
Antidesma	montanum	tropical evergreen broadleaf forest	tree	21.615	101.585
Aporosa	yunnanensis	tropical evergreen broadleaf forest	tree	21.615	101.585
Baccaurea	ramiflora	tropical evergreen broadleaf forest	tree	21.615	101.585
Boehmeria	clidemioides	tropical evergreen broadleaf forest	low to high shrub	21.615	101.585
Byttneria	grandifolia	tropical evergreen broadleaf forest	liana	21.615	101.585
Caesalpinia	coriaria	tropical evergreen broadleaf forest	liana	21.615	101.585
Capparis	fohaiensis	tropical evergreen broadleaf forest	liana	21.615	101.585
Castanopsis	indica	tropical evergreen broadleaf forest	tree	21.615	101.585
Cinnamomum	bejolghota	tropical evergreen broadleaf forest	tree	21.615	101.585
Cleidion	brevipetiolatum	tropical evergreen broadleaf forest	tree	21.615	101.585

Diospyros	hasseltii	tropical evergreen broadleaf forest	tree	21.615	101.585
Diospyros	nigrocortex	tropical evergreen broadleaf forest	tree	21.615	101.585
Duabanga	grandiflora	tropical evergreen broadleaf forest	tree	21.615	101.585
Dysoxylum	gotadhora	tropical evergreen broadleaf forest	tree	21.615	101.585
Elaeocarpus	glabripetalus	tropical evergreen broadleaf forest	tree	21.615	101.585
Elaeocarpus	rugosus	tropical evergreen broadleaf forest	tree	21.615	101.585
Garcinia	cowa	tropical shrubland	tree	21.615	101.585
Shorea	chinensis	tropical shrubland	tree	21.615	101.585
Knema	furfuracea	tropical shrubland	tree	21.615	101.585
Knema	globularia	tropical shrubland	tree	21.615	101.585
Leea	compactiflora	tropical shrubland	forb	21.615	101.585
Maesa	permollis	tropical shrubland	low to high shrub	21.615	101.585
Mitrephora	thorelii	tropical shrubland	tree	21.615	101.585
Mycetia	gracilis	tropical shrubland	low to high shrub	21.615	101.585
Mezzettiopsis	creaghii	tropical shrubland	tree	21.615	101.585
Ostodes	katharinae	tropical shrubland	tree	21.615	101.585
Phlogacanthus	curviflorus	tropical shrubland	forb	21.615	101.585
Phoebe	lanceolata	tropical shrubland	tree	21.615	101.585
Phrynium	placentarium	tropical shrubland	forb	21.615	101.585
Pittosporopsis	kerrii	tropical shrubland	low to high shrub	21.615	101.585

Pometia	pinnata	tropical shrubland	tree	21.615	101.585
Saprosma	ternatum	tropical shrubland	low to high shrub	21.615	101.585
Sloanea	tomentosa	tropical shrubland	tree	21.615	101.585
Smilax	zeylanica	tropical shrubland	liana	21.615	101.585
Syzygium	megacarpum	tropical shrubland	tree	21.615	101.585
Terminalia	myriocarpa	tropical shrubland	tree	21.615	101.585
Tetrastigma	planicaule	tropical shrubland	liana	21.615	101.585
Uncaria	laevigata	tropical shrubland	liana	21.615	101.585
Ardisia	thyrsiflora	tropical shrubland	tree	21.615	101.575
Baccaurea	ramiflora	tropical shrubland	tree	21.615	101.575
Beilschmiedia	purpurascens	tropical shrubland	tree	21.615	101.575
Castanopsis	echinocarpa	tropical shrubland	tree	21.615	101.575
Castanopsis	hystrix	tropical shrubland	tree	21.615	101.575
Cinnamomum	bejolghota	tropical shrubland	tree	21.615	101.575
Diospyros	sp.	tropical shrubland	tree	21.615	101.575
Elaeocarpus	sylvestris	tropical shrubland	tree	21.615	101.575
Elaeocarpus	varunua	tropical shrubland	tree	21.615	101.575
Engelhardia	spicata	tropical shrubland	tree	21.615	101.575
Garcinia	cowa	tropical shrubland	tree	21.615	101.575
Knema	furfuracea	tropical shrubland	tree	21.615	101.575
Lithocarpus	grandifolius	tropical shrubland	tree	21.615	101.575
Lithocarpus	sp.	tropical shrubland	tree	21.615	101.575
Litsea	verticillata	tropical shrubland	tree	21.615	101.575
Nephelium	chryseum	tropical shrubland	tree	21.615	101.575
Phoebe	lanceolata	tropical shrubland	tree	21.615	101.575
Polyalthia	simiarum	tropical shrubland	tree	21.615	101.575

Syzygium	oblatum	tropical shrubland	tree	21.615	101.575
Actinodaphne	henryi	tropical deciduous broadleaf forest	tree	21.615	101.585
Actinodaphne	obovata	tropical deciduous broadleaf forest	tree	21.615	101.585
Baccaurea	ramiflora	tropical deciduous broadleaf forest	tree	21.615	101.585
Bauhinia	erythropoda	tropical deciduous broadleaf forest	tree	21.615	101.585
Castanopsis	hystrix	tropical deciduous broadleaf forest	tree	21.615	101.585
Costus	speciosus	tropical deciduous broadleaf forest	forb	21.615	101.585
Desmos	yunnanensis	tropical deciduous broadleaf forest	liana	21.615	101.585
Ficus	cyrtophylla	tropical deciduous broadleaf forest	tree	21.615	101.585
Ficus	semicordata	tropical deciduous broadleaf forest	tree	21.615	101.585
Garcinia	cowa	tropical deciduous broadleaf forest	tree	21.615	101.585
Glochidion	lanceolarium	tropical deciduous broadleaf forest	tree	21.615	101.585
Gnetum	parvifolium	tropical deciduous broadleaf forest	liana	21.615	101.585

Goniothalamus	griffithii	tropical deciduous broadleaf forest	tree	21.615	101.585
Knema	furfuracea	tropical deciduous broadleaf forest	tree	21.615	101.585
Lasianthus	verticillatus	tropical deciduous broadleaf forest	low to high shrub	21.615	101.585
Leea	compactiflora	tropical deciduous broadleaf forest	forb	21.615	101.585
Lithocarpus	auriculatus	tropical deciduous broadleaf forest	tree	21.615	101.585
Litsea	monopetala	tropical deciduous broadleaf forest	tree	21.615	101.585
Melastoma	malabathricum	tropical deciduous broadleaf forest	low to high shrub	21.615	101.585
Mitrephora	thorelii	tropical deciduous broadleaf forest	tree	21.615	101.585
Phoebe	lanceolata	tropical deciduous broadleaf forest	tree	21.615	101.585
Phoebe	sheareri	tropical deciduous broadleaf forest	tree	21.615	101.585
Phrynium	placentarium	tropical deciduous broadleaf forest	forb	21.615	101.585
Pittosporopsis	kerrii	tropical deciduous broadleaf forest	low to high shrub	21.615	101.585
Poikilospermum	suaveolens	tropical deciduous broadleaf forest	liana	21.615	101.585

Pollia	thyrsiflora	tropical deciduous broadleaf forest	forb	21.615	101.585
Saprosma	ternatum	tropical deciduous broadleaf forest	low to high shrub	21.615	101.585
Sarcosperma	kachinense var. simondii	tropical deciduous broadleaf forest	tree	21.615	101.585
Schima	wallichii	tropical deciduous broadleaf forest	tree	21.615	101.585
Smilax	hypoglauca	tropical deciduous broadleaf forest	liana	21.615	101.585
Tabernaemontana	corymbosa	tropical deciduous broadleaf forest	low to high shrub	21.615	101.585
Tarennoidea	wallichii	tropical deciduous broadleaf forest	tree	21.615	101.585
Terminalia	myriocarpa	tropical deciduous broadleaf forest	tree	21.615	101.585
Thysanolaena	latifolia	tropical deciduous broadleaf forest	graminoid	21.615	101.585
Turpinia	pomifera	tropical deciduous broadleaf forest	tree	21.615	101.585
Uncaria	laevigata	tropical deciduous broadleaf forest	liana	21.615	101.585
Walsura	pinnata	tropical deciduous broadleaf forest	tree	21.615	101.585
Acer	campbellii	tropical deciduous broadleaf forest	tree	24.545	101.025

Actinidia	glaucocallosa	tropical deciduous broadleaf forest	liana	24.545	101.025
Ardisia	crenata	tropical deciduous broadleaf forest	low to high shrub	24.545	101.025
Aucuba	chlorascens	tropical deciduous broadleaf forest	low to high shrub	24.545	101.025
Camellia	forrestii var forrestii	tropical deciduous broadleaf forest	tree	24.545	101.025
Carex	perakensis	tropical deciduous broadleaf forest	graminoid	24.545	101.025
Castanopsis	wattii	tropical deciduous broadleaf forest	tree	24.545	101.025
Celastrus	orbiculatus	tropical deciduous broadleaf forest	liana	24.545	101.025
Daphne	papyracea var papyracea	tropical deciduous broadleaf forest	low to high shrub	24.545	101.025
Disporum	sessile	tropical deciduous broadleaf forest	forb	24.545	101.025
Dryopteris	wallichiana	tropical deciduous broadleaf forest	pteridophyte	24.545	101.025
Eriobotrya	bengalensis	tropical deciduous broadleaf forest	tree	24.545	101.025
Euonymus	vagans	tropical deciduous broadleaf forest	liana	24.545	101.025
Eurya	jintungensis	tropical deciduous broadleaf forest	tree	24.545	101.025

Fargesia	wuliangshanensis	tropical deciduous broadleaf forest	bamboo	24.545	101.025
Gamblea	ciliata var evodiifolia	tropical deciduous broadleaf forest	tree	24.545	101.025
Ilex	corallina	tropical deciduous broadleaf forest	tree	24.545	101.025
Ilex	gingtungensis	tropical deciduous broadleaf forest	tree	24.545	101.025
Illicium	simonsii	tropical deciduous broadleaf forest	tree	24.545	101.025
impatiens	rubrostriata	tropical deciduous broadleaf forest	forb	24.545	101.025
Lithocarpus	hancei	tropical deciduous broadleaf forest	tree	24.545	101.025
Lithocarpus	xylocarpus	tropical deciduous broadleaf forest	tree	24.545	101.025
Machilus	bombycina	tropical deciduous broadleaf forest	tree	24.545	101.025
Machilus	yunnanensis	tropical deciduous broadleaf forest	tree	24.545	101.025
Mahonia	duclouxiana	tropical deciduous broadleaf forest	low to high shrub	24.545	101.025
Manglietia	insignis	tropical deciduous broadleaf forest	tree	24.545	101.025
neolitsea	chuii	tropical deciduous broadleaf forest	tree	24.545	101.025

Plagiogyria	communis	tropical deciduous broadleaf forest	pteridophyte	24.545	101.025
Rhododendron	leptothrium	tropical deciduous broadleaf forest	small tree	24.545	101.025
Rosa	longicuspis	tropical deciduous broadleaf forest	liana	24.545	101.025
Rubus	paniculatus	tropical deciduous broadleaf forest	liana	24.545	101.025
Schima	noronhae	tropical deciduous broadleaf forest	tree	24.545	101.025
Stewartia	pteropetiolata	tropical deciduous broadleaf forest	tree	24.545	101.025
Symplocos	anomala	tropical deciduous broadleaf forest	tree	24.545	101.025
Symplocos	ramosissima	tropical deciduous broadleaf forest	tree	24.545	101.025
Symplocos	sumuntia	tropical deciduous broadleaf forest	tree	24.545	101.025
Ainsliaea	spicata	tropical deciduous broadleaf forest	forb	24.535	101.035
Carex	nemostachys	tropical deciduous broadleaf forest	graminoid	24.535	101.035
Clethra	delavayi var delavayi	tropical deciduous broadleaf forest	tree	24.535	101.035
Fargesia	wuliangshanensis	tropical deciduous broadleaf forest	bamboo	24.535	101.035

Gaultheria	griffithiana	tropical deciduous broadleaf forest	low to high shrub	24.535	101.035
Heterosmilax	chinensis	tropical deciduous broadleaf forest	liana	24.535	101.035
Ilex	corallina	tropical deciduous broadleaf forest	tree	24.535	101.035
Lithocarpus	crassifolius	tropical deciduous broadleaf forest	tree	24.535	101.035
Lithocarpus	grandifolius	tropical deciduous broadleaf forest	tree	24.535	101.035
Lyonia	ovalifolia	tropical deciduous broadleaf forest	tree	24.535	101.035
Lyonia	villosa	tropical deciduous broadleaf forest	tree	24.535	101.035
Pinus	armandii	tropical deciduous broadleaf forest	tree	24.535	101.035
Rhododendron	irroratum	tropical deciduous broadleaf forest	tree	24.535	101.035
Schefflera	fengii	tropical deciduous broadleaf forest	tree	24.535	101.035
Schefflera	shweliensis	tropical deciduous broadleaf forest	tree	24.535	101.035
Smilax	menispermoidea	tropical deciduous broadleaf forest	liana	24.535	101.035
Stranvaesia	davidiana	tropical deciduous broadleaf forest	tree	24.535	101.035

Symplocos	dryophila	tropical deciduous broadleaf forest	tree	24.535	101.035
Ternstroemia	gymnanthera	tropical deciduous broadleaf forest	tree	24.535	101.035
Vaccinium	duclouxii	tropical deciduous broadleaf forest	tree	24.535	101.035
Acystopteris	japonica	tropical deciduous broadleaf forest	pteridophyte	24.505	100.995
Alnus	nepalensis	tropical deciduous broadleaf forest	tree	24.505	100.995
Anneslea	fragrans	tropical deciduous broadleaf forest	tree	24.505	100.995
Camellia	forrestii var forrestii	tropical deciduous broadleaf forest	tree	24.505	100.995
Camellia	pitardii	tropical deciduous broadleaf forest	tree	24.505	100.995
Castanopsis	fleuryi	subtropical evergreen broadleaf forest	tree	24.505	100.995
Craibiodendron	yunnanense	subtropical evergreen broadleaf forest	tree	24.505	100.995
Dichroa	febrifuga	subtropical evergreen broadleaf forest	low to high shrub	24.505	100.995
Diploterygium	laevissimum	subtropical evergreen broadleaf forest	pteridophyte	24.505	100.995
Eurya	jintungensis	subtropical evergreen broadleaf forest	tree	24.505	100.995

Eurya	trichocarpa	subtropical evergreen broadleaf forest	tree	24.505	100.995
Hypericum	uralum	subtropical evergreen broadleaf forest	low to high shrub	24.505	100.995
Impatiens	rubrostriata	subtropical evergreen broadleaf forest	liana	24.505	100.995
Isodon	sculponeatus	subtropical evergreen broadleaf forest	liana	24.505	100.995
Leucosceptrum	canum	subtropical evergreen broadleaf forest	liana	24.505	100.995
Lithocarpus	dealbatus	subtropical evergreen broadleaf forest	tree	24.505	100.995
Lithocarpus	truncatus	subtropical evergreen broadleaf forest	tree	24.505	100.995
Lyonia	ovalifolia var lanceolata	subtropical evergreen broadleaf forest	tree	24.505	100.995
Lyonia	villosa	subtropical evergreen broadleaf forest	tree	24.505	100.995
Machilus	longipedicellata	subtropical evergreen broadleaf forest	tree	24.505	100.995
Maesa	indica	subtropical evergreen broadleaf forest	low to high shrub	24.505	100.995
Millettia	dielsiana	subtropical evergreen broadleaf forest	liana	24.505	100.995
Myrica	esculenta	subtropical evergreen broadleaf forest	tree	24.505	100.995

Pinus	kesiya	subtropical evergreen broadleaf forest	tree	24.505	100.995
Rhododendron	microphyton	subtropical evergreen broadleaf forest	tree	24.505	100.995
Rubus	alceifolius	subtropical evergreen broadleaf forest	liana	24.505	100.995
Schima	argentea	subtropical evergreen broadleaf forest	tree	24.505	100.995
Schima	wallichii	subtropical evergreen broadleaf forest	tree	24.505	100.995
Senecio	scandens	subtropical evergreen broadleaf forest	forb	24.505	100.995
Smilax	ocreata	subtropical evergreen broadleaf forest	liana	24.505	100.995
Ternstroemia	gymnanthera	subtropical evergreen broadleaf forest	tree	24.505	100.995
Tetrastigma	serrulatum	subtropical evergreen broadleaf forest	liana	24.505	100.995
Tripterygium	hypoglaucum	subtropical evergreen broadleaf forest	liana	24.505	100.995
Vaccinium	duclouxii	subtropical evergreen broadleaf forest	tree	24.505	100.995
Yushania	multiramea	subtropical evergreen broadleaf forest	bamboo	24.505	100.995
Albizia	kalkora	subtropical evergreen broadleaf forest	tree	23.685	101.855

Bothriochloa	pertusa	subtropical evergreen broadleaf forest	graminoid	23.685	101.855
Bridelia	tomentosa	subtropical evergreen broadleaf forest	low to high shrub	23.685	101.855
Buchanania	latifolia	subtropical evergreen broadleaf forest	tree	23.685	101.855
Cajanus	scarabaeoides	subtropical evergreen broadleaf forest	liana	23.685	101.855
Callicarpa	nudiflora	subtropical evergreen broadleaf forest	low to high shrub	23.685	101.855
Carissa	spinarum	subtropical evergreen broadleaf forest	low to high shrub	23.685	101.855
Cipadessa	baccifera	subtropical evergreen broadleaf forest	low to high shrub	23.685	101.855
Corallodiscus	flabellatus	subtropical evergreen broadleaf forest	forb	23.685	101.855
Crotalaria	linifolia	subtropical evergreen broadleaf forest	low to high shrub	23.685	101.855
Dendrolobium	triangulare	subtropical evergreen broadleaf forest	tree	23.685	101.855
Porana	racemosa	subtropical evergreen broadleaf forest	liana	23.685	101.855
Diospyros	yunnanensis	subtropical evergreen broadleaf forest	low to high shrub	23.685	101.855
Fraxinus	malacophylla	subtropical evergreen broadleaf forest	tree	23.685	101.855

Geodorum	densiflorum	subtropical evergreen broadleaf forest	geophyte	23.685	101.855
Heteropogon	contortus	subtropical evergreen broadleaf forest	graminoid	23.685	101.855
Isodon	amethystoides	subtropical evergreen broadleaf forest	erect dwarf shrub	23.685	101.855
Lannea	coromandelica	subtropical evergreen broadleaf forest	tree	23.685	101.855
Maytenus	hookeri	subtropical evergreen broadleaf forest	low to high shrub	23.685	101.855
Myriopteron	extensum	subtropical evergreen broadleaf forest	liana	23.685	101.855
Olea	cuspidata	subtropical evergreen broadleaf forest	low to high shrub	23.685	101.855
Osteomeles	schwerinae	subtropical evergreen broadleaf forest	low to high shrub	23.685	101.855
Parthenocissus	tricuspidata	subtropical evergreen broadleaf forest	liana	23.685	101.855
Phyllanthus	emblica	subtropical evergreen broadleaf forest	tree	23.685	101.855
Pistacia	weinmanniifolia	subtropical evergreen broadleaf forest	tree	23.685	101.855
Polyalthia	cerasoides	subtropical evergreen broadleaf forest	tree	23.685	101.855
Setaria	plicata	tropical shrubland	graminoid	23.685	101.855
Symplocos	racemosa	tropical shrubland	small tree	23.685	101.855

Tephrosia	purpurea	tropical shrubland	low to high shrub	23.685	101.855
Terminthia	paniculata	tropical shrubland	tree	23.685	101.855
Vitex	negundo	tropical shrubland	low to high shrub	23.685	101.855
Woodfordia	fruticosa	tropical shrubland	low to high shrub	23.685	101.855
Albizia	kalkora	tropical shrubland	tree	23.695	101.855
Barleria	cristata	tropical shrubland	low to high shrub	23.695	101.855
Boea	clarkeana	tropical shrubland	forb	23.695	101.855
Bombax	ceiba	tropical shrubland	tree	23.695	101.855
Bridelia	stipularis	tropical shrubland	liana	23.695	101.855
Buchanania	latifolia	tropical shrubland	tree	23.695	101.855
Caesalpinia	sappan	tropical shrubland	tree	23.695	101.855
Cajanus	scarabaeoides	tropical shrubland	liana	23.695	101.855
Callicarpa	nudiflora	tropical shrubland	low to high shrub	23.695	101.855
Campylotropis	delavayi	tropical shrubland	tree	23.695	101.855
Cipadessa	baccifera	tropical shrubland	low to high shrub	23.695	101.855
Corallodiscus	flabellatus	tropical shrubland	forb	23.695	101.855
Cryptolepis	buchananii	tropical shrubland	liana	23.695	101.855
Cyclobalanopsis	helferiana	tropical shrubland	tree	23.695	101.855
Dendrolobium	triangulare	tropical shrubland	tree	23.695	101.855
Porana	racemosa	tropical shrubland	liana	23.695	101.855
Eriobotrya	prinoides	tropical shrubland	tree	23.695	101.855
Eriolaena	spectabilis	tropical shrubland	tree	23.695	101.855
Fraxinus	malacophylla	tropical shrubland	tree	23.695	101.855
Garuga	forrestii	tropical shrubland	tree	23.695	101.855
Haldina	cordifolia	tropical shrubland	tree	23.695	101.855
Jasminum	diversifolium	tropical shrubland	liana	23.695	101.855

Lannea	coromandelica	tropical shrubland	tree	23.695	101.855
Myriopteron	extensum	tropical shrubland	liana	23.695	101.855
Olea	cuspidata	tropical shrubland	low to high shrub	23.695	101.855
Osteomeles	schwerinae	tropical shrubland	low to high shrub	23.695	101.855
Panicum	sumatrense	tropical shrubland	graminoid	23.695	101.855
Phyllanthus	emblica	tropical shrubland	tree	23.695	101.855
Pistacia	weinmanniifolia	tropical shrubland	tree	23.695	101.855
Polyalthia	cerasoides	tropical grassland	tree	23.695	101.855
Psidium	guajava	tropical grassland	tree	23.695	101.855
Setaria	plicata	tropical grassland	graminoid	23.695	101.855
Smilax	ferox	tropical grassland	liana	23.695	101.855
Symplocos	racemosa	tropical grassland	small tree	23.695	101.855
Tarenna	depauperata	tropical grassland	low to high shrub	23.695	101.855
Terminthia	paniculata	tropical grassland	tree	23.695	101.855
Trema	angustifolia	tropical grassland	tree	23.695	101.855
unknown	sp	tropical grassland	graminoid	23.695	101.855
Woodfordia	fruticosa	tropical grassland	low to high shrub	23.695	101.855
Acer	truncatum	tropical grassland	tree	36.765	109.245
Agropyron	cristatum	tropical grassland	graminoid	36.765	109.245
Amorpha	fruticosa	tropical grassland	forb	36.765	109.245
Artemisia	sacrorum	tropical grassland	erect dwarf shrub	36.765	109.245
Artemisia	subdigitata	tropical grassland	forb	36.765	109.245
Berberis	amurensis	tropical grassland	low to high shrub	36.765	109.245
Caragana	korshinskii	tropical grassland	low to high shrub	36.765	109.245
Carex	lanceolata	tropical grassland	graminoid	36.765	109.245
Cornus	macrophylla	tropical grassland	tree	36.765	109.245

Cotoneaster	acutifolius	tropical grassland	low to high shrub	36.765	109.245
Euphorbia	humifusa	tropical grassland	forb	36.765	109.245
Forsythia	suspensa	tropical grassland	low to high shrub	36.765	109.245
Hippophae	rhamnoides	tropical grassland	low to high shrub	36.765	109.245
Imperata	cylindrica var major	tropical grassland	graminoid	36.765	109.245
Lespedeza	davurica	tropical grassland	erect dwarf shub	36.765	109.245
Lonicera	hispida	tropical grassland	low to high shrub	36.765	109.245
Periploca	sepium	tropical grassland	low to high shrub	36.765	109.245
Pinus	tabuliformis	tropical grassland	tree	36.765	109.245
Populus	simonii	tropical grassland	tree	36.765	109.245
Potentilla	acaulis	tropical grassland	forb	36.765	109.245
Prinsepia	uniflora	tropical grassland	forb	36.765	109.245
Pulsatilla	chinensis	tropical grassland	forb	36.765	109.245
Pulsatilla	chinensis	tropical grassland	forb	36.765	109.245
Pyrus	betulifolia	tropical grassland	tree	36.765	109.245
Robinia	pseudoacacia	tropical grassland	tree	36.765	109.245
Rosa	hugonis	tropical grassland	low to high shrub	36.765	109.245
Rubus	parvifolius	tropical grassland	liana	36.765	109.245
Sophora	viciifolia	tropical grassland	low to high shrub	36.765	109.245
Spiraea	pubescens	tropical grassland	low to high shrub	36.765	109.245
Syringa	oblata	tropical grassland	small tree	36.765	109.245
Thalictrum	simplex	tropical grassland	forb	36.765	109.245
Ulmus	propinqua	tropical grassland	tree	36.765	109.245
Ulmus	pumila	tropical grassland	tree	36.765	109.245
Xanthoceras	sorbifolium	tropical grassland	small tree	36.765	109.245
Ziziphus	jujuba var spinosa	tropical grassland	small tree	36.765	109.245

Acer	erianthum	tropical grassland	tree	36.065	108.535
Acer	ginnala	tropical grassland	tree	36.065	108.535
Acer	truncatum	tropical grassland	tree	36.065	108.535
Agropyron	cristatum	tropical grassland	graminoid	36.065	108.535
Artemisia	giraldii	tropical grassland	erect dwarf shrub	36.065	108.535
Artemisia	sacrorum	tropical grassland	erect dwarf shrub	36.065	108.535
Artemisia	subdigitata	tropical grassland	forb	36.065	108.535
Betula	platyphylla var japonica	tropical grassland	tree	36.065	108.535
Bothriochloa	ischaemum	tropical grassland	erect dwarf shrub	36.065	108.535
Carex	lanceolata	tropical grassland	graminoid	36.065	108.535
Cornus	macrophylla	tropical grassland	tree	36.065	108.535
Cotoneaster	acutifolius	tropical grassland	low to high shrub	36.065	108.535
Elaeagnus	pungens	tropical grassland	low to high shrub	36.065	108.535
Acanthopanax	senticosus	tropical grassland	low to high shrub	36.065	108.535
Euphorbia	humifusa	tropical grassland	forb	36.065	108.535
Glycyrrhiza	uralensis	tropical grassland	low to high shrub	36.065	108.535
Hippophae	rhamnoides	tropical grassland	low to high shrub	36.065	108.535
Imperata	cylindrica var major	tropical grassland	graminoid	36.065	108.535
Lespedeza	davurica	tropical grassland	erect dwarf shub	36.065	108.535
Lonicera	hispida	tropical grassland	low to high shrub	36.065	108.535
Ostryopsis	davidiana	tropical grassland	low to high shrub	36.065	108.535
Periploca	sepium	tropical grassland	low to high shrub	36.065	108.535
Pinus	tabuliformis	tropical grassland	tree	36.065	108.535
Platycladus	orientalis	tropical grassland	tree	36.065	108.535
Populus	davidiana	tropical grassland	tree	36.065	108.535

Populus	simonii	tropical grassland	tree	36.065	108.535
Potentilla	acaulis	cropland	forb	36.065	108.535
Prinsepia	uniflora	cropland	forb	36.065	108.535
Prunus	davidiana	cropland	tree	36.065	108.535
Prunus	setulosa	cropland	tree	36.065	108.535
Pulsatilla	chinensis	cropland	forb	36.065	108.535
Pulsatilla	chinensis	cropland	forb	36.065	108.535
Pyrus	betulifolia	cropland	tree	36.065	108.535
Pyrus	pyrifolia	cropland	tree	36.065	108.535
Quercus	liaotungensis	cropland	tree	36.065	108.535
Robinia	pseudoacacia	cropland	tree	36.065	108.535
Rosa	hugonis	cropland	low to high shrub	36.065	108.535
Rubus	parvifolius	cropland	liana	36.065	108.535
Smilax	vaginata	cropland	low to high shrub	36.065	108.535
Sophora	viciifolia	cropland	low to high shrub	36.065	108.535
Sorbus	hupehensis var aperta	cropland	tree	36.065	108.535
Spiraea	pubescens	cropland	low to high shrub	36.065	108.535
Syringa	oblata	cropland	small tree	36.065	108.535
Thalictrum	simplex	cropland	forb	36.065	108.535
Ulmus	pumila	cropland	tree	36.065	108.535
Ziziphus	jujuba var spinosa	cropland	small tree	36.065	108.535
Agropyron	cristatum	cropland	graminoid	37.855	110.165
Amorpha	fruticosa	cropland	forb	37.855	110.165
Artemisia	giraldii	cropland	erect dwarf shrub	37.855	110.165
Artemisia	sacrorum	cropland	erect dwarf shrub	37.855	110.165

Artemisia	subdigitata	cropland	forb	37.855	110.165
Astragalus	adsurgens	cropland	forb	37.855	110.165
Caragana	korshinskii	cropland	low to high shrub	37.855	110.165
Pycnostelma	lateriflorum	cropland	forb	37.855	110.165
Lespedeza	bicolor	cropland	low to high shrub	37.855	110.165
Lespedeza	davurica	cropland	erect dwarf shub	37.855	110.165
Medicago	sativa	cropland	forb	37.855	110.165
Melilotus	albus	cropland	forb	37.855	110.165
Periploca	sepium	cropland	low to high shrub	37.855	110.165
Pinus	tabuliformis	cropland	tree	37.855	110.165
Populus	simonii	cropland	tree	37.855	110.165
Robinia	pseudoacacia	temperate evergreen needleleaf forest	tree	37.855	110.165
Robinia	pseudoacacia var inermis	temperate evergreen needleleaf forest	tree	37.855	110.165
Themeda	triandra var japonica	temperate evergreen needleleaf forest	graminoid	37.855	110.165
Ulmus	propinqua	temperate evergreen needleleaf forest	tree	37.855	110.165
Ulmus	pumila	temperate evergreen needleleaf forest	tree	37.855	110.165
Wikstroemia	chamaedaphne	temperate evergreen needleleaf forest	forb	37.855	110.165
Ziziphus	jujuba var spinosa	temperate evergreen needleleaf forest	small tree	37.855	110.165

Acer	erianthum	temperate evergreen needleleaf forest	tree	33.435	108.435
Acer	ginnala	temperate evergreen needleleaf forest	tree	33.435	108.435
Ampelopsis	aconitifolia	temperate evergreen needleleaf forest	liana	33.435	108.435
Anemone	vitifolia	temperate evergreen needleleaf forest	forb	33.435	108.435
Artemisia	argyi	temperate evergreen needleleaf forest	forb	33.435	108.435
Artemisia	sacrorum	temperate evergreen needleleaf forest	erect dwarf shrub	33.435	108.435
Artemisia	subdigitata	temperate evergreen needleleaf forest	forb	33.435	108.435
Astragalus	adsurgens	temperate evergreen needleleaf forest	forb	33.435	108.435
Axyris	amaranthoides	temperate evergreen needleleaf forest	forb	33.435	108.435
Berberis	circumserrata	temperate evergreen needleleaf forest	low to high shrub	33.435	108.435
Betula	platyphylla var japonica	temperate evergreen needleleaf forest	tree	33.435	108.435
Betula	albosinensis var septantrionalis	temperate evergreen needleleaf forest	tree	33.435	108.435
Carex	lanceolata	temperate evergreen needleleaf forest	graminoid	33.435	108.435

Clematis	obscura	temperate evergreen needleleaf forest	liana	33.435	108.435
Consolida	ajacis	temperate evergreen needleleaf forest	forb	33.435	108.435
Cornus	kousa var chinensis	temperate evergreen needleleaf forest	tree	33.435	108.435
Cornus	officinalis	temperate evergreen needleleaf forest	tree	33.435	108.435
Corylus	heterophylla	temperate evergreen needleleaf forest	tree	33.435	108.435
Cotoneaster	acutifolius	temperate evergreen needleleaf forest	low to high shrub	33.435	108.435
Pycnostelma	lateriflorum	temperate evergreen needleleaf forest	forb	33.435	108.435
Elaeagnus	glabra	temperate evergreen needleleaf forest	low to high shrub	33.435	108.435
Elaeagnus	pungens	temperate evergreen needleleaf forest	low to high shrub	33.435	108.435
Acanthopanax	senticosus	temperate evergreen needleleaf forest	low to high shrub	33.435	108.435
Euphorbia	humifusa	temperate evergreen needleleaf forest	forb	33.435	108.435
Forsythia	suspensa	temperate evergreen needleleaf forest	low to high shrub	33.435	108.435
Lespedeza	bicolor	temperate evergreen needleleaf forest	low to high shrub	33.435	108.435

Lespedeza	cyrtobotrya	temperate evergreen needleleaf forest	low to high shrub	33.435	108.435
Lonicera	hispida	temperate evergreen needleleaf forest	low to high shrub	33.435	108.435
Lonicera	japonica	temperate evergreen needleleaf forest	forb	33.435	108.435
Medicago	lupulina	temperate evergreen needleleaf forest	forb	33.435	108.435
Medicago	sativa	temperate evergreen needleleaf forest	forb	33.435	108.435
Melilotus	albus	temperate evergreen needleleaf forest	forb	33.435	108.435
Onobrychis	viciifolia	temperate evergreen needleleaf forest	forb	33.435	108.435
Paederia	scandens	temperate evergreen needleleaf forest	liana	33.435	108.435
Pinus	bungeana	temperate evergreen needleleaf forest	tree	33.435	108.435
Pinus	tabuliformis	temperate evergreen needleleaf forest	tree	33.435	108.435
Platycladus	orientalis	temperate evergreen needleleaf forest	tree	33.435	108.435
Populus	davidiana	temperate evergreen needleleaf forest	tree	33.435	108.435
Potentilla	acaulis	temperate evergreen needleleaf forest	forb	33.435	108.435

Potentilla	fruticosa	cropland	forb	33.435	108.435
Potentilla	multicaulis	cropland	forb	33.435	108.435
Prinsepia	uniflora	cropland	forb	33.435	108.435
Prunus	davidiana	cropland	tree	33.435	108.435
Pulsatilla	chinensis	cropland	forb	33.435	108.435
Pyrus	betulifolia	cropland	tree	33.435	108.435
Quercus	acutissima	cropland	tree	33.435	108.435
Quercus	aliena var acutiserrata	cropland	tree	33.435	108.435
Robinia	pseudoacacia	cropland	tree	33.435	108.435
Rosa	hugonis	cropland	low to high shrub	33.435	108.435
Rubus	innominatus	cropland	low to high shrub	33.435	108.435
Setaria	viridis	cropland	graminoid	33.435	108.435
Smilax	vaginata	cropland	low to high shrub	33.435	108.435
Spiraea	pubescens	cropland	low to high shrub	33.435	108.435
Syringa	oblata	cropland	small tree	33.435	108.435
Thalictrum	baicalense	cropland	forb	33.435	108.435
Thalictrum	foeniculaceum	cropland	forb	33.435	108.435
Vaccaria	pyramidata	cropland	forb	33.435	108.435
Agropyron	cristatum	cropland	graminoid	38.785	110.345
Astragalus	adsurgens	cropland	forb	38.785	110.345
Caragana	korshinskii	cropland	low to high shrub	38.785	110.345
Hippophae	rhamnoides	cropland	low to high shrub	38.785	110.345
Lespedeza	davurica	temperate broadleaf deciduous forest	erect dwarf shub	38.785	110.345
Medicago	sativa	temperate broadleaf deciduous forest	forb	38.785	110.345

Pinus	tabuliformis	temperate broadleaf deciduous forest	tree	38.785	110.345
Populus	simonii	temperate broadleaf deciduous forest	tree	38.785	110.345
Salix	mongolica	temperate broadleaf deciduous forest	tree	38.785	110.345
Ulmus	pumila	temperate broadleaf deciduous forest	tree	38.785	110.345
Ziziphus	jujuba var spinosa	temperate broadleaf deciduous forest	small tree	38.785	110.345
Acer	ginnala	temperate broadleaf deciduous forest	tree	38.785	110.345
Anemone	vitifolia	temperate broadleaf deciduous forest	forb	35.045	109.135
Artemisia	sacrorum	temperate broadleaf deciduous forest	erect dwarf shrub	35.045	109.135
Artemisia	subdigitata	temperate broadleaf deciduous forest	forb	35.045	109.135
Carex	lanceolata	temperate broadleaf deciduous forest	graminoid	35.045	109.135
Cornus	macrophylla	temperate broadleaf deciduous forest	tree	35.045	109.135
Cotoneaster	acutifolius	temperate broadleaf deciduous forest	low to high shrub	35.045	109.135
Elaeagnus	pungens	temperate broadleaf deciduous forest	low to high shrub	35.045	109.135

Forsythia	suspensa	temperate broadleaf deciduous forest	low to high shrub	35.045	109.135
Hippophae	rhamnoides	temperate broadleaf deciduous forest	low to high shrub	35.045	109.135
Lonicera	hispida	temperate broadleaf deciduous forest	low to high shrub	35.045	109.135
Ostryopsis	davidiana	temperate broadleaf deciduous forest	low to high shrub	35.045	109.135
Periploca	sepium	temperate broadleaf deciduous forest	low to high shrub	35.045	109.135
Pinus	tabuliformis	temperate broadleaf deciduous forest	tree	35.045	109.135
Populus	davidiana	temperate broadleaf deciduous forest	tree	35.045	109.135
Potentilla	acaulis	temperate broadleaf deciduous forest	forb	35.045	109.135
Prunus	davidiana	temperate broadleaf deciduous forest	tree	35.045	109.135
Prunus	pilosiuscula	temperate broadleaf deciduous forest	tree	35.045	109.135
Pulsatilla	chinensis	temperate broadleaf deciduous forest	forb	35.045	109.135
Pulsatilla	chinensis	temperate broadleaf deciduous forest	forb	35.045	109.135
Pyrus	betulifolia	temperate broadleaf deciduous forest	tree	35.045	109.135

Quercus	liaotungensis	temperate broadleaf deciduous forest	tree	35.045	109.135
Robinia	pseudoacacia	temperate broadleaf deciduous forest	tree	35.045	109.135
Rosa	hugonis	temperate broadleaf deciduous forest	low to high shrub	35.045	109.135
Sophora	viciifolia	temperate broadleaf deciduous forest	low to high shrub	35.045	109.135
Spiraea	pubescens	temperate broadleaf deciduous forest	low to high shrub	35.045	109.135
Syringa	oblata	temperate broadleaf deciduous forest	small tree	35.045	109.135
Thalictrum	simplex	temperate broadleaf deciduous forest	forb	35.045	109.135
Tilia	mongolica	temperate broadleaf deciduous forest	tree	35.045	109.135
Vaccaria	pyramidata	temperate broadleaf deciduous forest	forb	35.045	109.135
Ziziphus	jujuba var spinosa	temperate broadleaf deciduous forest	small tree	35.045	109.135
Artemisia	subdigitata	temperate broadleaf deciduous forest	forb	34.265	108.065
Atriplex	littoralis	temperate broadleaf deciduous forest	erect dwarf shrub	34.265	108.065
Diospyros	kaki	temperate broadleaf deciduous forest	tree	34.265	108.065

Lespedeza	davurica	temperate broadleaf deciduous forest	erect dwarf shub	34.265	108.065
Pinus	tabuliformis	temperate broadleaf deciduous forest	tree	34.265	108.065
Platycladus	orientalis	temperate broadleaf deciduous forest	tree	34.265	108.065
Robinia	pseudoacacia	temperate broadleaf deciduous forest	tree	34.265	108.065
Rubus	parvifolius	temperate broadleaf deciduous forest	liana	34.265	108.065
Ziziphus	jujuba var spinosa	temperate broadleaf deciduous forest	small tree	34.265	108.065
Ampelopsis	aconitifolia	temperate broadleaf deciduous forest	liana	34.815	108.035
Artemisia	argyi	temperate broadleaf deciduous forest	forb	34.815	108.035
Artemisia	sacrorum	temperate broadleaf deciduous forest	erect dwarf shrub	34.815	108.035
Artemisia	subdigitata	temperate broadleaf deciduous forest	forb	34.815	108.035
Hippophae	rhamnoides	temperate broadleaf deciduous forest	low to high shrub	34.815	108.035
Lespedeza	davurica	temperate broadleaf deciduous forest	erect dwarf shub	34.815	108.035
Lonicera	koehneana	temperate broadleaf deciduous forest	low to high shrub	34.815	108.035

Lonicera	japonica	temperate broadleaf deciduous forest	forb	34.815	108.035
Pinus	tabuliformis	temperate broadleaf deciduous forest	tree	34.815	108.035
Populus	hopeiensis	temperate broadleaf deciduous forest	tree	34.815	108.035
Potentilla	acaulis	steppe	forb	34.815	108.035
Pulsatilla	chinensis	steppe	forb	34.815	108.035
Rhamnus	arguta	steppe	tree	34.815	108.035
Robinia	pseudoacacia	steppe	tree	34.815	108.035
Rosa	hugonis	steppe	low to high shrub	34.815	108.035
Rubus	parvifolius	steppe	liana	34.815	108.035
Spiraea	pubescens	steppe	low to high shrub	34.815	108.035
Thalictrum	simplex	steppe	forb	34.815	108.035
Vaccaria	pyramidata	steppe	forb	34.815	108.035
Ziziphus	jujuba var spinosa	steppe	small tree	34.815	108.035
Baccaurea	ramiflora	steppe	tree	21.935	101.25
Barringtonia	macrostachya	steppe	tree	21.935	101.25
Bauhinia	glauca	cropland	low to high shrub	21.935	101.25
Bauhinia	yunnanensis	cropland	liana	21.935	101.25
Byttneria	aspera	cropland	liana	21.935	101.25
Millettia	oosperma	cropland	low to high shrub	21.935	101.25
Carallia	lanceaefolia	cropland	tree	21.935	101.25
Castanopsis	indica	cropland	tree	21.935	101.25
Celastrus	paniculatus	cropland	liana	21.935	101.25
Combretum	latifolium	cropland	liana	21.935	101.25

Ficus	auriculata	cropland	tree	21.935	101.25
Ficus	callosa	cropland	tree	21.935	101.25
Ficus	cyrtophylla	cropland	tree	21.935	101.25
Ficus	hirta	cropland	tree	21.935	101.25
Ficus	subulata	cropland	liana	21.935	101.25
Ficus	superba	cropland	tree	21.935	101.25
Fissistigma	polyanthoides	cropland	low to high shrub	21.935	101.25
Fissistigma	polyanthum	cropland	low to high shrub	21.935	101.25
Gnetum	parvifolium	cropland	liana	21.935	101.25
Shorea	chinensis	cropland	tree	21.935	101.25
Iodes	ovalis	cropland	forb	21.935	101.25
Leea	crispa	cropland	low to high shrub	21.935	101.25
Lepisanthes	senegalensis	cropland	tree	21.935	101.25
Litsea	panamanja	cropland	low to high shrub	21.935	101.25
Mayodendron	igneum	cropland	tree	21.935	101.25
Millettia	dielsiana	cropland	liana	21.935	101.25
Securidaca	inappendiculata	cropland	low to high shrub	21.935	101.25
Syzygium	latilimbum	cropland	tree	21.935	101.25
Tetrastigma	planicaule	cropland	liana	21.935	101.25
Tinomiscium	tonkinensis	cropland	liana	21.935	101.25
Uncaria	macrophylla	cropland	liana	21.935	101.25
Uncaria	rhynchophylla	cropland	liana	21.935	101.25
Ventilago	calyculata	cropland	liana	21.935	101.25
Ziziphus	attopensis	cropland	liana	21.935	101.25
Acer	buergerianum	cropland	tree	32.065	118.815
Acer	ginnala	cropland	tree	32.065	118.815

Broussonetia	papyrifera	cropland	tree	32.065	118.815
Celtis	sinensis	cropland	tree	32.065	118.815
Dalbergia	hupeana	cropland	tree	32.065	118.815
Diospyros	lotus	cropland	tree	32.065	118.815
Securinega	suffruticosa	cropland	tree	32.065	118.815
Grewia	biloba	temperate grassland	low to high shrub	32.065	118.815
Ligustrum	lucidum	temperate grassland	tree	32.065	118.815
Ligustrum	quihoui	temperate grassland	low to high shrub	32.065	118.815
Lindera	angustifolia	temperate grassland	low to high shrub	32.065	118.815
Lindera	glauca	temperate grassland	low to high shrub	32.065	118.815
Liquidambar	formosana	temperate grassland	tree	32.065	118.815
Cudrania	tricuspidata	temperate grassland	low to high shrub	32.065	118.815
Morus	alba	temperate grassland	small tree	32.065	118.815
Pistacia	chinensis	temperate grassland	tree	32.065	118.815
Platycarya	strobilacea	temperate grassland	tree	32.065	118.815
Pueraria	lobata	temperate grassland	liana	32.065	118.815
Quercus	fabrei	temperate grassland	tree	32.065	118.815
Rhamnus	crenata	temperate grassland	low to high shrub	32.065	118.815
Rosa	multiflora	temperate grassland	low to high shrub	32.065	118.815
Rubus	parvifolius	temperate grassland	liana	32.065	118.815
Smilax	china	temperate grassland	liana	32.065	118.815
Symplocos	paniculata	temperate grassland	low to high shrub	32.065	118.815
Ulmus	parvifolia	temperate grassland	tree	32.065	118.815
Vitex	negundo	temperate grassland	low to high shrub	32.065	118.815
Wisteria	sinensis	temperate grassland	liana	32.065	118.815
Acer	buergerianum	cropland	tree	32.045	118.835

Acer	ginnala	cropland	tree	32.045	118.835
Aphananthe	aspera	cropland	tree	32.045	118.835
Celtis	sinensis	cropland	tree	32.045	118.835
Dalbergia	hupeana	cropland	tree	32.045	118.835
Diospyros	kaki	cropland	tree	32.045	118.835
Elaeagnus	multiflora	cropland	low to high shrub	32.045	118.835
Euonymus	alatus	cropland	low to high shrub	32.045	118.835
Euscaphis	japonica	cropland	low to high shrub	32.045	118.835
Firmiana	simplex	cropland	tree	32.045	118.835
Securinega	suffruticosa	cropland	tree	32.045	118.835
Grewia	biloba	cropland	low to high shrub	32.045	118.835
Kalopanax	septemlobus	cropland	tree	32.045	118.835
Ligustrum	lucidum	cropland	tree	32.045	118.835
Lindera	glauca	cropland	low to high shrub	32.045	118.835
Liquidambar	formosana	cropland	tree	32.045	118.835
Lonicera	japonica	cropland	forb	32.045	118.835
Cudrania	tricuspidata	cropland	low to high shrub	32.045	118.835
Magnolia	denudata	cropland	tree	32.045	118.835
Desmodium	caudatum	cropland	low to high shrub	32.045	118.835
Photinia	parvifolia	cropland	tree	32.045	118.835
Pistacia	chinensis	cropland	tree	32.045	118.835
Premna	microphylla	cropland	erect dwarf shrub	32.045	118.835
Quercus	fabrei	cropland	tree	32.045	118.835
Quercus	variabilis	cropland	tree	32.045	118.835
Rhamnus	globosa	cropland	low to high shrub	32.045	118.835
Rhus	chinensis	cropland	tree	32.045	118.835

Rubus	swinhoei	cropland	low to high shrub	32.045	118.835
Serissa	serissoides	cropland	erect dwarf shrub	32.045	118.835
Smilax	china	cropland	liana	32.045	118.835
Symplocos	paniculata	cropland	low to high shrub	32.045	118.835
Ulmus	parvifolia	cropland	tree	32.045	118.835
Viburnum	dilatatum	cropland	low to high shrub	32.045	118.835
Agriophyllum	squarrosum	cropland	forb	36.425	80.715
Artemisia	sp.	tropical evergreen needleleaf forest	ND	36.425	80.715
Bassia	dasyphylla	tropical evergreen needleleaf forest	forb	36.425	80.715
Calligonum	mongolicum	tropical evergreen needleleaf forest	low to high shrub	36.425	80.715
Corispermum	heptapotamicum	tropical evergreen needleleaf forest	forb	36.425	80.715
Halogeton	glomeratus	tropical evergreen needleleaf forest	erect dwarf shrub	36.425	80.715
Ceratoides	latens	tropical evergreen needleleaf forest	erect dwarf shrub	36.425	80.715
Reaumuria	soongarica	tropical evergreen needleleaf forest	low to high shrub	36.425	80.715
Salsola	ruthenica	tropical evergreen needleleaf forest	forb	36.425	80.715
Sympegma	regelii	tropical evergreen needleleaf forest	erect dwarf shrub	36.425	80.715

Zygophyllum	xanthoxylum	tropical evergreen needleleaf forest	erect dwarf shrub	36.425	80.715
Agriophyllum	squarrosum	tropical evergreen needleleaf forest	forb	44.365	87.915
Astragalus	oxyglottis	tropical evergreen needleleaf forest	forb	44.365	87.915
Bassia	dasyphylla	tropical evergreen needleleaf forest	forb	44.365	87.915
Ceratocarpus	arenarius	tropical evergreen needleleaf forest	erect dwarf shrub	44.365	87.915
Eragrostis	poaeoides	tropical evergreen needleleaf forest	graminoid	44.365	87.915
Horanowia	ulicina	tropical evergreen needleleaf forest	forb	44.365	87.915
Nitraria	sibirica	tropical evergreen needleleaf forest	low to high shrub	44.365	87.915
Petrosimonia	sibirica	tropical evergreen needleleaf forest	forb	44.365	87.915
Reaumuria	soongarica	tropical evergreen needleleaf forest	low to high shrub	44.365	87.915
Salsola	sp.a	tropical evergreen needleleaf forest	ND	44.365	87.915
Salsola	sp.b	tropical evergreen needleleaf forest	ND	44.365	87.915
Salsola	sp.c	tropical evergreen needleleaf forest	ND	44.365	87.915

Seriphidium	terrae-albae	tropical evergreen needleleaf forest	erect dwarf shrub	44.365	87.915
Aristida	pennata	tropical evergreen needleleaf forest	graminoid	44.365	87.915
Allium	mongolicum	tropical evergreen needleleaf forest	geophyte	37.455	104.775
Artemisia	capillaris	tropical evergreen needleleaf forest	low to high shrub	37.455	104.775
Artemisia	ordosica	tropical evergreen needleleaf forest	low to high shrub	37.455	104.775
Cleistogenes	songorica	tropical evergreen needleleaf forest	graminoid	37.455	104.775
Ceratoides	latens	subtropical decidous broadleaf forest	erect dwarf shrub	37.455	104.775
Lespedeza	davurica	subtropical decidous broadleaf forest	erect dwarf shub	37.455	104.775
Nitraria	sibirica	subtropical decidous broadleaf forest	low to high shrub	37.455	104.775
Salsola	laricifolia	subtropical decidous broadleaf forest	erect dwarf shrub	37.455	104.775
Salsola	passerina	subtropical decidous broadleaf forest	erect dwarf shrub	37.455	104.775
Stipa	gobica	subtropical decidous broadleaf forest	graminoid	37.455	104.775
Agriophyllum	squarrosum	subtropical decidous broadleaf forest	forb	39.495	110.205

Artemisia	ordosica	subtropical decidous broadleaf forest	low to high shrub	39.495	110.205
Artemisia	sphaerocephala	subtropical decidous broadleaf forest	forb	39.495	110.205
Astragalus	melilotoides	subtropical decidous broadleaf forest	forb	39.495	110.205
Bassia	dasyphylla	subtropical decidous broadleaf forest	forb	39.495	110.205
Caragana	korshinskii	subtropical decidous broadleaf forest	low to high shrub	39.495	110.205
Cleistogenes	squarrosa	subtropical decidous broadleaf forest	graminoid	39.495	110.205
Corispermum	mongolicum	subtropical decidous broadleaf forest	forb	39.495	110.205
Cynanchum	thesioides	subtropical decidous broadleaf forest	forb	39.495	110.205
Chenopodium	aristatum	subtropical decidous broadleaf forest	forb	39.495	110.205
Euphorbia	humifusa	subtropical decidous broadleaf forest	forb	39.495	110.205
Ixeris	gracilis	subtropical decidous broadleaf forest	forb	39.495	110.205
Lespedeza	davurica	subtropical decidous broadleaf forest	erect dwarf shub	39.495	110.205
Oxytropis	psammocharis	subtropical decidous broadleaf forest	forb	39.495	110.205

Poa	annua	subtropical decidous broadleaf forest	graminoid	39.495	110.205
Setaria	viridis	subtropical decidous broadleaf forest	graminoid	39.495	110.205
Agropyron	cristatum	subtropical decidous broadleaf forest	graminoid	42.865	115.885
Agropyron	desertorum	subtropical decidous broadleaf forest	graminoid	42.865	115.885
Agropyron	mongolicum	subtropical decidous broadleaf forest	graminoid	42.865	115.885
Artemisia	frigida	subtropical decidous broadleaf forest	erect dwarf shrub	42.865	115.885
Artemisia	intramongolica	subtropical decidous broadleaf forest	low to high shrub	42.865	115.885
Artemisia	scoparia	subtropical decidous broadleaf forest	forb	42.865	115.885
Calamagrostis	epigejos	subtropical decidous broadleaf forest	graminoid	42.865	115.885
Caragana	korshinskii	subtropical decidous broadleaf forest	low to high shrub	42.865	115.885
Chenopodium	acuminatum	subtropical decidous broadleaf forest	forb	42.865	115.885
Cleistogenes	squarrosa	subtropical decidous broadleaf forest	graminoid	42.865	115.885
Corispermum	mongolicum	desert	forb	42.865	115.885
Cynanchum	thesioides	desert	forb	42.865	115.885

Inula	britannica	desert	forb	42.865	115.885
Oxytropis	psammocharis	desert	forb	42.865	115.885
Potentilla	acaulis	desert	forb	42.865	115.885
Salsola	collina	desert	forb	42.865	115.885
Thalictrum	squarrosum	desert	forb	42.865	115.885
Thymus	mongolicus	desert	erect dwarf shrub	42.865	115.885
Agriophyllum	squarrosum	desert	forb	42.865	115.885
Artemisia	halodendron	desert	forb	42.935	120.685
Bassia	dasyphylla	desert	forb	42.935	120.685
Caragana	microphylla	desert	low to high shrub	42.935	120.685
Chenopodium	acuminatum	desert	forb	42.935	120.685
Cleistogenes	squarrosa	desert	graminoid	42.935	120.685
Corispermum	mongolicum	desert	forb	42.935	120.685
Cynanchum	thesioides	desert	forb	42.935	120.685
Echinochloa	crusgalli	desert	graminoid	42.935	120.685
Euphorbia	humifusa	desert	forb	42.935	120.685
Ixeris	gracilis	desert	forb	42.935	120.685
Lespedeza	davurica	desert	erect dwarf shub	42.935	120.685
Lespedeza	juncea	desert	erect dwarf shrub	42.935	120.685
Melissitus	ruthenicus	desert	forb	42.935	120.685
Oxytropis	psammocharis	desert	forb	42.935	120.685
Salsola	collina	desert	forb	42.935	120.685
Setaria	viridis	desert	graminoid	42.935	120.685
Sophora	flavescens	desert	forb	42.935	120.685
Agriophyllum	squarrosum	desert	forb	49.475	117.955
Agropyron	cristatum	desert	graminoid	49.475	117.955

Artemisia	frigida	desert	erect dwarf shrub	49.475	117.955
Artemisia	halodendron	desert	forb	49.475	117.955
Astragalus	melilotoides	desert	forb	49.475	117.955
Chenopodium	acuminatum	desert	forb	49.475	117.955
Cleistogenes	squarrosa	desert	graminoid	49.475	117.955
Corispermum	mongolicum	desert	forb	49.475	117.955
Cynanchum	thesioides	desert	forb	49.475	117.955
Hedysarum	fruticosum	steppe	forb	49.475	117.955
Oxytropis	hailarensis	steppe	forb	49.475	117.955
Rhodiola	rosea	steppe	forb	49.475	117.955
Salsola	collina	steppe	forb	49.475	117.955
Setaria	viridis	steppe	graminoid	49.475	117.955
Abies	nephrolepis	steppe	tree	42.315	128.125
Acer	mandshuricum	steppe	tree	42.315	128.125
Acer	mono	steppe	tree	42.315	128.125
Acer	pseudo-sieboldianum	steppe	tree	42.315	128.125
Acer	tegmentosum	steppe	tree	42.315	128.125
Acer	ukurundense	steppe	tree	42.315	128.125
Corylus	mandshurica	steppe	tree	42.315	128.125
Deutzia	parviflora var. amurensis	steppe	low to high shrub	42.315	128.125
Eleutherococcus	senticosus	steppe	low to high shrub	42.315	128.125
Fraxinus	mandshurica	steppe	tree	42.315	128.125
Larix	olgensis	steppe	tree	42.315	128.125

Lonicera	praeflorens	temperate deciduous woodland	low to high shrub	42.315	128.125
Maackia	amurensis	temperate deciduous woodland	tree	42.315	128.125
Philadelphus	schrenkii	temperate deciduous woodland	low to high shrub	42.315	128.125
Pinus	koraiensis	temperate deciduous woodland	tree	42.315	128.125
Quercus	mongolica	temperate deciduous woodland	tree	42.315	128.125
Rhamnus	davurica	temperate deciduous woodland	tree	42.315	128.125
Ribes	maximoviczianum	temperate deciduous woodland	low to high shrub	42.315	128.125
Sorbaria	sorbifolia	temperate deciduous woodland	low to high shrub	42.315	128.125
Spiraea	chamaedrifolia	temperate deciduous woodland	low to high shrub	42.315	128.125
Syringa	reticulata subsp. amurensis	temperate deciduous woodland	tree	42.315	128.125
Tilia	amurensis	temperate deciduous woodland	tree	42.315	128.125
Ulmus	davidiana var. japonica	temperate deciduous woodland	tree	42.315	128.125
Ulmus	laciniata	temperate deciduous woodland	tree	42.315	128.125

Abies	nephrolepis	temperate deciduous woodland	tree	42.285	128.095
Acer	mono	temperate deciduous woodland	tree	42.285	128.095
Acer	pseudo-sieboldianum	temperate deciduous woodland	tree	42.285	128.095
Acer	tegmentosum	temperate deciduous woodland	tree	42.285	128.095
Acer	ukurundense	temperate deciduous woodland	tree	42.285	128.095
Corylus	mandshurica	temperate deciduous woodland	tree	42.285	128.095
Eleutherococcus	senticosus	steppe	low to high shrub	42.285	128.095
Euonymus	verrucosus	steppe	low to high shrub	42.285	128.095
Lonicera	praeflorens	steppe	low to high shrub	42.285	128.095
Padus	avium	steppe	tree	42.285	128.095
Philadelphus	schrenkii	steppe	low to high shrub	42.285	128.095
Pinus	koraiensis	steppe	tree	42.285	128.095
Populus	davidiana	steppe	tree	42.285	128.095
Quercus	mongolica	steppe	tree	42.285	128.095
Ribes	mandshuricum	steppe	low to high shrub	42.285	128.095
Syringa	reticulata subsp. amurensis	steppe	tree	42.285	128.095
Tilia	amurensis	steppe	tree	42.285	128.095
Ulmus	davidiana var. japonica	steppe	tree	42.285	128.095

Viburnum	burejaeticum	steppe	low to high shrub	42.285	128.095
Abies	nephrolepis	steppe	tree	42.225	128.075
Acer	komarovii/tschonoskii	steppe	tree	42.225	128.075
Acer	mono	steppe	tree	42.225	128.075
Acer	pseudo-<br>sieboldianum	steppe	tree	42.225	128.075
Acer	tegmentosum	steppe	tree	42.225	128.075
Acer	ukurundense	steppe	tree	42.225	128.075
Corylus	mandshurica	steppe	tree	42.225	128.075
Eleutherococcus	senticosus	steppe	low to high shrub	42.225	128.075
Lonicera	praeflorens	steppe	low to high shrub	42.225	128.075
Philadelphus	schrenkii	steppe	low to high shrub	42.225	128.075
Ribes	mandshuricum	steppe	low to high shrub	42.225	128.075
Tilia	amurensis	steppe	tree	42.225	128.075
Abies	nephrolepis	steppe	tree	42.175	128.135
Acer	barbinerve	steppe	tree	42.175	128.135
Acer	komarovii/tschonoskii	steppe	tree	42.175	128.135
Acer	tegmentosum	steppe	tree	42.175	128.135
Acer	ukurundense	steppe	tree	42.175	128.135
Berberis	amurensis	steppe	low to high shrub	42.175	128.135
Euonymus	verrucosus	temperate mixed forest	low to high shrub	42.175	128.135
Larix	olgensis	temperate mixed forest	tree	42.175	128.135
Lonicera	edulis	temperate mixed forest	low to high shrub	42.175	128.135
Lonicera	maximowiczii	temperate mixed forest	low to high shrub	42.175	128.135
Picea	jezoensis var.<br>komarovii	temperate mixed forest	tree	42.175	128.135

Picea	koraiensis	temperate mixed forest	tree	42.175	128.135
Pinus	koraiensis	temperate mixed forest	tree	42.175	128.135
Ribes	maximoviczianum	temperate mixed forest	low to high shrub	42.175	128.135
Rosa	acicularis	temperate mixed forest	low to high shrub	42.175	128.135
Tilia	amurensis	temperate mixed forest	tree	42.175	128.135
Vaccinium	uliginosum var. alpinum	temperate mixed forest	low to high shrub	42.175	128.135
Abies	nephrolepis	temperate mixed forest	tree	42.125	128.115
Acer	komarovii/tschonoskii	temperate mixed forest	tree	42.125	128.115
Acer	tegmentosum	temperate mixed forest	tree	42.125	128.115
Acer	ukurundense	temperate mixed forest	tree	42.125	128.115
Euonymus	verrucosus	temperate mixed forest	low to high shrub	42.125	128.115
Larix	olgensis	temperate mixed forest	tree	42.125	128.115
Lonicera	edulis	temperate mixed forest	low to high shrub	42.125	128.115
Picea	jezoensis var. komarovii	temperate mixed forest	tree	42.125	128.115
Pinus	koraiensis	temperate mixed forest	tree	42.125	128.115
Ribes	maximoviczianum	temperate mixed forest	low to high shrub	42.125	128.115
Rosa	acicularis	temperate mixed forest	low to high shrub	42.125	128.115
Abies	nephrolepis	temperate mixed forest	tree	42.085	128.075
Acer	komarovii/tschonoskii	temperate mixed forest	tree	42.085	128.075
Acer	ukurundense	temperate mixed forest	tree	42.085	128.075
Lonicera	edulis	temperate mixed forest	low to high shrub	42.085	128.075
Lonicera	maximowiczii	temperate mixed forest	low to high shrub	42.085	128.075
Picea	jezoensis var. komarovii	temperate mixed forest	tree	42.085	128.075

Ribes	horridum	temperate mixed forest	low to high shrub	42.085	128.075
Rosa	acicularis	temperate mixed forest	low to high shrub	42.085	128.075
Abies	nephrolepis	temperate mixed forest	tree	42.075	128.065
Acer	ukurundense	temperate mixed forest	tree	42.075	128.065
Betula	ermanii	temperate mixed forest	tree	42.075	128.065
Larix	olgensis	temperate mixed forest	tree	42.075	128.065
Lonicera	edulis	temperate mixed forest	low to high shrub	42.075	128.065
Lonicera	maximowiczii	temperate mixed forest	low to high shrub	42.075	128.065
Picea	jezoensis var. komarovii	temperate mixed forest	tree	42.075	128.065
Rhododendron	aureum	temperate mixed forest	erect dwarf shrub	42.075	128.065
Ribes	horridum	temperate mixed forest	low to high shrub	42.075	128.065
Sorbus	pohuashanensis	temperate mixed forest	tree	42.075	128.065
Viburnum	koreanum	temperate mixed forest	low to high shrub	42.075	128.065
Betula	ermanii	temperate mixed forest	tree	42.065	128.065
Larix	olgensis	temperate mixed forest	tree	42.065	128.065
Lonicera	edulis	boreal needleleaf forest	low to high shrub	42.065	128.065
Rhododendron	aureum	boreal needleleaf forest	erect dwarf shrub	42.065	128.065
Sorbus	pohuashanensis	boreal needleleaf forest	tree	42.065	128.065
Alnus	hirsuta	boreal needleleaf forest	small tree	42.065	128.065
Betula	fruticosa	boreal needleleaf forest	low to high shrub	53.465	122.345

Betula	platyphylla	boreal needleleaf forest	tree	53.465	122.345
Calamagrostis	angustifolia	boreal needleleaf forest	graminoid	53.465	122.345
Equisetum	arvense	boreal needleleaf forest	pteridophyte	53.465	122.345
Geranium	wilfordii	boreal needleleaf forest	forb	53.465	122.345
Larix	gmelinii	boreal needleleaf forest	tree	53.465	122.345
Ledum	palustre	boreal needleleaf forest	erect dwarf shrub	53.465	122.345
Phedimus	aizoon	boreal needleleaf forest	forb	53.465	122.345
Pinus	sylvestris var. mongolica	boreal needleleaf forest	tree	53.465	122.345
Potentilla	fruticosa	boreal needleleaf forest	erect dwarf shrub	53.465	122.345
Rhododendron	simsii	boreal needleleaf forest	low to high shrub	53.465	122.345
Rosa	acicularis	boreal needleleaf forest	low to high shrub	53.465	122.345
Salix	raddeana	boreal needleleaf forest	low to high shrub	53.465	122.345
Saussurea	japonica	boreal needleleaf forest	rosette forb	53.465	122.345

Thalictrum	aquilegiifolium	boreal needleleaf forest	forb	53.465	122.345
Trollius	chinensis	boreal needleleaf forest	forb	53.465	122.345
Vaccinium	uliginosum	boreal needleleaf forest	erect dwarf shrub	53.465	122.345
Vaccinium	vitis-idaea	boreal needleleaf forest	erect dwarf shrub	53.465	122.345
Vicia	sepium	boreal needleleaf forest	climber	53.465	122.345
Adenophora	tetraphylla	boreal needleleaf forest	forb	53.465	122.345
Alnus	hirsuta	boreal needleleaf forest	tree	53.465	122.345
Betula	platyphylla	boreal needleleaf forest	tree	53.465	122.345
Calamagrostis	angustifolia	boreal needleleaf forest	graminoid	53.465	122.345
Equisetum	arvense	boreal needleleaf forest	pteridophyte	53.465	122.345
Fragaria	orientalis	boreal needleleaf forest	forb	53.465	122.345
Geranium	wilfordii	boreal needleleaf forest	forb	53.465	122.345
Larix	gmelinii	boreal needleleaf forest	tree	53.465	122.345

Pinus	sylvestris var. mongolica	boreal needleleaf forest	tree	53.465	122.345
Populus	davidiana	boreal needleleaf forest	tree	53.465	122.345
Pyrola	asarifolia	boreal needleleaf forest	rosette forb	53.465	122.345
Rhododendron	simsii	boreal needleleaf forest	low to high shrub	53.465	122.345
Ribes	mandshuricum	boreal needleleaf forest	low to high shrub	53.465	122.345
Rosa	acicularis	boreal needleleaf forest	low to high shrub	53.465	122.345
Salix	raddeana	boreal needleleaf forest	small tree	53.465	122.345
Sanguisorba	officinalis	boreal needleleaf forest	forb	53.465	122.345
Sorbaria	sorbifolia	boreal needleleaf forest	low to high shrub	53.465	122.345
Spiraea	pubescens	boreal needleleaf forest	low to high shrub	53.465	122.345
Vaccinium	vitis-idaea	boreal needleleaf forest	erect dwarf shrub	53.465	122.345
Vicia	sepium	boreal needleleaf forest	climber	53.465	122.345
Alnus	hirsuta	boreal needleleaf forest	tree	53.385	122.255

Avena?	fatua?	boreal needleleaf forest	graminoid	53.385	122.255
Betula	platyphylla	boreal needleleaf forest	tree	53.385	122.255
Calamagrostis	angustifolia	boreal needleleaf forest	graminoid	53.385	122.255
Geranium	wilfordii	boreal needleleaf forest	forb	53.385	122.255
Gymnocarpium	jessoense	tundra	pteridophyte	53.385	122.255
Larix	gmelinii	tundra	tree	53.385	122.255
Ledum	palustre	tundra	erect dwarf shrub	53.385	122.255
Maianthemum	bifolium	tundra	forb	53.385	122.255
Pinus	sylvestris var. mongolica	tundra	tree	53.385	122.255
Populus	davidiana	tundra	tree	53.385	122.255
Rhododendron	simsii	tundra	low to high shrub	53.385	122.255
Rosa	acicularis	tundra	low to high shrub	53.385	122.255
Rubus	arcticus	tundra	forb	53.385	122.255
Rubus	clivicola	tundra	forb	53.385	122.255
Salix	raddeana	tundra	low to high shrub	53.385	122.255
Saussurea	japonica	tundra	forb	53.385	122.255
Sorbaria	sorbifolia	tundra	low to high shrub	53.385	122.255
Paris	verticillata	tundra	forb	53.385	122.255
Vaccinium	vitis-idaea	tundra	erect dwarf shrub	53.385	122.255
Acer	oliverianum	boreal deciduous forest	tree	33.435	108.435

Anemone	hupehensis	boreal deciduous forest	forb	33.435	108.435
Carex	siderosticta	boreal deciduous forest	graminoid	33.435	108.435
Carpinus	tschonoskii	boreal deciduous forest	tree	33.435	108.435
Celastrus	orbiculatus	boreal deciduous forest	liana	33.435	108.435
Bothrycaryum	controversum	boreal deciduous forest	tree	33.435	108.435
Dendrobenthamia	japonica var. chinensis	boreal deciduous forest	tree	33.435	108.435
Elaeagnus	umbellata	boreal deciduous forest	low to high shrub	33.435	108.435
Epimedium	sagittatum	boreal deciduous forest	forb	33.435	108.435
Euonymus	alatus	boreal deciduous forest	low to high shrub	33.435	108.435
Sinarundinaria	nitida	boreal deciduous forest	bamboo	33.435	108.435
Hedera	nepalensis	boreal deciduous forest	liana	33.435	108.435
Holboellia	fargesii	boreal deciduous forest	liana	33.435	108.435
Ilex	pernyi	boreal deciduous forest	small tree	33.435	108.435

Juglans	cathayensis	boreal deciduous forest	tree	33.435	108.435
Kalopanax	septumlobus	boreal deciduous forest	tree	33.435	108.435
Lespedeza	buergeri	boreal deciduous forest	low to high shrub	33.435	108.435
Litsea	pungens	boreal deciduous forest	small tree	33.435	108.435
Neillia	sinensis	boreal deciduous forest	low to high shrub	33.435	108.435
Paederia	scandens	boreal deciduous forest	liana	33.435	108.435
Petasites	japonicus	boreal deciduous forest	forb	33.435	108.435
Pinus	armandii	boreal deciduous forest	tree	33.435	108.435
Pinus	tabuliformis	boreal deciduous forest	tree	33.435	108.435
Quercus	aliena var. acutiserrata	boreal deciduous forest	tree	33.435	108.435
Rodgersia	aesculifolia	tundra	forb	33.435	108.435
Schisandra	sphenanthera	tundra	liana	33.435	108.435
Smilax	discotis	boreal deciduous forest	liana	33.435	108.435
Smilax	stans	boreal deciduous forest	low to high shrub	33.435	108.435

Styrax	hemsleyanus	boreal deciduous forest	tree	33.435	108.435
Toxicodendron	vernicifluum	boreal deciduous forest	tree	33.435	108.435
Tsuga	chinensis	boreal deciduous forest	tree	33.435	108.435
Unknown fern		boreal deciduous forest	pteridophyte	33.435	108.435
Unknown grass		boreal deciduous forest	graminoid	33.435	108.435
